# Discovery of small molecule ligands for the von Hippel-Lindau (VHL) E3 ligase and their use as inhibitors and PROTAC degraders

**DOI:** 10.1039/d2cs00387b

**Published:** 2022-08-19

**Authors:** Claudia J. Diehl, Alessio Ciulli

**Affiliations:** Centre for Targeted Protein Degradation, School of Life Sciences, University of Dundee Dundee Scotland UK a.ciulli@dundee.ac.uk

## Abstract

The von Hippel-Lindau (VHL) Cullin RING E3 ligase is an essential enzyme in the ubiquitin-proteasome system that recruits substrates such as the hypoxia inducible factor for ubiquitination and subsequent proteasomal degradation. The ubiquitin-proteasome pathway can be hijacked toward non-native neo-substrate proteins using proteolysis targeting chimeras (PROTACs), bifunctional molecules designed to simultaneously bind to an E3 ligase and a target protein to induce target ubiquitination and degradation. The availability of high-quality small-molecule ligands with good binding affinity for E3 ligases is fundamental for PROTAC development. Lack of good E3 ligase ligands as starting points to develop PROTAC degraders was initially a stumbling block to the development of the field. Herein, the journey towards the design of small-molecule ligands binding to VHL is presented. We cover the structure-based design of VHL ligands, their application as inhibitors in their own right, and their implementation into rationally designed, potent PROTAC degraders of various target proteins. We highlight the key findings and learnings that have provided strong foundations for the remarkable development of targeted protein degradation, and that offer a blueprint for designing new ligands for E3 ligases beyond VHL.

## Abstract 2

This review provides a comprehensive overview of the structure-based design of small-molecule VHL ligands and their applications as VHL inhibitors and E3 ligase recruiting moieties in PROTAC degraders.

## Graphical abstract

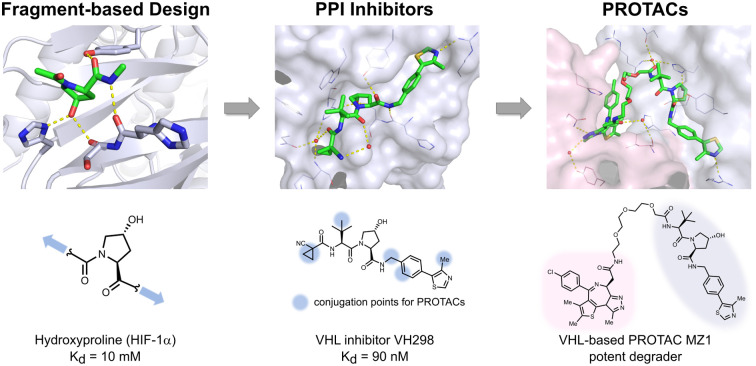

## 1. Introduction

**Fig. 1:**
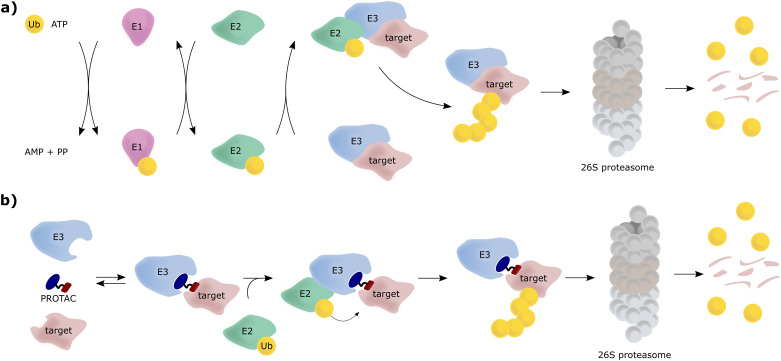
Schematic overview of the UPS, where E3 can consist of a single protein or a multi-subunit protein complex (a) and mechanism of action of PROTAC molecules inducing targeted protein degradation (b).

The von Hippel-Lindau (VHL) protein is the substrate receptor subunit of the Cullin2, really interesting new gene (RING)-VHL (CRL2^VHL^) multi-subunit E3 ligase. CRL2^VHL^ is a representative of the group of E3 ligases, which are, along with E1 and E2 ligases, essential enzymes of the ubiquitin–proteasome system (UPS), the cellular machinery responsible for degrading intracellular protein targets.^1–4^ In this cascade process, target proteins are tagged with the small protein ubiquitin, which is initially ATP-dependently activated by an E1 ubiquitin-activating enzyme, then transferred to an E2 ubiquitin-conjugating enzyme and eventually, covalently transferred to a substrate protein, a key step catalysed by an E3 ligase. Poly-ubiquitin chains can be built by E3 ligases, by transferring further ubiquitin molecules to the substrate-bound ubiquitin, and serve as recognition tags for the 26S proteasome, which unfolds and degrades poly-ubiquitylated proteins (Fig. 1a). As a consequence of their exquisite substrate specificity in the ubiquitination process, E3 ligases present attractive therapeutic targets, *e.g.*, through disruption or modulation of the interaction with their natural substrates.^5^ Hijacking of this UPS machinery for targeted protein degradation (TPD) to degrade non-natural neo-substrates has held promise for a long time. However, only in recent years TPD has established itself as a viable means of small-molecule intervention, mainly due to the success of proteolysis-targeting chimeras (PROTACs).^6–8^ PROTACs are hetero-bifunctional molecules, consisting of an E3 ligase ligand and a ligand for the target protein connected by a linker, and as such are designed to bring the target protein and the E3 ligase in proximity, resulting in ubiquitination of the target protein and its subsequent proteasomal degradation (Fig. 1b).

E3 ligases have been historically considered “undruggable” targets, because they are often large multi-subunit or multi-domain proteins, catalysing a ligation reaction across two proteins.^9^ As a result, the development of E3 ligase ligands has proven challenging, as E3 ligases lack ligandable active sites found in conventional druggable targets, for example protein kinases and G protein-coupled receptors (GPCRs). Binding to E3 ligases instead must involve targeting of protein–protein interactions (PPIs) or otherwise shallow protein surfaces or interfaces.^10^ This challenge proved a stumbling block for the PROTAC field for over a decade since its first inception in 2001.^11^ This changed shortly after high-quality, drug-like small-molecule binders for the E3 ligase substrate receptor subunits VHL and cereblon (CRBN) were reported and their binding modes crystallographically determined around 2012–2014.^12–15^ Beyond that, ligands for further E3 ligases such as mouse double minute 2 homologue (MDM2),^16^ cellular inhibitor of apoptosis (cIAP),^17^ RING-type zinc-finger proteins (RNF4, RNF114),^18,19^ damage-specific DNA binding protein 1 (DDB1)-Cul4 associated factor 11, 16 (DCAF11, DCAF16)^20,21^ or Kelch-like ECH-associated protein 1 (KEAP1),^22^ have been developed and also used as part of PROTAC molecules. Nonetheless, CRBN and VHL ligands remain the most successful and most widely E3 ligase recruiting ligands used in PROTACs.^23,24^

Herein, the journey leading to the structure-based design and optimisation of small-molecule VHL ligands is presented. We also discuss the various developments and diverse applications of such VHL binders – both as VHL inhibitors and as VHL-recruiting portions of PROTAC molecules.

## 2. The VHL gene and protein

The von Hippel–Lindau (*vhl*) gene was first identified in 1993, by positional cloning.^25^ It is associated with VHL disease, an autosomal dominant genetic disease caused by germline inactivating mutations in *vhl*, predisposing to various types of tumours, such as retinal angiomatosis and haemangioblastomas.^26,27^ These conditions had been known long before the *vhl* gene was discovered. Patients with familial retinal angiomatosis were first described in 1894 by Treacher Collins,^28^ and then in 1904 by Eugen von Hippel.^29^ In 1926, Arvid Lindau described central nervous system haemangioblastomas, that were linked to the retinal angiomas previously reported.^30^ The name von Hippel-Lindau disease was later coined and used to describe patients with retinal angiomatosis and cerebellar haemangioblastomas. Other cancers frequently related to VHL disease are clear-cell renal cell carcinomas (ccRCCs) and phaeochromocytomas (PCCs), amongst other tumours of the kidney, epididymis and pancreas.^31^ Biallelic inactivation of *vhl* is embryonic lethal in mice (*vhl*−/−) owing to placental vasculogenesis deficiency, whereas heterozygous *vhl* mice (*vhl*+/−) are viable. The *vhl* gene, located on chromosome 3p25, encodes two major protein isoforms: a 213 amino acids “long” isoform (VHL_1–213_), and a 160 amino acids “short” isoform (VHL_54–213_), that arises from an internal alternative translation initiation site (Met54) and lacks the N-terminal pentameric acidic repeat domain.^32,33^ These two isoforms, long and short, are often referred to as pVHL30 and pVHL19, respectively, based on their apparent molecular masses upon gel electrophoresis. Both isoforms exhibit tumour suppressor function, as shown in functional complementation studies in mice.^34^ Importantly, both isoforms are ubiquitously expressed, and exhibit E3 ligase activity to target hypoxia inducible factors (HIFs) as substrate for oxygen-dependent degradation.^35^

### 2.1. Structures and function of VHL

**Fig. 2:**
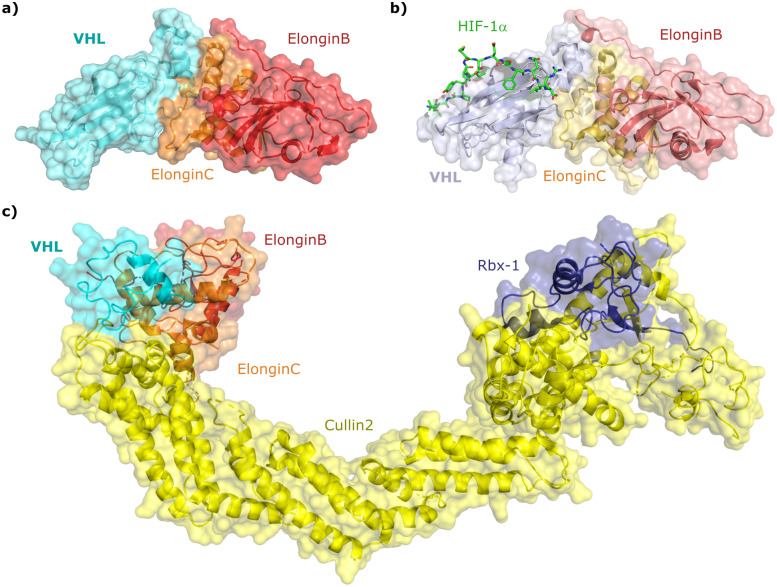
Crystal structure of VCB (PDB 1VCB) (a),^40^ cocrystal structure of HIF-1α peptide bound to VCB (PDB 1LM8) (b),^41^ and structure of the full length VCB-Cul2-Rbx1 multidomain complex (PDB 5N4W) (c).^42^

Since the late nineties, biochemical studies had shown that VHL associates with components of a Cullin RING ligase complex, later named CRL2^VHL^: adaptor subunits Elongin B (EloB) and Elongin C (EloC); scaffold subunit Cullin2 (Cul2), and the RING-containing protein Rbx1, that recruits a ubiquitin-loaded E2 to promote the transfer of ubiquitin to substrates.^36–38^ Within CRL2^VHL^, VHL interacts with the EloB/C adaptor subunits *via* a conserved sequence motif called the VHL/BC-box.^39^ The first crystal structure of VHL was solved in 1999 by the Pavletich laboratory, as a ternary complex with EloC and EloB, also named VCB (Fig. 2a).^40^ VHL is composed of two distinct domains: an N-terminal β domain (amino acid 63–154) that contains the binding site for the substrate hypoxia inducible factor 1 (HIF-1α), and an α domain (amino acid 155–213) that serves primarily to recruit ElonginB/C (Fig. 2a).

HIF-1α, and the paralogous HIF-2α, are the most well-characterised substrates of VHL. HIF-α proteins function as oxygen-sensitive subunits within the HIF transcription factors, to induce the expression of specific genes in response to low oxygen levels (hypoxia), including genes involved in cell proliferation and angiogenesis, and regulators of energy uptake and anaerobic metabolism.^43,44^ Under normal oxygen levels (normoxia), two conserved proline residues of HIF-α (Pro402 and Pro564 in HIF-1α) are hydroxylated by prolyl hydroxylase domain-containing (PHD) enzymes that use 2-oxoglutarate, Fe^2+^, and ascorbate as co-factors, and oxygen as co-substrate.^45,46^ Upon prolyl hydroxylation, HIF-α are recognised by VHL, poly-ubiquitylated and subsequently degraded by the proteasome. In contrast, at low oxygen levels (hypoxia), due to the insufficient oxygen level, PHDs are unable to efficiently hydroxylate HIF-α subunits. The non-hydroxylated HIF-α molecules are no longer recognised by VHL and escape ubiquitination–degradation, thereby accumulating and entering the nucleus where they dimerise with the HIF-β subunit and promote the transcription of hypoxia-responsive genes.^47^

Cocrystal structures of VCB bound to a HIF-1α peptide were solved and published in 2002 by the Pavletich and Jones laboratories.^41,48^ The structures elucidated the stereoselective recognition of hydroxylated proline Hyp564 by VHL within the C-terminal oxygen destruction domain (CODD) of HIF-1α, and specific interactions of the linear peptide epitope with the VHL β domain binding surface (Fig. 2b). Over a decade later, cocrystal structures of VCB with bound N-terminal domain of Cul2 solved by the Xiong laboratory,^49^ and of the full-length VHL-EloB/C-Cul2-Rbx1 pentameric complex solved by the Ciulli laboratory,^42^ illuminated the molecular recognition of recruitment of Cul2 by VCB and of the full CRL2^VHL^ complex components assembly (Fig. 2c). CRL2^VHL^ is activated by neddylation, a post-translational modification attaching the ubiquitin-like protein NEDD8 at a specific lysine residue on the C-terminal region of the Cullin subunit, inducing conformational changes and multivalent interactions that assist ubiquitin transfer to the substrate protein.^50,51^ Cul2 can be further de-neddylated and de-activated by a protease complex called the COP9 signalosome (CSN), and de-neddylated CRL2 is sequestered by Cullin-associated NEDD8-dissociated protein 1 (CAND1) that keeps the CRL in an inactive state.^52^

### 2.2. Early attempts to target VHL

In an early study attempting to intercept and interfere with normoxic HIF regulation, the Pugh group showed that polypeptides derived from the HIF-1α oxygen-dependent degradation domains (ODDs) around Pro402 and Pro564 could be ectopically expressed in cells to block the normoxic HIF degradation pathway leading to stabilisation of HIF-α and upregulation of downstream processes.^53^ VHL interaction assays suggested that these polypeptides are able to bind to VHL – presumably after their own prolyl hydroxylation – and act as competitor to HIF-1α as native substrate of VHL. However, these early efforts were not further developed, in part because they required genetic manipulation to express the desired peptides, and in part because of the limitations associated with peptidic molecules, such as poor drug-like properties, low cell permeability and low intracellular stability due to susceptibility to proteolytic cleavage, which prevented their use as chemical tools to study biology.

The concept of hijacking the UPS for targeted protein degradation using a bifunctional PROTAC molecule as tool to bring an E3 ligase and a target protein in close proximity, resulting in target polyubiquitination and proteasomal degradation, was first described in 2001 by the Crews and Deshaies laboratories.^11^ As proof-of-concept, the protein methionine aminopeptidase-2 (MetAP-2) was targeted and shown to be depleted in cell lysates upon addition of a bifunctional PROTAC, consisting of a 10-mer IκBα phosphopeptide as a ligand for the Skp1-Cullin-F box complex, at that time one of the best-studied E3 ligases, and a small-molecule ligand for MetAP-2.^11^ The Crews laboratory subsequently reported peptide-based VHL-recruiting PROTACs as chemical tools inducing degradation of green fluorescent protein (GFP) fused with the FK506 binding protein (FKBP12) and GFP fused with the androgen receptor (AR).^54^ Derived from the native substrate HIF-1α, a seven amino acid sequence ALAPYIP corresponding to the region around Pro564 of HIF-1α was exploited as a VHL recognition unit in those early PROTAC molecules. To address the lack of cell permeability of peptide-based PROTACs, a poly-arginine sequence originating from the cell-uptake of the transactivating transcriptional activator (Tat) protein was added as a cell-penetrating tag to the peptide sequence.^54^ These early studies, while pioneering in efforts, highlighted the limitations and challenges associated with the peptidic nature of the E3 ligase binding moieties available at the time, thus motivating the quest for non-peptidic, more drug-like ligands.

## 3. Structure-based design of small-molecule VHL inhibitors

Spurred by the promise of PROTAC technology and its potential applications, the search began for novel E3 ligase binders. The applicability of peptidic E3 ligase binders in cells and *in vivo* remained limited, because of the poor physicochemical properties of peptides, as described above. Despite this, peptidic binders offer useful tools to target PPIs, which is required to target E3 ligases, and can be optimised to have high affinity and excellent selectivity for their target binding site.^55–57^

Non-peptidic binders are usually small-molecular scaffolds with an average molecular weight <500 Da, thus featuring desirable drug-like properties. Physicochemical properties and bioactivity of small-molecule inhibitors can be finely modulated by tuning their molecular structure, *e.g.*, by introducing lipophilic groups, controlling the number of hydrogen bond donors (HBD) or subtly changing their electronics through variation of substituents. However, due to their considerably smaller size and surface area compared to peptidic binders, it is challenging for non-peptidic small molecules to successfully target proteins at shallow surfaces and PPIs – that are found outside of active sites. Consequently, when targeting PPIs, small-molecule non-peptidic inhibitors often feature lower affinity and reduced target selectivity compared to their peptidic counterparts.^10,58,59^ Ideally, a small-molecule E3 ligase binder should have good physicochemical properties while maintaining high target binding affinity, selectivity and cellular activity.

### 3.1. First-generation VHL inhibitors

**Fig. 3:**
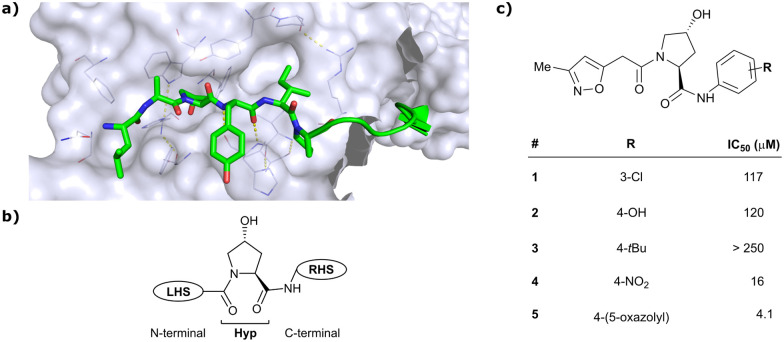
Initial structure-guided design of VHL inhibitors. Cocrystal structure of hydroxy-HIF-1α (green) bound to VHL (PDB 1LM8)^41^ (a), nomenclature of VHL inhibitor subsections (b) and first reported binders (c).^12^

As both high-throughput screening (HTS) and virtual screening approaches were initially unsuccessful in identifying *bona fide* VHL binders, researchers from the laboratories of Ciulli and Crews turned to nature for a starting point for rational design of a small-molecule ligand of VHL.^12^ They targeted the known binding site of VHL's native substrate, hydroxy-HIF-1α, on the VHL protein surface by attempting to mimic the critical PPIs of hydroxy-HIF-1α with VHL as observed from cocrystal structures (Fig. 3a).^41^ Based on the essential element in hydroxy-HIF-1α recognition by VHL, the hydroxyproline Hyp564, which originates from post-translational hydroxylation of Pro564, was elected as the initial central motif for *de novo* elaboration of hydroxyproline (Hyp) derivatives.^12^

The inhibitor design efforts were based on extending the molecular scaffold to both sides of this central Hyp fragment, dissecting the hydroxy-HIF-1α binding site of VHL into a left-hand side (LHS) and right-hand side (RHS) relative to Hyp (left and right are depicted from the N- to C-terminus of the bound peptide, see Fig. 3b). Structural optimisation at the LHS proceeds *via* N-terminal modification of the central Hyp, while the C-terminus of Hyp is targeted during RHS optimisation (Fig. 3b). The central Hyp binding site is formed by buried, mostly aromatic, side chains of VHL, such as Trp88, Tyr98, His110, Ser111, His115 and Trp117. Upon Hyp binding, in its C^4^-*exo* conformer stabilised by the presence of the C^4^-hydroxy group, key hydrogen bonds are formed between Hyp's hydroxyl group and the side chains of Ser 111 and His115, between Hyp's carbonyl and the hydroxy group of Tyr98 and between Hyp's amide -NH and the backbone carbonyl of His110. The LHS pocket is confined by hydrophobic residues of Phe91 and Tyr112 and the hydrophilic amino acids Asn67, Arg69 and His115. On the RHS, a hydrophobic elongated pocket is enclosed by Phe76, Tyr98, Ile109 and Trp117 close to the Hyp site and Pro86, Pro99, Leu101 and Arg107 further to the RHS.

**Fig. 4:**
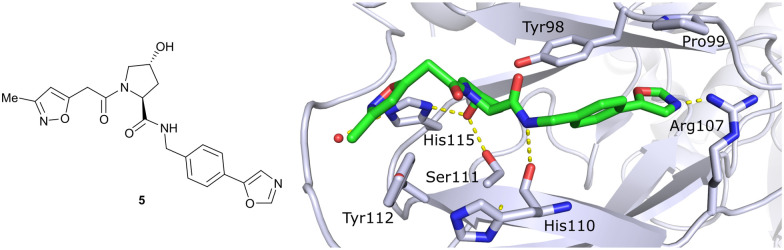
Cocrystal structure of 5 bound to VCB (PDB 3ZRC).^12^

*In silico* predictions identified an isoxazole moiety as a promising design element for engaging favourably with a crystallographic water bound at the LHS pocket. Addition of a methyl-isoxazole group to the Hyp amino acid yielded a minimal binding pharmacophore as identified by studying binding of Hyp-containing fragments using water-ligand observed *via* gradient spectroscopy (WaterLOGSY) NMR binding experiments.^12^ Accordingly, Hyp-derivatives 1 and 2 were synthesised featuring a LHS isoxazole unit and a RHS benzyl group envisioned to interact with the side chain of Tyr98. The potential binding ability of these initial compounds to VHL was evaluated with a fluorescence polarisation (FP) displacement assay using a fluorescently labelled HIF-1α peptide, FAM-DEALA-Hyp-YIDP, as probe. Confirming the design strategy, both initial compounds were able to displace the probe and qualified as VHL-binders, though probe displacement occurred only at high concentrations (half maximal inhibitory concentration (IC_50_) >100 μM) (Fig. 3c). Using a solid-phase-synthesis protocol, a library of 15 analogues of 1 introducing differently 4-substituted benzyl amines on the RHS was synthesised and evaluated. As a general trend, higher binding affinity was detected with electron withdrawing groups (EWG) in the *para*-position (*e.g.* -NO_2_, 4), while electron donating groups (EDG) reduced binding affinity (*e.g.* -*t*Bu, 3). The best binder 5, featuring an oxazole ring in 4-position, achieved a single-digit μM IC_50_ value – an almost 28-fold increase in binding affinity compared to compound 1. The cocrystal structure of 5 in complex with VCB confirmed its binding site on VHL and its predicted binding mode compared to the HIF-1α peptide. The key hydrogen bonding interactions around Hyp564 were preserved, including hydrogen bonds between the hydroxy group of Hyp and Ser111 and His115, as well as interactions between the RHS carbonyl and the phenolic -OH of Tyr98, and between the RHS amide -NH and His110 (Fig. 4). Inhibitor 5 forms a hydrogen bond between the LHS isoxazole with a crystallographically conserved water in the LHS pocket, as designed, and a hydrogen bond between the RHS oxazole group and the Arg107 side chain. Additional favourable hydrophobic interactions are formed with Pro99 as well as π–π-interactions of the LHS isoxazole with Tyr112 and – as envisioned – of the RHS benzylic group with Tyr98. This side-on π–π-interaction of the RHS benzylic moiety with the electron-rich phenolic ring of Tyr98 rationalised the observed favourability of electron-poor arenes at the RHS as opposed to electron-rich ones.

**Fig. 5:**
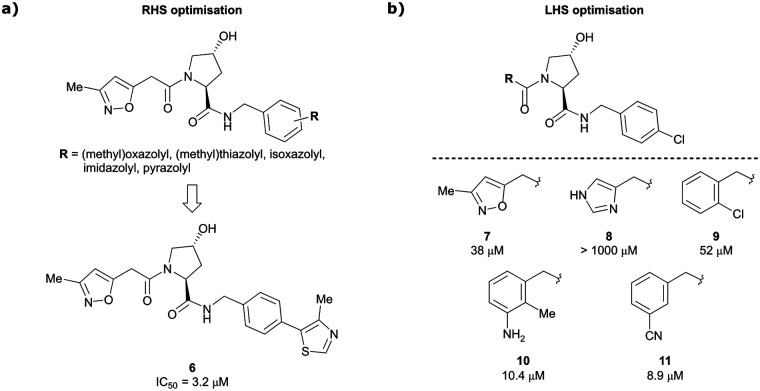
Structure-guided optimisation of the RHS (a) and LHS (b) in Hyp-based inhibitors.^13^

Building on this initial success, a detailed follow-up study by the Ciulli and Crews laboratories initially optimised LHS and RHS of Hyp-based VHL binders separately to assess VHL ligand structure–activity relationships (SAR) followed by a combinatorial optimisation of both sides using initial hits from the side-separated optimisations.^13^ Extending the previous SAR of the RHS, the effect of 5-membered heterocyclic moieties as substituents in *para*-position of the benzyl fragment on the binding affinity was systematically investigated. Therefore, a variety of N-, O-, and S-containing 5-membered rings were tested, but oxazole 5 showed superior binding affinity to all other examined derivatives. Aiming at increasing the hydrophobic contact with Pro99, an additional methyl group was introduced at the 4-position of oxazole and thiazole rings, leading to discovery of the slightly more potent 4-methylthiazole compound 6 (IC_50_ = 3.2 μM) (Fig. 5a).

For the synthetic ease of the LHS optimisation, 4-chlorophenyl was chosen as RHS fragment and a set of heteroaryl acetamides was screened. All of these derivatives – even pyrazole and imidazole analogues (*e.g.*, 8) that were expected to interact more tightly with the LHS structural water – had lower affinity than reference compound 7. In a more diverse screening set, chlorobenzamide derivative **9** featured only a 1.4-fold loss in binding affinity compared to 7 (Fig. 5b). Based on this result, a range of benzamide derivatives were designed and several improved binders were obtained, such as 3-amino-2-methylbenzamide derivative 10 (IC_50_ = 10.4 μM) and 3-cyanobenzamide 11 (IC_50_ = 8.9 μM).

**Fig. 6:**
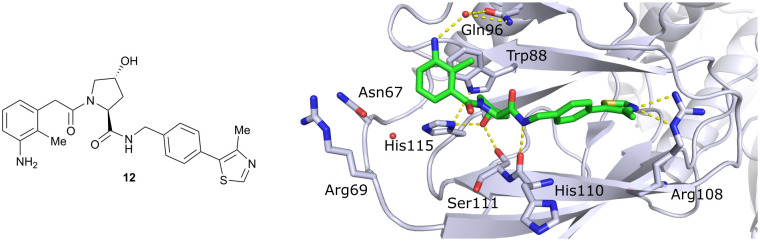
Cocrystal structure of inhibitor 12 bound to VCB (PDB 4B9K), derived from combinatorial optimisation of LHS and RHS.^13^

Finally, combinatorial optimisation by screening a set of LHS benzamides against *para*-chloro-, *para*-oxazolyl- and *para*-(4-methy)thiazolyl benzyl moieties on the RHS resulted in improved binding. Inhibitor 12 combines the most potent fragments of the separate optimisations, the 3-amino-2-methylbenzamide moiety on the LHS and the (4-methy)thiazolyl benzyl fragment on the RHS. The cocrystal structure of 12 bound to VCB was solved at 2.00 Å resolution.^13^ The bound conformation of the RHS of 12 to VCB resembles the binding mode of the previously reported oxazolyl fragment of 5, while better filling the hydrophobic pocket underneath Pro99 with the methyl-thiazole ring (Fig. 6). Compared to 5, inhibitor 12 adopts an alternative LHS conformation by avoiding the structural water in the pocket bound by Asn67, Arg69 and His115, and orientating towards the side chain of Trp88, thereby creating a novel water-mediated hydrogen bond to the side chain of Gln96.

Based on these first-generation inhibitors, a systematic study explored the potential of fragment-based lead discovery (FBLD) for targeting the VHL–HIF protein–protein interface.^60^ Dividing the known binders into rule-of-3 (Ro3) compliant fragments did not result in detectable binding to VHL using a range of biophysical techniques including differential scanning fluorimetry (DSF), FP, and isothermal titration calorimetry (ITC). Binding of zwitterionic amino-acid Hyp as Ro3-compliant fragment could not be detected and required capping of the charged amino and carboxylate functionalities with acetyl and methyl-amide, respectively, to enable detection of binding. Nonetheless, binding of capped Hyp still remained remarkably weak (dissociation constant (*K*_d_) ∼ 10 mM), and required significant optimisation of the detection limits in all biophysical assays used. These observations likely rationalise previous failure to identify VHL inhibitors using HTS and virtual screening strategies.

By enlarging the fragments of the initial binder beyond Ro3, detection of binding events using biophysical assays became possible. A small set of functionalities on the RHS and LHS of the Hyp core was assessed for their ligand efficiency (LE) and lipophilic ligand efficiency (LLE). While the RHS pocket tolerates a range of aromatic and heteroaromatic rings as *para*-substituents of the benzyl moiety, a *tert*-butyl (*t*Bu) group was identified as a novel favourable feature at the LHS. This *t*Bu group became very important in the design of second-generation VHL ligands, detailed next.

### 3.2. Second-generation VHL inhibitors

Despite the successes in designing the first-generation VHL binders, these molecules still featured only a moderate binding potency in the single-digit micromolar range, low lipophilicity potentially limiting cell permeability, and as a result lacked cellular activity.^13^ Striving towards improved binding affinity and lipophilicity, the Ciulli laboratory followed a metrics-, structure- and ITC-guided design strategy for VHL inhibitors in the following years.^14,61,62^

**Fig. 7:**
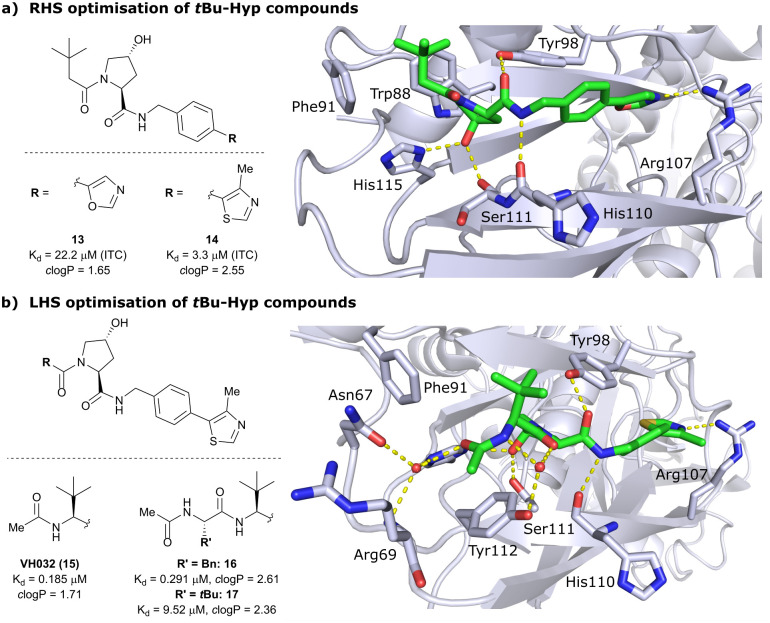
Development of second-generation VHL inhibitors: RHS optimisation starting from benchmarking compound 13 and cocrystal structure of 13 with VCB (PDB 4W9C) (a); disclosure of VHL inhibitor VH032 in the course of LHS optimisation and cocrystal structure of VH032 bound to VCB (PDB 4W9H) (b).^14^

As the previous FBLD study^60^ disclosed a *t*Bu functionality on the LHS α-position as highly beneficial, further optimisation efforts were started from a *t*Bu-Hyp fragment featuring a more balanced lipophilicity compared to the hydrophilic isoxazole-Hyp as an anchor ligand. As a benchmarking compound, ligand 13 was initially synthesised and thoroughly characterised.^14^ Crystal structure elucidation of 13 bound to VCB revealed maintained key interactions at the Hyp core and a novel LHS orientation, with the *t*Bu group pointing upwards to form hydrophobic contacts to Phe91 and Trp88 (Fig. 7a).

Structural superposition of the ligated VHL protomers (four within the asymmetric unit of the crystal lattice) showed considerable variation in the dihedral angles of C_phenyl_–C_oxazolyl_. This observation, together with conformation energy calculations revealing a preferred energy minimum of 0° for the dihedral angle between phenyl and oxazolyl, indicated energetic unfavourability of the bound conformation of 13, likely accounting for the only moderate binding affinity (*K*_d_ = 22 μM by ITC). This analysis prompted further optimisation to lock the RHS biaryl in a more favourable bound conformation. Derivatisation at the *para*-position of the RHS benzylic moiety by introducing 5-membered oxazole and thiazole rings led to a small series of binders with overall improved lipophilicities. Methylation of the 4-position of oxazolyl- and thiazolyl moieties improved both binding affinity and lipophilicity and locked the dihedral angle to a more favourable ∼40° energy minimum that was observed consistently in all protomers of the bound cocrystal structures that were solved for each compound. Together, this strategy generated the most potent binder of this series, 4-methylthiazole analogues 14 with a *K*_d_ of 3.3 μM. Introduction of a methyl group as *meta*-substituent on the benzyl ring was tolerated but did not improve the binding potency.

For further LHS optimisation, an amide bond vector into the LHS pocket was envisioned as attractive growing vector, thereby mimicking the peptidic backbone structure of HIF-1α. By using *N*-Boc-protected l-*tert*-leucine (*tert*-Leu) as reactant for amide-coupling with the LHS of Hyp, a terminal amine group required by the peptidic growing vector was easily introduced. N-terminal acetylation gave rise to the considerably improved VHL-inhibitor VH032 (15), with a *K*_d_ of 185 nM, yielding the first small-molecule inhibitor featuring a higher binding affinity towards VHL than the 10-mer model HIF-1α peptide. VH032 retains the previous binding interactions on the RHS of Hyp, and gains in binding affinity by beneficial hydrogen bonding of the newly introduced amide group with a structural water in the LHS pocket. The amide -NH is facing towards the solvent, thereby avoiding adverse interactions with VHL's protein surface (Fig. 7b). This amide -NH is potentially also shielded and so minimises desolvation penalties, due to the steric bulk offered by the *tert*-Leu side chain group, an effect later observed with macrocyclic peptidic compounds.^63^ To this end, the *t*Bu group proved to be essential in affording the superior VHL binding affinity of VH032, as exchange of *tert*-Leu with other acetylated natural and non-natural amino acids, *e.g.* Pro, Hyp or phenylglycine, led to about 3.5- to 5-fold reduced binding affinity. Further growing into the LHS pocket by addition of another amino acid (Ala, Leu, Phe, *tert*-Leu, phenylglycine) did not improve the binding affinity compared to VH032, although nanomolar affinities were observed with the Leu and Phe (16) extensions. In contrast, direct attachment of bulky groups on the peptidic backbone led to a drastic loss in binding affinity (*e.g.*, *K*_d_ = 9.5 μM with *tert*-Leu; 17) (Fig. 7b).

From the structural insights of the cocrystal structure of VH032 bound to VCB, a systematic group-based optimisation strategy was pursued to further improve binding affinity, cell membrane passive permeability as well as cellular activity.^62^ Focussing on the LHS acetyl capping group of VH032, the carbonyl functionality introduced with *tert*-Leu was retained to capitalise on its hydrogen bonding interaction with structural water in the LHS pocket. To improve filling of the LHS pocket, that is shaped by Arg69, Asn67, Phe91 and Tyr112, the H-atoms of the LHS acetamide's methyl group were gradually replaced by larger groups. Furthermore, locking of the acetamide's conformation was attempted by addition of EWGs in α-position to the carbonyl, an effect well understood with model compounds.^64,65^ Moreover, the HBD (acetamide-NH) was replaced as a strategy to improve cellular permeability. The novel binders were evaluated not only biophysically by FP and ITC as in previous VHL inhibitor optimisations, but also in HeLa cells by monitoring HIF-1α protein levels to assess their cellular activity.

**Fig. 8:**
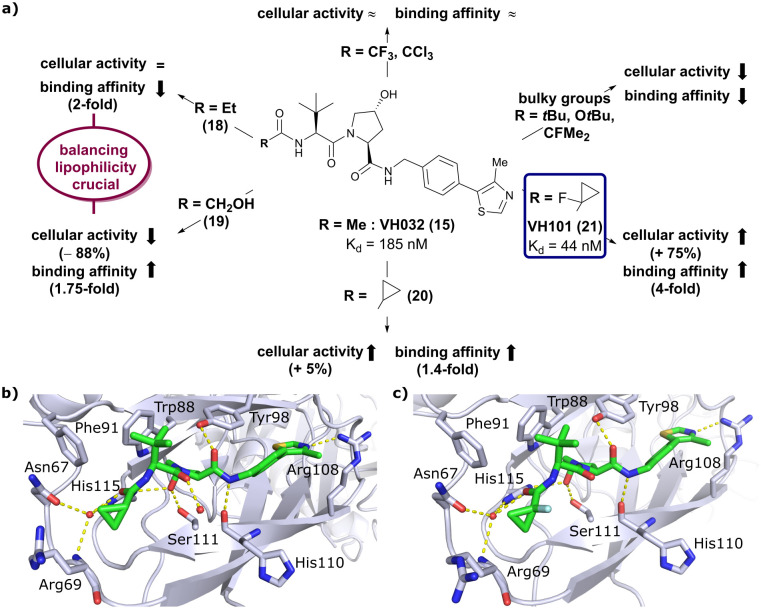
Development of second generation VHL inhibitors: structure–activity relationships at the LHS pocket (a), cocrystal structure of inhibitor 20 bound to VCB (PDB 5NVW) (b), and cocrystal structure of VH101 bound to VCB (PDB 5NVX) (c).^62^

Installation of bulky groups, such as *t*Bu, CFMe_2_ or *N*-Boc protection instead of acetylation, caused significant loss both in cellular activity and binding affinity as compared to VH032 (Fig. 8a), indicating that such groups were too large and could not be accommodated by the limited volume of the LHS pocket. Exchanging the methyl group with –CF_3_ or –CCl_3_ did not affect cellular activity or binding potency notably. Addition of an ethyl group maintained the cellular activity while leading to a 2-fold loss in binding affinity (compound 18), while hydroxylation led to 88% reduction in cellular activity despite increasing the binding affinity to VHL by 1.75-fold (compound 19). These two examples featuring inverse trends in cellular activity and binding affinity highlight the crucial impact of lipophilicity on cellular activity and consequently the necessity to evaluate lipophilicities of potential binders as part of inhibitor design strategies.

Replacement of the VH032 LHS methyl group with a highly constrained cyclopropyl ring – thus choosing a substituent's volume in between the too large *t*Bu group and the tolerated Et group while constraining its flexibility – proved to be beneficial, as binding affinity increased about 1.4-fold while marginally increasing cellular activity (compound 20). The addition of an α-fluorine substituent in combination with cyclopropylation generated VHL-inhibitor VH101 (21) with unprecedented activity, showing a 75% increased cellular potency and 4-fold increased binding affinity with a *K*_d_ value of 44 nM to VHL as compared to VH032.

Structurally, the cyclopropyl-containing inhibitors 20 and 21 both conserve the binding mode of VH032 at the VHL/HIF-1α interface including key interactions at Hyp and the inhibitors’ RHS. The cyclopropyl group fits snugly into the pocket on the far LHS and induces in both cases conformational changes in the Arg69 side chain, which bends to better accommodate the cyclopropyl moiety inside the LHS pocket (Fig. 8b and c). The α-fluorination in VH101 induces a strict anti-conformation of the α-fluorocarbonyl moiety. This favourable pre-organisation of the ligand results in a lowered entropic penalty for binding and therefore higher binding affinity. Further assessment of the cytotoxicity of VH101 revealed a notable decrease in cell proliferation on different cancerous and non-cancerous cell lines at 150 μM concentration of VH101. Cytotoxicity at these concentrations was also observed for its non-binding negative control *cis*-VH101, in which the *cis*-Hyp core motif prevents binding to VHL, indicating potential off-target cytotoxicity of VH101.^62^ For these reasons, VH101 did not qualify as a useful chemical probe and a second series of inhibitors was designed based on the SAR insights of VH101.

**Fig. 9:**
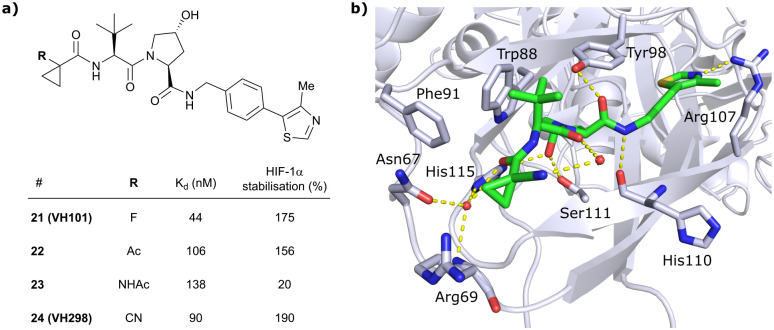
Development of 2^nd^ generation VHL inhibitors: further LHS optimisation towards reduced cytotoxicity (a) and cocrystal structure of inhibitor VH298 (24) bound to VCB (PDB 5LLI) (b).^62^

In this second inhibitor series, the observed flexibility in the side chain of Arg69 was further exploited by exchanging the cyclopropyl ring with slightly larger and more lipophilic groups. Replacement of the cyclopropyl by a cyclobutyl moiety led to a slightly reduced binding affinity as compared to VH032 while increasing its cellular potency about 1.4-fold. In direct comparison to cyclopropyl derivative 20, the one-carbon ring expansion resulted in 1.6-fold loss in binding affinity but increased cellular activity, indicating that lipophilicity drives the inhibitors’ cellular potency. Oxetane, cyclobutanone and *N*-acetylated azetidine incorporation was unfavourable for cellular activity and binding potency, defining a very narrow window of tolerated structural changes to the LHS cyclopropyl motif. Further LHS optimisation based on VH101 towards reduced cytotoxicity focused on replacing the α-fluorine atom on the LHS by other small EWGs, or by promoting intramolecular hydrogen bonds (Fig. 9a). Acetylation in α-position (22) slightly reduced the binding affinity compared to **VH101** (*K*_d_ = 106 nM *vs.* 44 nM, respectively), but still induced a considerable 1.5-fold increase in cellular activity as compared to VH032. Acetamide introduction (23) resulted in a 5-fold loss in cellular potency relative to VH032, attributed to unfavourable addition of an extra amide bond increasing the desolvation penalty and reducing cell permeability. Furthermore, no additional interaction with VHL was exploited through this acetamide functionality.^62^ Eventually, replacing the α-fluorine with a cyano group gave rise to VHL inhibitor VH298 (24) with competitive cellular activity compared to VH101 (1.9-fold increased relative to VH032) and a double-digit nanomolar binding affinity (*K*_d_ = 90 nM). Of all second-series inhibitors, VH298's binding mode recapitulates that of VH101 the most, featuring the same *trans*-orientation of the LHS amide bond with the cyano group pointing away from the protein surface and maintaining all previous identified stabilising interactions with VHL. Additionally, the cyano group engages in formation of a stabilising water network by forming a hydrogen bond to a structural water located above His115 (Fig. 9b). VH298 showed negligible cell toxicity at 150 μM concentration, thus presenting a significant improvement to VH101 and qualifying as feasible chemical probe candidate.

Analysing the second-generation VHL inhibitors SAR in more detail, strong correlation of inhibitor permeability, lipophilicity, binding affinity, and complex half-life with cellular activity were found. In contrast, neither the number of HBD or rotatable bonds correlated with cellular activity in general, except a preference for HBDs ≤ 3. Nonetheless, both factors showed significant influence in isolated cases, *e.g.*, inhibitors 19 and 23, and should not be neglected in inhibitor design.

### 3.3. Qualification of VH298 as a VHL chemical probe

To evaluate the suitability of VH298 as chemical probe molecule able to displace HIF-1α from VHL, VH298 was systematically profiled in cells in comparison to its negative control, *cis*-VH298, which lacks binding affinity to VHL due to inverse stereochemistry at the essential hydroxy group of Hyp.^61^ Target engagement of VH298 was validated using cellular thermal shift assay (CETSA) and chemoproteomic analysis as orthogonal methods, while negligible off-target effects were observed at 50 μM concentration against >100 tested cellular kinases, GPCRs and ion channels. Both VH298 and VH032 showed no cell toxicity in several fibroblast, tumoural and non-tumoural cell lines at up to 150 μM (or even 500 μM) concentration, qualifying them as suitable chemical probes selectively targeting the VHL/HIF-1α interaction without global affection of cell viability.^61^ Further encouraging for *in vivo* application in animal models, VH298 showed slow microsomal clearance and high plasma metabolic stability.^62^

Down-stream effects of VH298 were studied using time- and dose-dependent treatments in HeLa cells followed by immunoblotting monitoring HIF-1α, HIF-2α and hydroxy-HIF-1α protein levels. These cellular activity studies revealed fast and long-lasting accumulation of HIF proteins in HeLa cells, a detectable response measurable at 10 μM inhibitor concentration and no response to the negative control *cis*-VH298, suggesting that the inhibitor's activity depends on VHL binding and is therefore on-target.^61^ Immunoprecipitation with a hydroxy-HIF-1α selective antibody showed that all stabilised HIF-1α is hydroxylated after treatments with VH298, consequently VH298 is interrupting the HIF-1α degradation pathway downstream from hydroxylation, as expected for an effective VHL/HIF-1α interaction disruptor. Monitoring of mRNA levels of known HIF target genes after treatments with VH298 and VH032 showed that the stabilised hydroxy-HIF-1α is transcriptionally active, as upregulation of HIF target genes was observed after treatments with VH032 and VH298.^61^

These initial assessments on downstream effects of the VHL inhibitors VH032 and VH298 determined that concentrations above 10 μM were required to ensure full target engagement and effective blockade of HIF-1α recruitment and ubiquitination by VHL, preventing subsequent proteasomal degradation. This means that large concentration ranges may be used for PROTAC applications where the VHL ligand is conjugated to POI ligands to induce POI degradation, without inducing undesirable down-stream effects from HIF stabilisation activity.

Beyond qualification as chemical probe by featuring selective and efficient on-target activity, a compound's metabolic stability is an additional qualifying factor of relevance for *in vivo* and therapeutic applications. While no metabolic studies have been published on VHL inhibitors, several soft spots for metabolism in the structure of VH032-derived ligands have been identified in the context of a PROTAC metabolic stability study.^66^ MS-based metabolite analysis identified aliphatic oxidation of carbons of the *t*Bu group and the Hyp ring, amide hydrolysis, glucuronidation of the hydroxy group of Hyp and human aldehyde oxidase-catalysed oxidation of the 2-position of the thiazole ring as prominent metabolic pathways. The impact of these metabolic modifications on inhibition potency or binary binding affinity has not been explored so far, but Hyp-glucuronidation will certainly impede binding to VHL, and hydroxylated metabolites would be expected to feature poorer cell permeability, thus weakening cellular potency.

### 3.4. Applications of VH298 as VHL inhibitor

Acknowledging VH298's qualification as VHL inhibitor by disrupting the VHL:HIF-α protein–protein interaction, VH298 has been included in the records of the chemical probes portal††https://www.chemicalprobes.org/vh298. and – along with its negative control – has been commercially available since 2017. VH032 and VH298 as chemical inhibitors of VHL have been used in –omics studies and compared to natural hypoxic response and response to PHD inhibitors.^67,68^ In a global transcriptomic analysis, unbiased high-throughput RNA sequencing was used to determine effects of treatment with VHL inhibitors VH032 and VH298, PHD inhibitors and hypoxia on the gene expression response.^67^ A large overlap in upregulated genes in all three experimental sets was observed, that indicated activation of the hypoxia signalling pathway *via* HIF transcription factors for all three cases. Furthermore, transfer of changed transcript levels of known HIF-target genes to protein levels was observed with VH298, which induced more hypoxia-related genes than VH032 underlining its higher potency.^67^ Using quantitative tandem mass tag (TMT) labelling-based mass spectrometry (MS), changes of the global proteome after treatment with the VHL inhibitors VH032 and VH298 were investigated and compared to hypoxia and treatment with a PHD inhibitor.^68^ Consistent with the previous transcriptomic analysis, the majority of upregulated proteins induced by the VHL inhibitors were also upregulated with the PHD inhibitor and in hypoxia. Notably, only the VHL inhibitors led to specific upregulation of VHL protein levels and enhanced proteasomal degradation of HIF-1α in prolonged treatments. This negative feedback mechanism prevents excess levels of HIF transcription factors upon prolonged inhibitor treatments, and is expected to result in low side effects of such VHL inhibitors in potential therapeutic applications.^68^

Based on its utility as HIF-1α activator, VH298 has been applied as a benchmark and control compound in various studies to develop a hypoxia response or more generally for studying the role of hypoxia in biology.^69–73^ The therapeutic potential of VH298 as HIF-1 activator has been explored in first *in vivo* studies aiming at enhancing enthesis healing after tendon injuries^74^ and improving hyperglycaemic wound healing.^75^ As the HIF-1 transcription factor enhances proliferation and accelerates differentiation of several cell types, accumulation of HIF-1α and hydroxy-HIF-1α through treatment with VH298 was hypothesised to accelerate healing of injured tendon-bone interface.^74^ At the cellular level, treatment of tendon-derived stem cells (TDSCs) with VH298 led to accumulation of HIF-1α and hydroxy-HIF-1α, improved viability and enhanced chondrogenic differentiation potential of TDSCs. In rat Achilles tendon-calcaneus rupture *in vivo* model, quickened maturation of enthesis tissue and improved healing was observed after post-injury treatment with VH298.^74^ Hyperglycaemia has been shown to impair HIF-1α protection under hypoxia and HIF-1α expression is decreased in skin wounds of diabetic rats. Consequently, the effect of VH298 on functional activities of fibroblasts and on wound healing processes in hyperglycaemic rat models has been established.^75^ As expected, VH298 induced increased protein levels of HIF-1α, HIF-2α and hydroxy-HIF-1α in rat fibroblasts and led to upregulation of mRNA of essential factors for wound healing. Furthermore, faster post-operative wound healing was observed in diabetic rats treated with VH298.^75^

These studies showcase not only the versatility of VH298 as means to induce hypoxia within cells, but also broad therapeutic potential of VH298 and VHL inhibitors more generally as HIF-1α activators.

### 3.5. Further applications of VHL inhibitors of generations 1 and 2

Apart from the use of the VHL inhibitors VH032 and VH298 as chemical probes, molecular scaffolds derived from the first and second generation VHL inhibitors have found applications in fragment screening optimisation^76^ and bioassay development^77–79^ as tool to investigate binding to VHL.

In a retrospective approach, defragmenting known first generation VHL inhibitors in Ro3-compliant fragments was used to assess the potential of NMR fragment screening towards druggability of VHL as a representative PPI target. Under ‘standard’ fragment screening conditions, none of these fragments showed detectable binding to VCB. A 3- to 4-fold increase of the concentrations of both ligand and protein led to detection of binding for about 40% of the Ro3-compliant compounds,^76^ suggesting that NMR fragment screening can be used to target PPIs, and that revision of standard active-site targeting conditions is important for success.

**Fig. 10:**
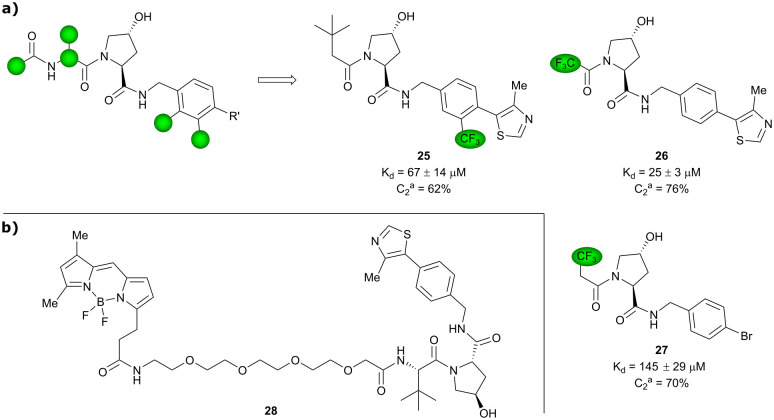
^19^F spy molecules for binding in Hyp site of VHL (a)^77^ and BODIPY-FL-PEG4-VH032 fluorescent probe (b).^79^

Based on VH032 as a template structure, the Ciulli laboratory developed a set of fluorine-containing VHL ligand analogues and assessed their utility as ^19^F NMR spy molecules for the Hyp binding site of VHL. Trifluoromethylated groups were attached at different positions of the inhibitor scaffold (Fig. 10) and the spy molecules’ binding affinities were evaluated by surface plasmon resonance (SPR) and related to their R_2_ contrast (*C*_2_) ^19^F NMR, the critical detection readout that is a measure of the change in R_2_ upon binding. The highest sensitivity was observed for compounds 25, 26 and 27 with *C*_2_ value between 62–76%, with 27 having the lowest binding affinity (*K*_d_ = 145 μM), thus the highest displacement rate in competition experiments (Fig. 10a).^77^ Consequently, spy molecule 27 was used to design a ^19^F NMR displacement assay, in which competitive ligand-observed ^19^F NMR was used to determine the binding affinity of VHL inhibitors^77^ and for the estimation of cooperativity factors of VHL-based PROTACs.^78^

The ^19^F competition assay using spy molecule 27 provided K_i_ values in good agreement with SPR-derived *K*_d_ values for VHL inhibitors.^77^ Robust differentiation between positive and negative cooperativity (*α*) was achieved as well as good correlation of cooperativity trends in comparison to orthogonal biophysical methods.^78^ Although *α* values of highly cooperative ternary complexes were underestimated and off-target interactions of the spy molecule with the protein of interest (POI) should be taken into consideration,^78^ this ^19^F displacement assay provides overall a valuable semi-quantitative tool to rapidly estimate cooperativities of VHL-recruiting PROTACs.

Popular high-throughput methodologies to assess the binding affinity of potential inhibitors are FP and time-resolved fluorescence resonance energy-transfer (TR-FRET) assays. As an alternative to the commonly used fluorescently labelled HIF-1α decapeptide, the Chen laboratory developed a small-molecule based high-affinity VHL fluorescent probe by linking the BODIPY FL fluorophore *via* a PEG_4_ linker to VH032 (Fig. 10b).^79^ The suitability of BODIPY-FL-PEG4-VH032 fluorescent probe (28) as surrogate for the fluorescently labelled HIF-1α decapeptide was established. Furthermore, using this fluorescent probe 28 a highly sensitive and selective TR-FRET assay was developed, validated against a set of VHL binders and applied into a pilot screening of >2000 alpha-helix mimetic small molecules for VHL inhibition hit identification.^79^

### 3.6. Efforts to expand the chemical space: fluoro-hydroxyprolines

Both HTS and fragment screening approaches have proven unsuccessful to date at identifying alternative binders for the Hyp site of VHL. However, fragment screenings by the Ciulli laboratory led to identification of two new pockets on the surface of the VCB protein – namely a second pocket on VHL distant from the HIF-1 Hyp binding site, and a pocket on EloC that engages with the N-terminal tail of Cul2.^80^ Consequently, synthetic efforts to further improve binding to VHL focused on modification to the Hyp core of previously established second-generation VHL inhibitors.

**Fig. 11:**
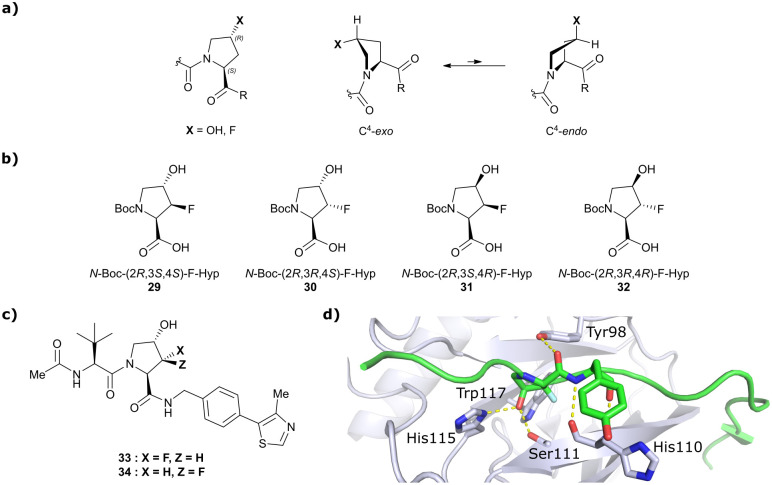
Pyrrolidine's conformational preference in 4-functionalised prolines (a), synthesised F-Hyp diastereomers (b), F-Hyp containing derivatives of VH032 (c) and cocrystal structure of a F-Hyp containing HIF-1α 19-mer peptide bound to VCB (PDB 6GFX) (d).^86^

Hydroxylation of proline in 4-position is known to bias the conformational preference of the pyrrolidine ring from a C^4^-*endo* pucker in unmodified proline to a C^4^-*exo* pucker for Hyp (Fig. 11a).^81–83^ The C^4^-*exo* conformation benefits from a gauche effect allowing for n→π* interaction between the N-terminal Hyp carbonyl oxygen lone-pair and the C-terminal Hyp C=O π* orbital.^84,85^ This interaction stabilises the *trans* configuration of the amide bond in Hyp-containing peptides and is an important structural requirement for binding of the Hyp residue to VHL.^86^ Similar effects on conformational preference have been observed for fluorinated prolines.^81,87–89^

Aiming at unravelling the consequences of simultaneous hydroxylation and fluorination of proline on its conformational preference, the Ciulli laboratory studied all four possible *N*-Boc protected 3-fluoro-4-hydroxyproline isomers, so called ‘F-Hyp's (Fig. 11b).^86^ Computational analysis of *N*-acetyl F-Hyp methyl esters revealed that a *cis*-arrangement of fluorine and carbonyl group lead to a 1 : 1 mixture of C^4^-*exo* and C^4^-*endo* pucker, while a *trans*-arrangement favoured the C^4^-*endo* conformation. 3-Fluorination of 4-hydroxyproline led to marginal increased hydrogen bond acidity of the hydroxy group regardless of stereochemistry, and only modestly improved hydrogen donor capacity of the hydroxy group in F-Hyps. To investigate the influence of Hyp fluorination on binding to the Hyp site of VHL, F-Hyp-containing 19-mer peptides of HIF-1α as well as F-Hyp containing VH032-derivatives 33 and 34 (Fig. 11c) were synthesised and biophysically and structurally characterised.^86^

Both (3*R*,4*S*)-F-Hyp peptide and inhibitor derivatives showed binding affinities to VHL comparable to the parent Hyp-containing compounds, while the (3*S*,4*S*)-F-Hyp derivatives featured considerably lower binding affinities. According to the cocrystal structure of the (3*R*,4*S*)-F-Hyp-containing peptide bound to VCB, the binding mode of the native peptide is preserved (Fig. 11d), and additional beneficial contributions arise from interaction of the fluorine substituent with the side chains of Trp117 and Ser111 and with the carbonyl of His110. These favourable interactions seem to compensate for the energy penalty arising from conformational pyrrolidine ring rearrangement to the C^4^-*exo* pucker required for binding to VHL. The F-Hyp containing inhibitors 33 and 34 both recapitulate the binding mode of VH032, while benefiting from additional contacts of F with Trp117, Ser111 and His110. The 12-fold higher binding affinity of 33 to VCB, compared to 34, was rationalised using quantum mechanics/molecular mechanics (QM/MM) by substantially increased electrostatic potential of the hydroxyl group of **33** compared to 34, thereby strengthening the hydrogen bond acceptor (HBA) interaction with Ser111. Inhibitor 33 benefits from slightly improved pharmacological properties compared to VH032, featuring a higher microsomal stability and higher membrane permeability, but with 10-fold lower membrane permeability compared to VH298.^62^ In accordance with the biophysical and pharmacokinetic characterisation of the VHL binders 33 and 34, their incorporation in the reported VHL-recruiting PROTACs showed more potent degrader activity with PROTACs containing the (3*R*,4*S*)-F-Hyp isomer. Despite the marked loss of binary binding affinity to VHL, the MZ1-like molecular-matched pair PROTAC containing the weak-affinity (3*S*,4*S*)-epimer of F-Hyp still induced highly selective degradation of bromodomain containing protein 4 (Brd4), and with half-degrading concentration (DC_50_) values significantly lower than the *K*_d_ values for VHL binding. These findings highlight that targeted protein degradation by PROTACs does not linearly disappear with loss of binary *K*_d_ and show that weaker affinity binders on the ligase end can still work well to generate effective degraders when the lowered binding affinity is rescued by positive cooperativity.^86^

### 3.7. Efforts to expand the chemical space: further modifications of VH032/VH298

**Fig. 12:**
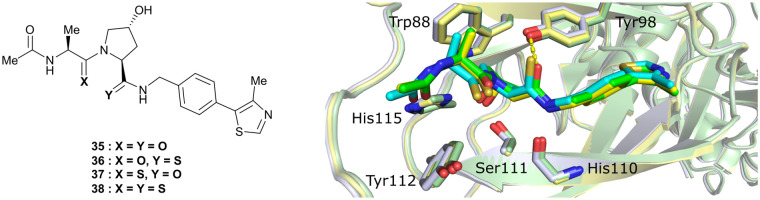
VHL inhibitors containing thioamide bioisosteres and superimposition of cocrystal structures of 35 (green), 36 (yellow) and 37 (cyan) with VCB (PDB 5NVY, 6FMJ, 6FMK).^92^

An alternative approach to structurally modify VHL inhibitors in their core region is the replacement of one or both Hyp-adjacent amide bonds by their thioamide isosteres, thereby modulating the strength of the n→π* interaction between the LHS and RHS carbonyl groups.^84,85,90,91^ As the cocrystal structure of both the native HIF-1α peptide and VH032 bound to VCB suggested a bound *trans* amide conformation allowing for n→π* interaction of the adjacent amides of Hyp, thioamide-containing analogues of VH032 were designed and analysed with regard to their conformational preference and inhibition ability.^92^ Synthesis of VHL inhibitors derived from VH032 containing a LHS, RHS or duplex thioamide incorporation was envisioned following the established synthetic route to VH032 with addition of carbonyl to thioamide conversion steps for the LHS and RHS fragments before assembling the whole inhibitor. For the LHS fragment however, carbonyl to thioamide synthetic conversion on *tert*-butyl leucine failed – presumably due to steric reasons preventing reactivity – and required resorting to a LHS alanine unit, which could be successfully converted into its thioamide analogue. Accordingly, a series of alanine-containing derivatives of VH032 featuring a RHS (36), LHS (37) or duplex (38) thioamide functionality was synthesised (Fig. 12).

Compared to the thioamide-free reference compound **35**, introduction of a LHS thioamide led to a two-fold loss in binding affinity, while exchange of the RHS carbonyl or both carbonyls led to considerably larger (10- to 40-fold) loss in binding affinity. Experimental evaluation of the solution state of the free ligands by NMR monitoring of the *cis*/*trans* isomer ratio revealed a slightly higher share of the *trans* isomer with the thioamide donor (on LHS, 37), in line with stronger n→π* donor character of thioamides compared to oxamides.^85^ Structurally, the amide to thioamide conversion induced a slight bending of the Tyr112 side chain to accommodate the LHS thioamide of 37 and 38 bound to VHL and elongated hydrogen bond distances to Tyr98 at RHS for 36 and 38 while maintaining all noncovalent interactions of reference inhibitor 35 (Fig. 12). Analysis of the interaction energy of the ligands to VHL by MM confirmed that loss in binding affinity largely stems from destabilisation of the interaction with Tyr98, highlighting the importance of the RHS carbonyl group for binding.^92^

**Fig. 13:**
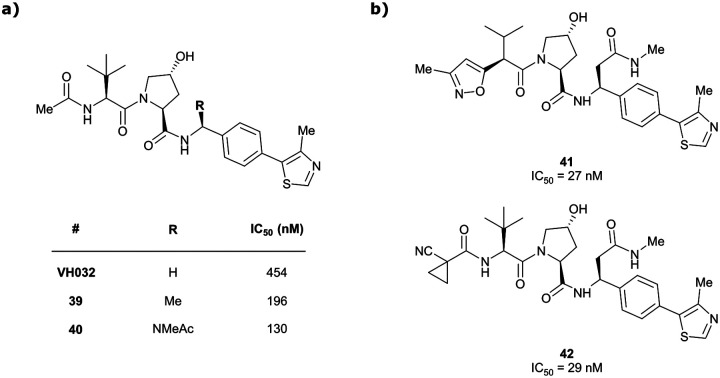
VHL inhibitors with benzylic modifications.^94,95^

Originally reported by Arvinas in the development of VHL-recruiting BET PROTAC ARV-771 (see below, Section 4.2.1. and Fig. 20),^93^ a popular modification of VH032-based VHL ligands is the stereoselective methylation of the RHS benzylic position. Subsequent attempts to further optimise the binding affinity of VHL inhibitors by the Wang laboratory assessed a series of VHL inhibitors featuring RHS benzylic modifications including methylations.^94,95^ From the set of benzylic methylated VHL binders, only the closest analogue of VH032, inhibitor 39, featured a two-fold improved binding affinity (IC_50_ = 196 nM *vs.* 454 nM for VH032 as determined by FP assay).^95^ Replacement of the methyl group with a methyl acetamide functionality further improved the binding affinity of inhibitor 40 by 1.3-fold compared to 39 (Fig. 13a).^94^

Conversely, exchange of the RHS 4-methylthiazolyl group with (pseudo)halides or alkanes of the methylated inhibitor 39 led to considerable loss in binding affinity.^95^ Further variation of the LHS part of 40 identified two novel inhibitors with considerably improved binding affinity (IC_50_ < 30 nM), featuring either 4-methylisoxazolyl (41) or cyclopropyl-carbonitrile (42, as in VH298) groups on the LHS (Fig. 13b).^94^

**Fig. 14:**
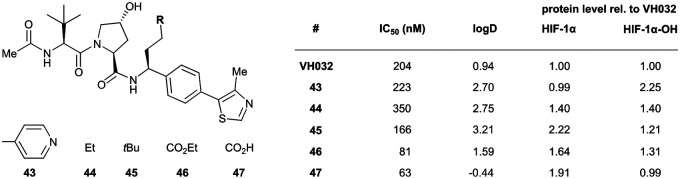
VHL inhibitors exploiting the subpocket between His110 and Tyr112.^96^

A recent ensemble approach combining virtual amino acid mutations, homology construction, native docking and MD simulations was used to analyse PPIs between HIF-1α and VHL to gain detailed insights into VHL binding pockets for the rational design of improved derivatives of VH032.^96^ Based on the identified interactions, a series of derivatives of VH032 featuring RHS benzylic modifications targeting a sub-pocket between His110 and Tyr112 was synthesised (Fig. 14). Introduction of a 4-ethylpyridyl unit (43) maintained the binding affinity of VH032 and led to a 2.25-fold increase in stabilisation of HIF-1α-OH, while an *n*-butyl residue (44) reduced binding affinity, though still slightly increasing stabilisation of HIF-1α and HIF-1α-OH through considerably improved cell permeability.^96^ Increasing the aliphatic residue to 3,3-dimethylbutyl (45) increased binding affinity, improved the cell permeability and led to a two-fold greater stabilisation of HIF-1α relative to that of VH032. Compared to 44, the introduction of an ester group (46) which could form extra hydrogen bond contacts with VHL markedly improved the binding affinity (IC_50_ = 81 nM) and improved both permeability and cellular potency as compared to VH032. Hydrolysis of this ester to the corresponding carboxylic acid (47), which as anionic carboxylate forms a salt bridge with the imidazole of His110, slightly increased the binding affinity (IC_50_ = 63 nM) but led to a significant drop in cell permeability, overall leading to loss in cellular potency. Like VH032, inhibitors 43, 46 and 47 promoted HIF-1α and HIF-1α-OH protein levels and featured no cell toxicity, thus could qualify as VHL/HIF-1α interaction disruptors.

Although not qualified as chemical probe VHL inhibitors, as the most commonly used VH298, the herein described compounds bearing further modifications, foremost the derivatisations in the RHS benzylic position, constitute highly valuable additions to the library of known VHL inhibitors. This is especially valuable in PROTAC design, where fine modulation of both the degrader's binding affinity and cellular permeability can be achieved by introducing small structural modifications to the VHL binder's molecular scaffold.

### 3.8. Efforts with virtual screening

**Fig. 15:**
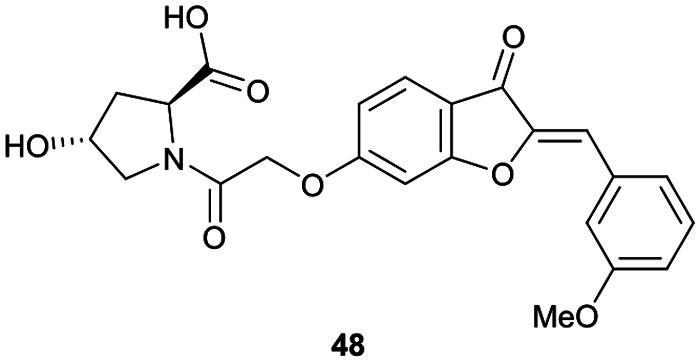
Alternative VHL inhibitor derived from virtual screening approach.^97^

Virtual screening approaches have been used to identify and develop novel VHL binders. An early structure-based virtual screening of a library of 90.000 natural products and natural product-like molecules *via* docking against VHL led to identification of binder 48 (Fig. 15), which has an IC_50_ value of 2.3 μM that compared well to that of the best first-generation inhibitors known at the time, *e.g.* compound 12.^97^ According to molecular modelling, 48 occupies the Hyp binding site of VHL, but with the Hyp fragment of 48 interacting with Ser68 in the LHS pocket instead of interacting with Ser111 as the previously reported first-generation VHL binders. Although 48 led to increased gene expression of downstream targets of HIF-1α and promoted angiogenesis in *in vivo* zebrafish models, no further development of VHL binders based on 48 has been disclosed since.^97^

Using an ensemble-based virtual screening approach, VH032, VH101, VH298 and three other related VHL inhibitors have been used as training set for pharmacophore modelling, eventually leading to the identification of a set of potential alternative binders for the Hyp site of VHL, but these have yet to be experimentally validated.^98^

Although conceptually promising for the identification of novel lead structures for inhibitor design, virtual screening-based VHL binder development has not yet delivered *bona-fide* VHL ligands and further in-depth experimental validation will be necessary to confirm the suitability of hits generated using this method.

Exploiting Hyp as key binding motif, high-affinity ligands of VHL with double-digit nanomolar binary *K*_d_ values have been developed using structure-based rational design, with VH032, VH101 and VH298 as most prominent and most widely utilised representatives. Though these binders already qualify as chemical probes, many opportunities remain for further ligand optimisation with improved binding affinities, especially in so far “underdeveloped” sites such as the RHS phenyl moiety. Ligand optimisation beyond the aforementioned scaffolds presents an ongoing quest and seems highly promising especially for application as ligands in PROTACs, where subtle modifications might enable interaction with the respective POI or open alternative exit vectors and chemistries for linker attachment.

## 4. VHL ligands for PROTACs

**Fig. 16:**
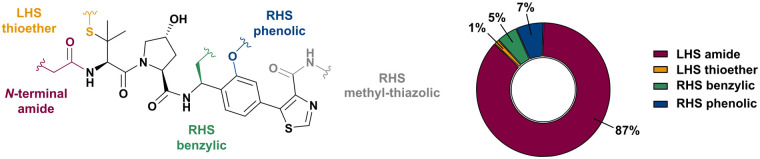
Exit vectors for linker attachment used in VHL-recruiting PROTAC design.‡

Shortly after the report of the first VHL 2^nd^ generation inhibitor featuring cellular activity, VH032, several studies proved successful at introducing this small-molecule VHL ligand as E3 ligase recruiting moiety in PROTACs.^99–101^ Along with the development of VHL-recruiting PROTACs, several suitable exit vectors at the VHL ligand scaffold have been identified and exploited for linker attachment (Fig. 16). Building on the peptidic structure of the LHS of the 2^nd^ generation VHL inhibitors, linkers can be readily attached to the N-terminus of VH032*via* an amide bond (Fig. 16, red) by exchanging the terminal acetyl group with a suitable linker. This growth vector mimics the orientation of the LHS peptide chain of the native VHL substrate HIF-1α-OH, thus avoids clashing with the protein surface. With 87% among all tethering vectors, N-terminal linker attachment *via* amide bond is by far the most commonly used conjugation vector for VHL-recruiting PROTACs.

Alternatively, the side chain of the key *tert*-Leu group at the LHS, which is conveniently pointing away from the protein surface, can serve as an alternative LHS exit vector. For this purpose, exchange of the *tert*-Leu group of the VHL binder with penicillamine has been reported,^102^ allowing linker attachments through a thioether functionality (Fig. 16, yellow).

On the inhibitor's RHS, further solvent-exposed atoms have been derived as potential exit vectors for linker attachment from analysis of the cocrystal structure of VH032 with VCB (see Fig. 7b). Benefiting from the improved binding potency of benzylic methylated VHL inhibitors (see Section 3.7), this solvent-exposed benzylic methyl group has been exploited as a tethering vector (Fig. 16, green). Analogous to the benzylic modification of inhibitors 40, 41 and 42, linker attachment at this benzylic position has been achieved *via* an amide bond and introduced in 5% of VHL-recruiting PROTACs to date.^94^ Very recently, installation of alkyl linkers at the benzylic position has been reported, requiring early introduction of the linker functionality as part of the VHL ligand synthesis.^103^

Adjacent to the benzylic position, the *ortho*-position of the first RHS arene is solvent-exposed as well and has been exploited as exit vector in 8% of VHL-recruiting PROTACs to date (Fig. 16, blue). To allow modular attachment of linkers, *ortho*-phenolic derivatives of the VHL inhibitors have been synthesised starting from a 4-bromo-2-hydroxybenzonitrile building block.^100^ Lastly, derivatisation of the solvent-exposed methyl group of the 4-methyl-thiazole fragment to a carboxylic acid has been proposed as further exit vector (Fig. 16, grey).^104^

More than 750 VHL-recruiting PROTACs have been published to date,‡‡Statistical overview generated using data extracted from PROTAC-DB^218^ (https://cadd.zju.edu.cn/protacdb/, as of 28th January 2022). targeting more than 50 different POIs for proteasomal degradation. A comprehensive overview of well-characterised VHL-recruiting PROTACs summarising targets, exploited exit vectors and VHL ligands, and key biophysical, structural, and cellular potency data is provided in Table 1. The following sections will focus on structure-guided design of VHL-recruiting PROTACs featuring different linker exit vectors and highlight VHL-recruiting PROTACs endorsed as chemical probes. More information on therapeutic targets of VHL-recruiting PROTACs^105^ and synthetic strategies to various VHL-based PROTACs^23^ can be found in comprehensive reviews elsewhere.

**Table 1:** Summary of well-characterised VHL-recruiting PROTAC degraders sorted by targets featuring their constituting VHL ligands, ternary binding affinity and cooperativity, information about ternary crystal structure availability as well as cellular activity data

Target	VHL ligand and exit vector	PROTAC name	PROTAC	*K*_d_ [nM]	*α*	Crystal structure (PDB code)	DC_50_ [nM]	*D*_max_ [%]	EC_50_ [nM]	Ref.
Brd2, Brd3, Brd4	VH032	MZ1	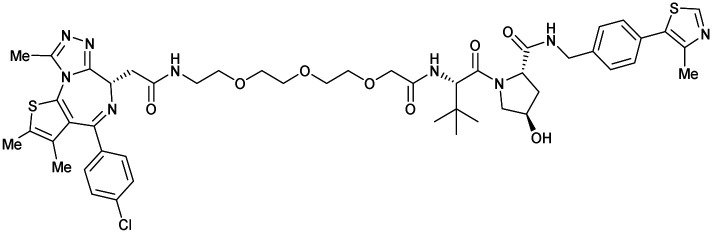	0.9^106^^,^^a^ (Brd2^BD2^)	32^106^^,^^a^ (Brd2^BD2^)	Yes (5T35)	40^f^ (Brd2)	98^f^ (Brd2)	27^l^	101,102,106,107
	N-terminal			8^106^^,^^a^ (Brd3^BD2^)	3.6^106^^,^^a^ (Brd3^BD2^)		100^f^ (Brd3)	100^f^ (Brd3)		
				1^106^^,^^a^ (Brd4^BD2^)	22^106^^,^^a^ (Brd4^BD2^)		2.5^f^ (Brd4)	100^f^ (Brd4)		

Brd2, Brd3, Brd4	Benzylic methylated VH032	ARV-771	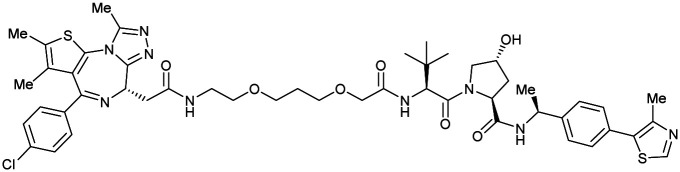	2.5^108^^,^^b^ (Brd4^BD2^)	13^108^^,^^b^ (Brd4^BD2^)	n.d.	>5^g^ (Brd2, Brd3, Brd4)	>99^h^	90^109^^,^^g^	93,108,109
	N-terminal									

Brd2, Brd3, Brd4	VH032	AT1	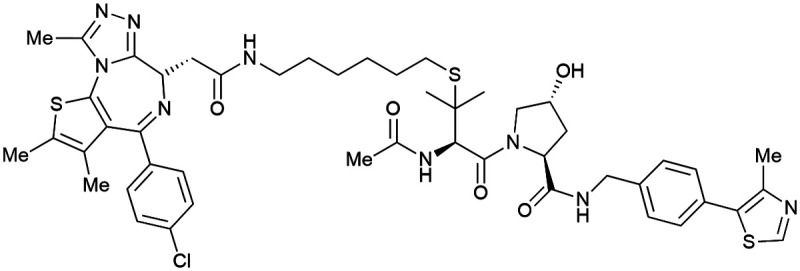	24^106^^,^^a^ (Brd4^BD2^)	4.7^106^^,^^a^ (Brd4^BD2^)	n.d.	>90^102^^,^^f^ (Brd4)	n.d.	n.d.	102,106
	LHS thioether									

Brd2, Brd3, Brd4	VH032	macroPROTAC-1	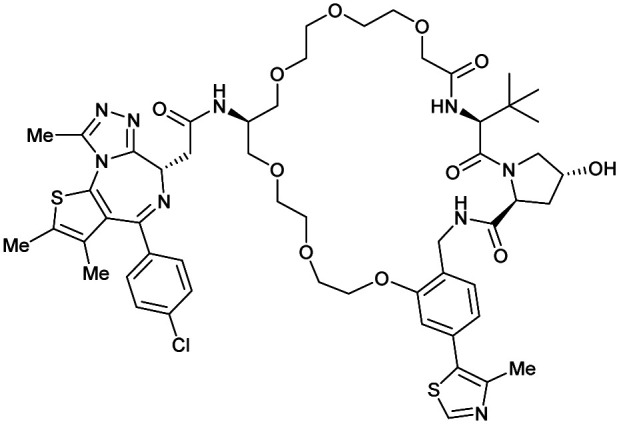	2^c^ (Brd4^BD2^)	20^c^ (Brd4^BD2^)	Yes (6SIS)	25^f^ (Brd4)	92^f^ (Brd2)	300^l^	110
	Cyclic N-terminal & phenolic							20^f^ (Brd3)		
								99^f^ (Brd4)		

Brd2, Brd3, Brd4	VH032	SIM1	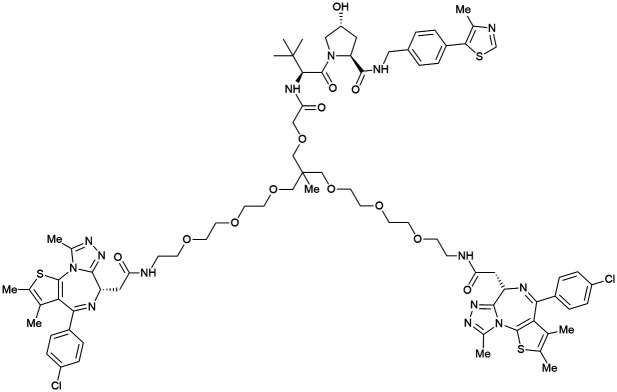	45^a^ (Brd2^BD1-BD2^)	13.8^a^ (Brd2^BD1-BD2^)	n.d.	1.1^i^ (Brd2)	n.d.	2^g^	109
				98^a^ (Brd4^BD1-BD2^)	6.4^a^ (Brd4^BD1-BD2^)		3.3^i^ (Brd3)			
	N-terminal						0.7^i^ (Brd4)			

Brd4	VH032	**Compound**9	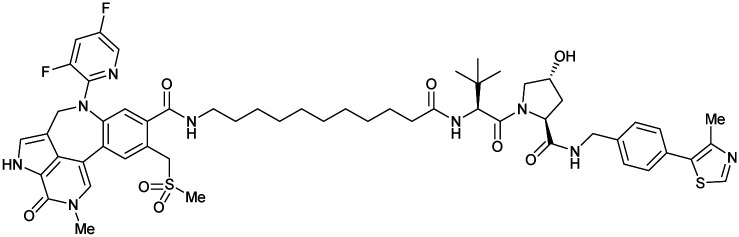	n.d.	n.d.	Yes (7KHH)	0.095^j^	96^j^	0.63^j^	111
	N-terminal									

Brd7, Brd9	VH101	VZ185	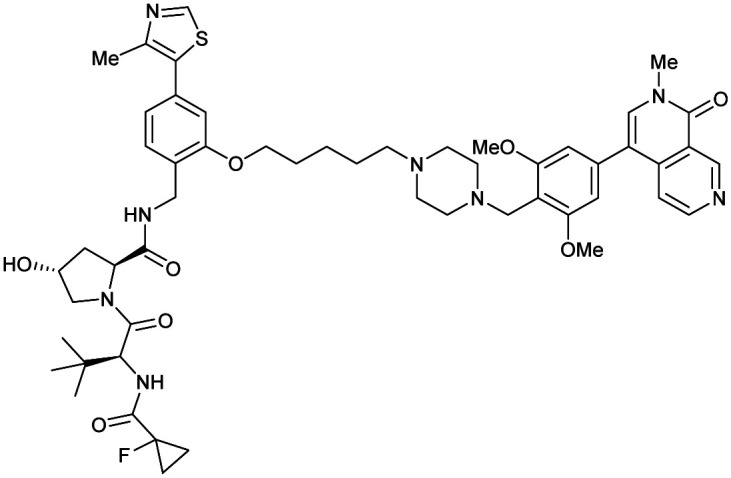	27^c^ (Brd9)	0.96^c^ (Brd9)	n.d.	1.8^k^ (Brd7)	>95^k^	3^u^	112
	RHS phenolic						4.5^k^ (Brd9)			

SMARCA2, SMARCA4, PRBM1	VH101	ACBI1	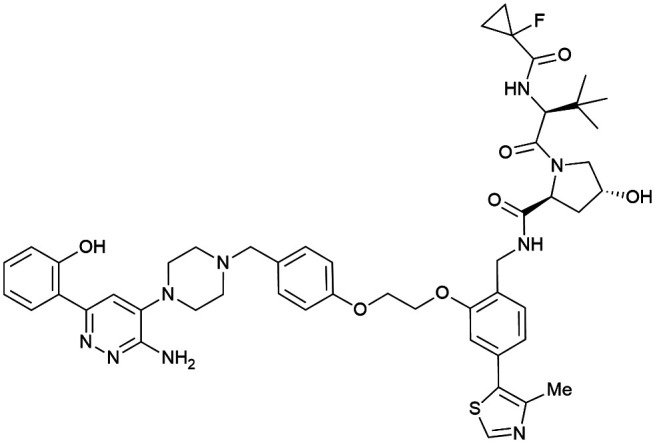	16^b^ (SMARCA2^B^^d^)	30^d^ (SMARCA2^B^^d^)	n.d.	6^l^ (SMARCA2)	>99^l^	28^l^	113
	RHS phenolic				11^d^ (SMARCA4^B^^d^)		11^l^ (SMARCA4)			
					12 d (PRBM1)		32^l^ (PRBM1)			

VHL	VH032	CM11	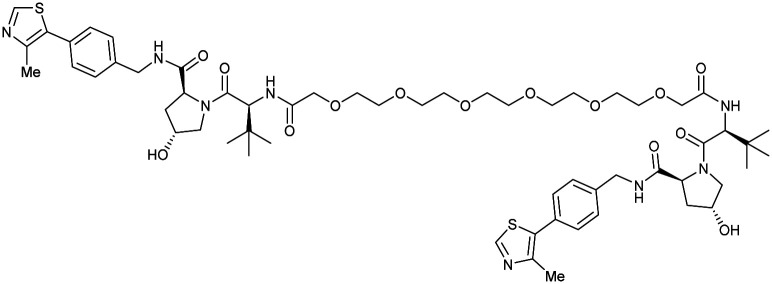	11^c^ (pVHL19)	18^c^ (pVHL19)	n.d.	n.d.	9^f^ (pVHL19)	n.d.	114
	N-terminal			25^c^ (pVL30	7.5^c^ (pVHL30)			>99^f^ (pVHL30)		

CRBN	VH032	**PROTAC 14a**	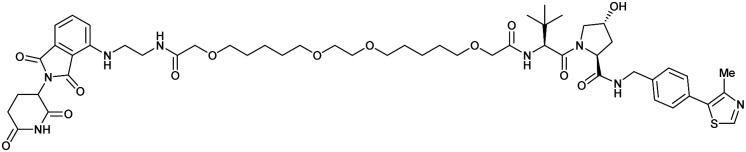	n.d.	n.d.	n.d.	200^f^	88^f^	n.d.	115
	N-terminal						200^i^	98^i^		

CRBN	VH032	CRBN-6**-5-5-VHL**	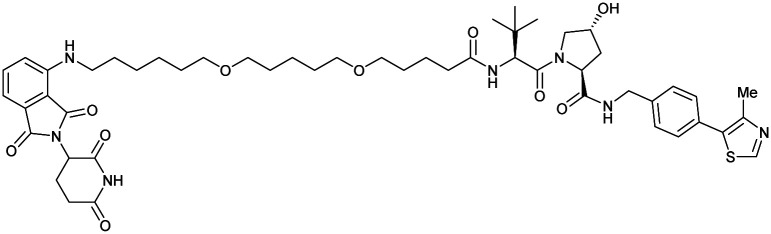	n.d.	n.d.	n.d.	1.5^m^	90^m^	n.d.	116
	N-terminal									

PTK2	VH032	BI-0319	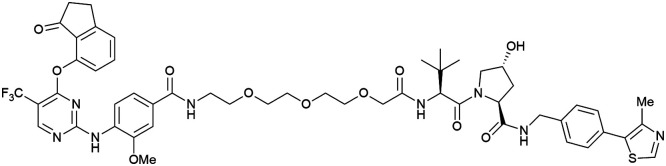	n.d.	n.d.	n.d.	243^n^	80^n^	5012^w^	117
	N-terminal									

PTK2	**Benzylic methylated**	GSK215	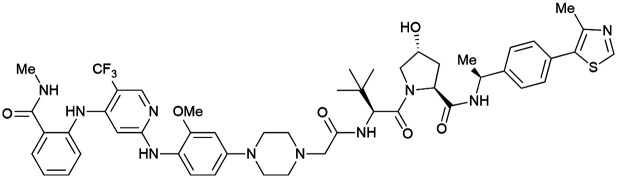	n.d.	n.d.	Yes (7PI4)	1.3^n^	99^n^	n.d.	118
	VH032									
	N-terminal									

SGK3	VH032	SGK-PROTAC1	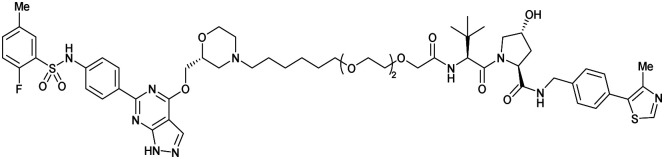	n.d.	n.d.	n.d.	<100^i^	80^i^	n.d.	119
	N-terminal									

AR	VH032	ARCC-4	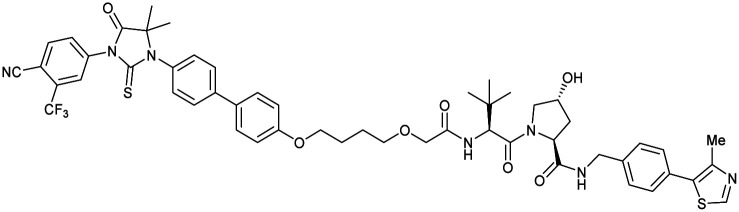	n.d.	n.d.	n.d.	5^h^	95^h^	280^h^	120
	N-terminal									

TBK1	VH032	**PROTAC 3i**	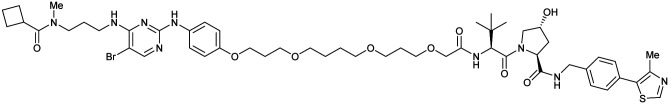	n.d.	n.d.	n.d.	12	96	n.d.	121
	N-terminal									

EGFR	VH032	Gefitinib-based PROTAC 3	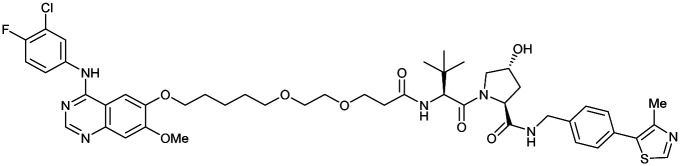	n.d.	n.d.	n.d.	11.7^o^	99^o^	n.d.	122
	N-terminal									

Bcl-xL	VH032	PROTAC-6	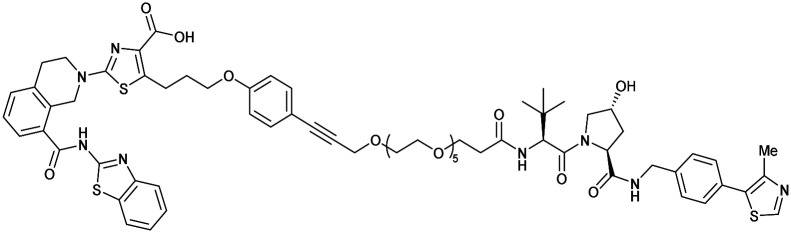	290^a^	0.72^a^	Yes (6ZHC)	4.8^p^	76^p^	578^v^	123
	N-terminal									

WDR5	VH032	MS33	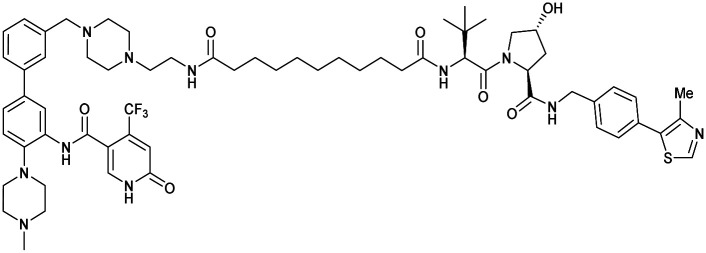	520^c^	1.66^c^	Yes (7JTO)	260^l^	77^l^	n.d.	124
	N-terminal									

WDR5	MS67		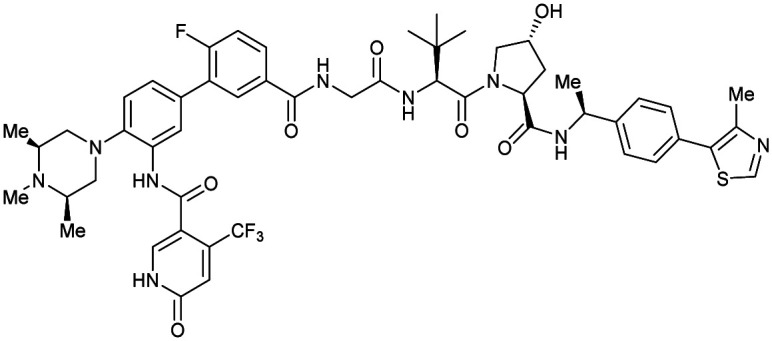	52^c^	2.74^c^	Yes (7JTP)	3.7^l^	94^l^	15^l^	124
		**Benzylic methylated** VH032								
		N-terminal								

WDR5	VH032	Homer	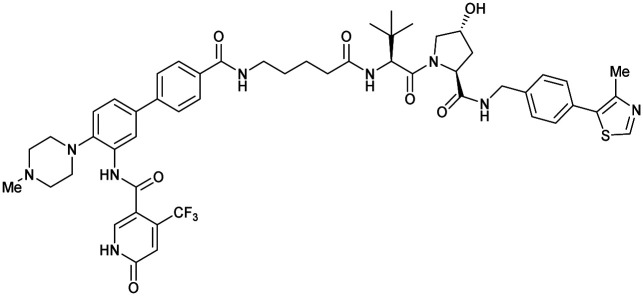	n.d.	n.d.	Yes (7Q2J)	53^l^	58^l^	n.d.	125
	N-terminal									

LRRK2	VH101	XL01126	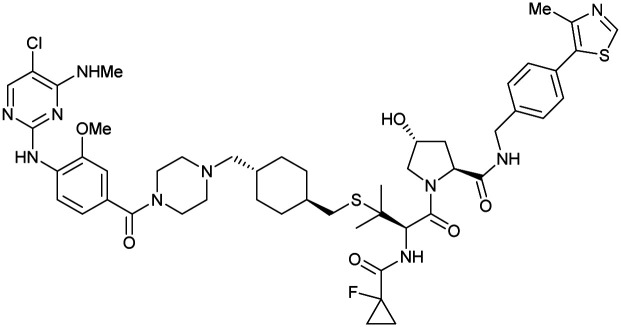	n.d.	5.7^e^	n.d.	32 (LRRK2)^q^	82 (LRRK2)^q^	54^q^	126
	LHS thioether						14 (LRRK2 ^G2019^^s^)^r^	90 (LRRK2 ^G2019^^s^)^r^	15^r^ (Rab10 dephosphorylation)	

*Halo-tagged proteins*	VH298	HaloPROTAC-E	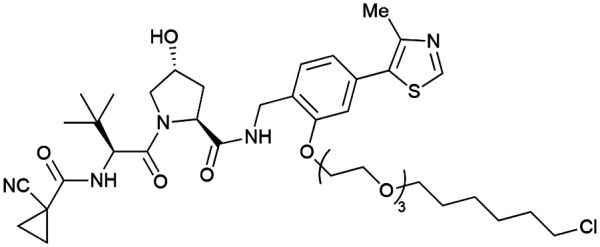	n.d.	n.d.	n.d.	3–10^s^ (SGK-Halo)	95^s^ (SGK-Halo)	No cytotoxicity up to 1 μM^i^^s^	127
	RHS phenolic						3–10^s^ (Halo-VPS34)	95^s^ (Halo-VPS34)		

*Bromo-tag-Brd2*	VH032	AGB1	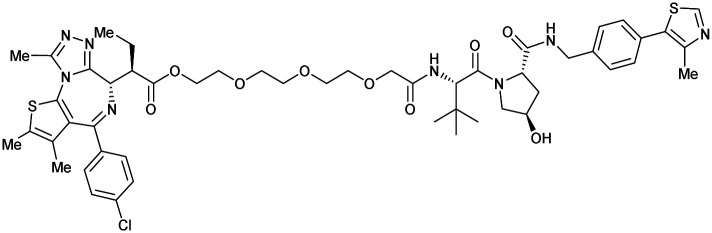	11^b^	11.1^b^	n.d.	<15^t^	92^t^	No cytotoxicity up to 1 μM^g^^i^^l^	128
	N-terminal									

*FKBP12* ^*F36*^ v *fusion proteins*		dTAG^V^-1	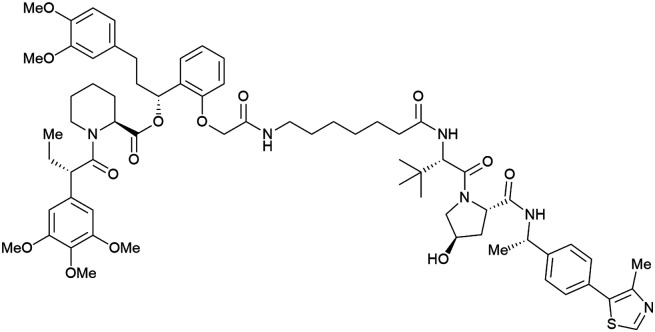	n.d.	n.d.	n.d.	n.d.	>99^x^	n.d.	129
	**Benzylic methylated** VH032									
	N-terminal									

a *K*_d_ and cooperativity α values of ternary complex formation, determined by SPR.

b *K*_d_ and cooperativity α values of ternary complex formation, determined by FP.

c *K*_d_ and cooperativity α values of ternary complex formation, determined by ITC.

d Cooperativity α values of ternary complex formation, determined by TR-FRET.

e Cooperativity α value of ternary complex formation derived from *K*_*i*_ values, determined by nanoBRET.

f In HeLa cells.

g In 22Rv1 cells.

h In VCaP cells.

i In HEK293 cells.

j In PC3-S1 cells.

k In RI-1 cells.

l In MV4;11 cells.

m In MM1S cells.

n In A549 cells.

o In HCC827 (exon 19 del) cells.

p In THP-1 cells.

q In MEF cells.

r In LRRK2^G2019S^ MEF cells.

s In HEK293-SGK-Halo or HEK293-Halo-VPS34 cells.

t In BromoTag-Brd2 HEK293 cells.

u In EOL-1 cells.

v In MOLT-4 cells.

w In SNU-387 cells.

x In EWS/FLI^−/−^ cells.

### 4.1. Pioneering first VHL-recruiting PROTACs

**Fig. 17:**
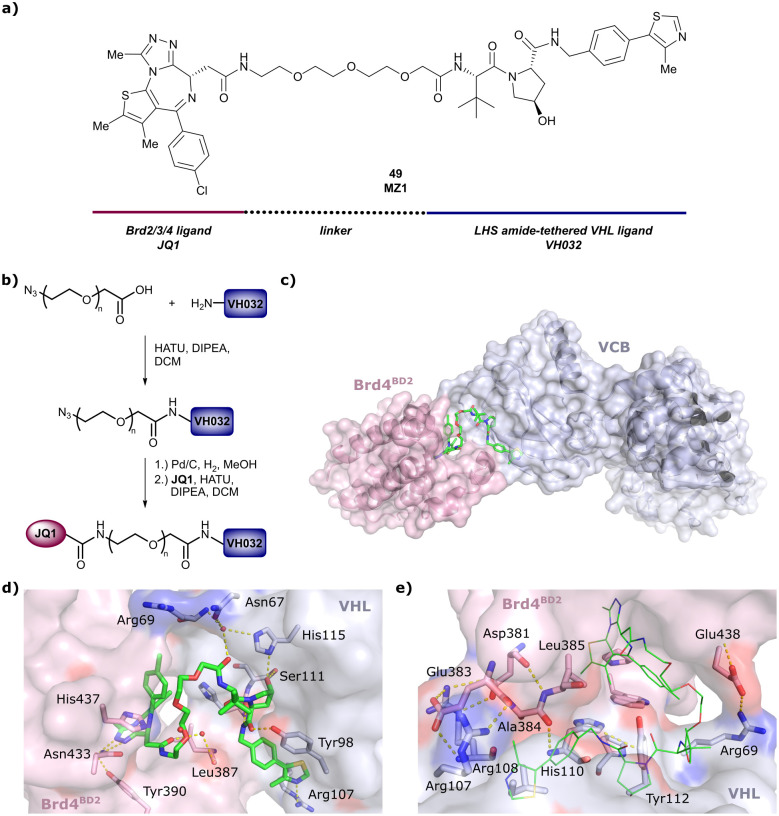
Development of first VHL targeting PROTAC degrader. Structure of MZ1 (49) (a), synthetic route to access such degraders (b), and ternary cocrystal structure of MZ1 bound to VCB and Brd4^BD2^ (c), PROTAC–protein interactions (d) and *de novo* PPI between Brd4^BD2^ and VHL (e) (PDB 5T35).^102^

One of the first applications of small-molecule inhibitors as VHL-recruiting moieties in degrader technology was reported in mid-2015 by the Ciulli laboratory integrating either VH032 or ligand 16 in a first set of Bromo- and Extra-Terminal (BET) proteins targeting PROTACs. These BET proteins targeting PROTACs were assembled from VH032 or 16 as VHL recruiter, polyethylene glycol (PEG) linkers featuring a carboxylic acid on one side and an azide group on the other, and pan-BET selective bromodomain inhibitor JQ1^130^ as BET ligand.^101^ In a two-step synthetic strategy, three PROTACs were formed by initial HATU-mediated amide coupling of the N-terminal free amine of VH032 or 16 with the carboxylic acid of the linker followed by reduction of the azide to the free amine and another amide bond formation with the hydrolysed carboxylic acid of the BET ligand JQ1 (Fig. 17b).

Initial assessment of these PROTACs established their cellular activity and selectivity in preferentially inducing degradation of Brd4 over Brd3 and Brd2 in several cell lines. From this small library of PROTACs, degrader 49 (MZ1) turned out to be the most efficient and potent degrader (Fig. 17a). The observed depletion of Brd4/3/2 levels proved to be both VHL- and proteasome-dependent, in line with the expected mode of action of PROTAC degraders. Promisingly for potential therapeutic application, the protein levels of VHL and HIF-1α were not affected at the dosage of MZ1 used in the degradation studies, thus avoiding side effects from potential VHL inhibition.^101^ In a later follow-up detailed structural analysis, the ternary crystal structure of MZ1 bound to VCB and Brd4^BD2^, which represents the first reported ternary crystal structure of a complex of POI/PROTAC/E3 ligase, has been resolved at 2.7 Å (Fig. 17c). This structure not only offered a first glimpse of how a PROTAC can bring together a target protein in tight complex with the E3 ligase, but also provided the basis for structure-based design of further BET protein degraders (see below, Section 4.3).^102^

In the ternary complex, MZ1 is embedded between the two proteins, inducing neo-protein-ligand contacts as well as new PPI of both hydrophobic and electrostatic nature. The hydrophobic base of the bowl-shaped interface of VCB and Brd4^BD2^ arises from PPIs between the so-called “WPF shelf” (Trp374, Pro375, Phe376) of Brd4^BD2^ with the residues of Arg69, Pro71 and Tyr112 of VHL and from interaction of Ala384 and Leu385, located in the ZA loop of Brd4^BD2^, with the hydrophobic side chains of Arg108, Ile109 and His110 of VHL. Furthermore, electrostatic interactions form the rim of the bowl-shaped interface: Glu438 from the BC loop of Brd4^BD2^ contacts Arg69 of VHL and on the opposite side of the bowl, Asp381 and Glu383 from the ZA loop of Brd4^BD2^ pair with the residues of opposite charge of Arg107 and Arg108 of VHL (Fig. 17e). MZ1 resides inside this bowl, with its constituting ligands at each end recapitulating the binding modes observed for the isolated inhibitors. Crucially, additional protein–PROTAC interactions were observed, including a hydrogen bond between the linker's oxygen close to the BET ligand end and His437, a BD2-specific residue, and van-der-Waals interactions between the BC loop of Brd4^BD2^ with the PEG linker (Fig. 17d).^102^ This ternary complex crystal structure highlighted the role of the PROTAC degrader to induce novel PPIs in addition to protein–ligand interactions and the potential of harnessing additional stabilising interactions with the linker.

**Fig. 18:**
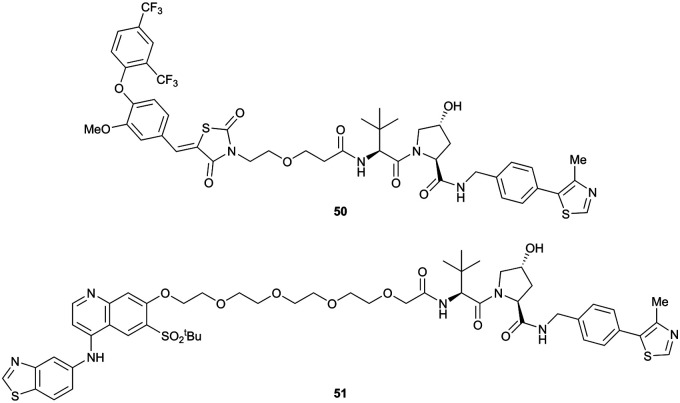
First ERRα (50) and RIPK2 (51) targeting VHL-recruiting PROTACs.^99^

Shortly after the disclosure of MZ1, the Crews laboratory in collaboration with GSK also reported their first VHL-recruiting PROTACs based on VH032. In their proof-of-concept study, PROTAC degraders targeting the oestrogen-related receptor alpha (ERRα) and the receptor-interacting serine/threonine-protein kinase 2 (RIPK2) were developed and shown to be active *in vivo* (Fig. 18).^99^

For both PROTACs, corresponding POI inhibitors were attached *via* HATU-mediated amide coupling to the N-terminus of VH032 using a PEG-based linker. Dose-dependent degradation of the target POI was observed in a proteasome-dependent manner in treatments with 50 or 51, while no noteworthy stabilisation of HIF-1α occurred up to a PROTAC concentration of 30 μM. Further confirming the essential role of the PROTAC in this degradation process, the POI levels were not affected by a treatment with the isolated inhibitors of the POI or VHL.^99^

**Fig. 19:**
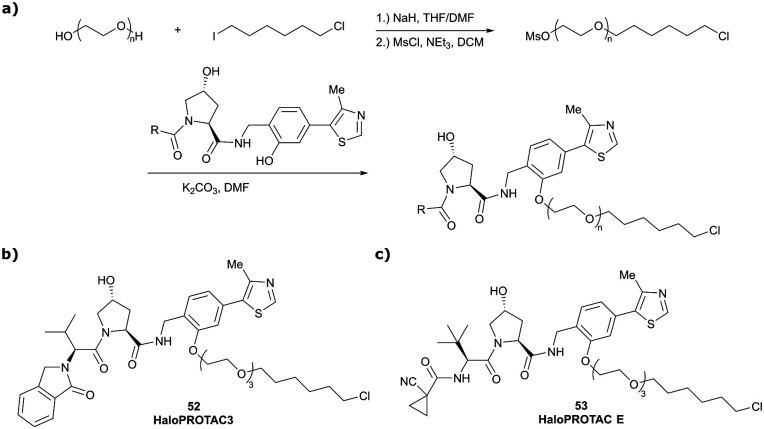
Synthetic strategy to access phenolic linked HaloPROTACs (a), and structures of HaloPROTAC3 (52) (b)^100^ and optimised HaloPROTAC-E (53) (c).^127^

In a second publication published almost simultaneously, the Crews laboratory/GSK collaboration disclosed VHL-ligand based HaloPROTACs as degraders for HaloTag7 fusion proteins.^100^ VHL inhibitors were conjugated with hexyl chloride tags, which covalently react with HaloTag units. From a HaloPROTAC library featuring varied linker lengths at either the RHS N-terminal or LHS phenolic position, the phenolic-tethered HaloPROTAC3 (52, Fig. 19b) was the most efficient degrader of exogenously-expressed green fluorescent protein (GPF)-HaloTag7, with a DC_50_ of 19 nM and a maximal degradation (*D*_max_) of 90% without affecting HIF-1α protein levels. HaloPROTACs featuring phenolic linkers were generated by base-mediated reaction of the phenol with a terminal mesylate group of the pre-formed linker-chlorohexyl tag conjugate (Fig. 19a). Efficient degradation of further cytosolic HaloTag7 fusion proteins proved the generality of the concept and the utility of HaloPROTAC3 as tool in chemical genetics studies. A few years later the Ciulli and Alessi laboratories reported optimised HaloPROTAC-E (53, Fig. 19c), by introducing the same cyano-cyclopropyl capping group as in VH298 in the VHL ligand moiety, and demonstrating potent, rapid and effective degradation of homozygously CRISPR’ed knock-in proteins at endogenous levels.^127^

### 4.2. Structure-guided rational design of N-terminal tethered VHL-recruiting PROTACs

Since these pioneering studies by the Ciulli and Crews laboratories, the vast majority of subsequent VHL-recruiting PROTACs have exploited the LHS N-terminal position as linker attachment site. Within the scope of this review, only selected examples of N-terminally tethered VHL-recruiting PROTACs endorsed as high-quality degrader probes will be discussed.

#### 4.2.1. BET protein targeting PROTACs

**Fig. 20:**
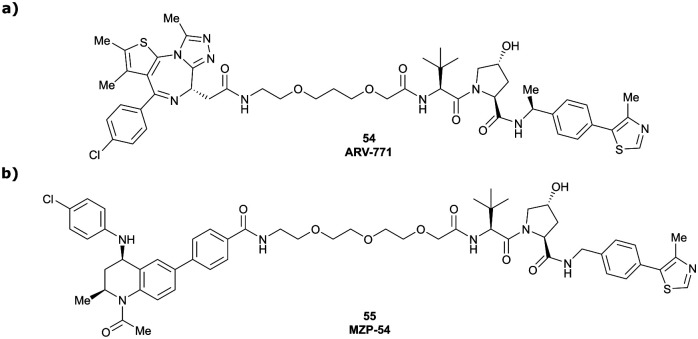
Development of further BET targeting PROTACs. Brd2/Brd3/Brd4 pan-selective degrader ARV-771 (54) (a),^93^ and Brd3/Brd4 selective degrader MZP-54 (55) (b).^107^

Following the disclosure of the BET degrader MZ1 by the Ciulli laboratory, a team at Arvinas disclosed the structurally closely related Brd2/3/4 pan-selective BET degrader ARV-771 (54, Fig. 20a).^93^ Using already established amide coupling chemistry, the slightly shorter ether-linker (8 atoms in ARV-771*vs.* 10 atoms in MZ1) was attached to a modified version of VH032 carrying an additional methyl group in the benzylic position at the RHS of Hyp. ARV-771 qualified as a potent degrader of Brd2, Brd3 and Brd4 with DC_50_ values <5 nM in 22Rv1 cancer cells and led to decreases in tumour size in xenograft models in mice.^93^ Together with MZ1, ARV-771 has been widely used as a benchmark PROTAC degrader of BET proteins (see below, Sections 4.6 and 4.7). Following up on the quest of designing potent BET degraders, the Ciulli laboratory reported an alternative series of PROTACs using the ∼10-fold higher affinity BET inhibitor I-BET726 as POI ligand instead of JQ1 used in MZ1 and PEG2,3,4 linkers.^107^ The related MZP PROTAC series featured conjugation *via* a distinct exit vector relative to that of the MZ series, based on the BET ligands binding mode. In contrast to MZ1 which preferentially degrades Brd4, PROTACs of this new series were equally effective at degrading Brd3 and Brd4. The PEG_3_-linked MZP-54 (55, Fig. 20b), the most potent degrader of this PROTAC series, induced preferential depletion of Brd3 and Brd4 over Brd2. Interestingly, MZP-54 was a less potent degrader than MZ1 and featured a narrower activity window, despite being constructed from a 10-fold more potent BET inhibitor. These effects could be rationalised by the negative cooperativity observed for the VCB/MZP-54/bromodomain ternary complexes,^107^ highlighting that improved binary binding affinity for the target protein does not necessarily translate into more potent target degradation in PROTAC development.

#### 4.2.2. TANK-binding kinase 1 (TBK1) targeting PROTACs

**Fig. 21:**
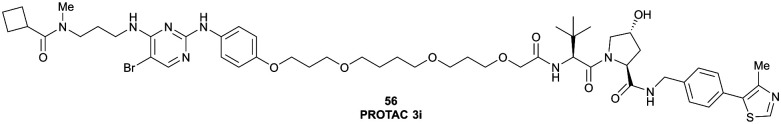
PROTAC 3i (56), chemical probe selectively degrading TBK1.^121^

TANK-binding kinase 1 (TBK1), a noncanonical member of the inhibitor of kappa B kinase (IKK) family of serine/threonine kinases, functions in cell development and the innate immune response, and has been linked to tumourigenesis as potential synthetic lethality with K-Ras.^131^ A PROTAC probe efficiently degrading TBK1 has been developed by Arvinas through SAR-guided optimisation.^121^ Starting from cocrystal structures of both ligands bound to their respective protein, the *para*-position of the pyrimidine-2-aminophenyl group of the TBK1 ligand was identified as suitable linker connection point to the LHS acetyl group of VH032, and a range of PROTACs featuring various flexible alkyl-ether linkers were synthesised and characterised. While PROTACs with linkers of <12 atoms failed to induce binding, longer linkers (up to 29 atoms) could be well accommodated and induced robust degradation of TBK1. One of the best degraders of this series, 15-atom linker containing PROTAC 3i (56, Fig. 21) with DC_50_ of 12 nM and *D*_max_ = 96%, was used as the structural basis for ligand modification of both the TBK1 and VHL binders. For variations in the 5-position of the pyrimidine in the TBK1 binder, binary binding affinity generally scaled with degradation potency – *viz.* the highest-affinity ligands with Br (PROTAC 3i), Cl or I substituents featured the lowest DC_50_ values of this series, with PROTAC 3i remaining the most potent one. On the other hand, substitution of the LHS *t*Bu group of VH032 with smaller alkyl groups reduced both binding affinity and degradation efficacy, though robust TBK1 degradation was still observed with up to 3-fold weaker binders (featuring Et or *n*Pr group instead of *t*Bu).^121^ Though the parent TBK1 inhibitor exhibited only poor selectivity for TBK1 over the structural similar IKKε protein, PROTAC 3i did not induce degradation of IKKε – adding further evidence that target degradation selectivity can be obtained beyond what would be expected from the binary target engagement alone.

#### 4.2.3. E3 ligase degrading PROTACs

Several chemical probes inducing chemical knock-down of specific E3 ligases have been developed in recent years exploiting the principle of PROTAC-mediated E3 ligase degradation.

**Fig. 22:**
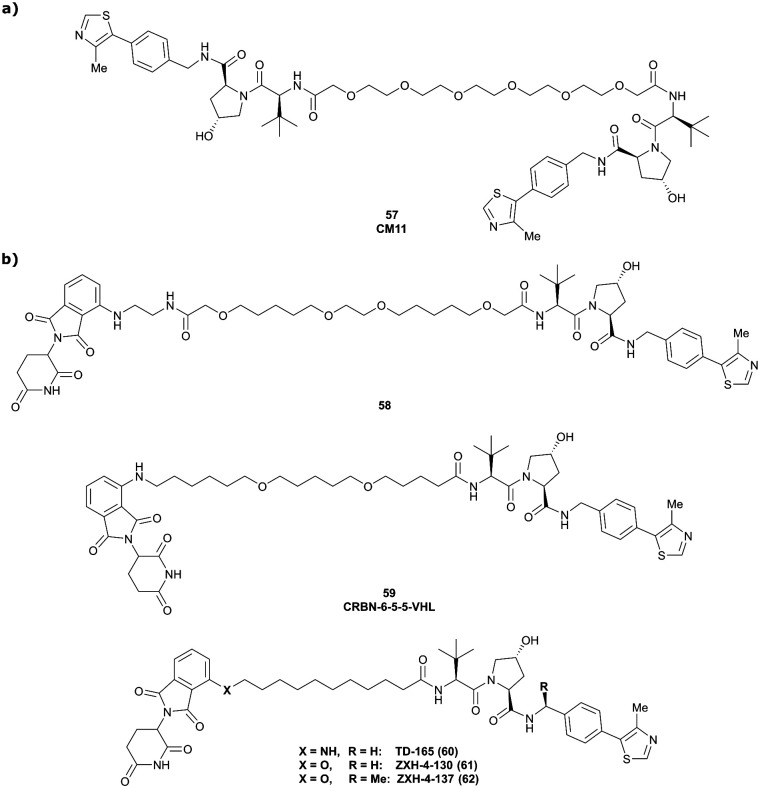
E3 ligase targeting PROTACs. Homo-PROTAC CM11 (57) inducing self-degradation of VHL (a),^114^ and CRBN-VHL Hetero-PROTACs inducing the selective degradation of CRBN (b).^115,116,132,133^

As first example, the Ciulli laboratory envisioned homo-bivalent PROTACs (Homo-PROTACs)^114^ consisting of two VHL ligands as chemical tools to induce VHL dimerisation and subsequent self-degradation by forming a VHL/Homo-PROTAC 2 : 1 ternary complex in which VHL acts both as enzyme and neosubstrate. Upon inspection of the cocrystal structure of VH032 bound to VCB (Fig. 7b), both the LHS amide functionality and the RHS phenolic position were identified as linker tethering points, and three series of Homo-PROTACs with varying PEG-linker length, symmetrical LHS amide–LHS amide, symmetrical RHS phenol–RHS phenol and asymmetrical LHS amide–RHS phenol linked HomoPROTACs were developed.^114^ The symmetric LHS amide–LHS amide PEG_5_ linked Homo-PROTAC CM11 (57, Fig. 22a) proved to be the most active degrader compared to analogues with shorter linkers and compared to members of the other series constructed *via* conjugation at the phenolic position. As shown by AlphaLISA, size exclusion chromatography and ITC, CM11 forms a stable and highly cooperative 2 : 1 complex (α = 18) with two molecules of VHL. CM11 induced full degradation of the long isoform pVHL30 with a DC_50_ < 100 nM, while marginally depleting protein levels of the short isoform pVHL19. In contrast to its parent inhibitor VH032, CM11 is only modestly stabilising hydroxy-HIF-1α in its active concentration window, thus qualifying as a chemical probe for isoform-selective knock-down of pVHL30 avoiding a HIF-dependent hypoxic response.^114^

Besides targeting E3 ligases using the self-degradation approach of Homo-PROTACs, E3 ligases can also be targeted with Hetero-PROTACs featuring ligands recruiting two different E3 ligases. In contrast to classic heterobivalent degraders, the roles of enzyme and neosubstrate are not predefined in this scenario and depend on the E3 ligase 1/PROTAC/E3 ligase 2 combination.

With regard to VHL-recruiting E3-ligase targeting PROTACs, CRBN targeting VHL-recruiting PROTACs have been independently developed by the Ciulli,^115^ Gütschow,^116^ Kim^132^ laboratories and further explored by the Gray group.^133^

Following a systematic approach, the Ciulli laboratory designed three series of CRBN-VHL PROTAC degraders covering the LHS N-terminal amide, the LHS thioether and the RHS phenolic linker tethering mode at the VHL binder, linked to pomalidomide as CRBN ligand.^115^ Several degraders from all series induced significant degradation of CRBN at 1 μM concentration, with 58 (Fig. 22b), being the most potent degrader featuring a DC_50_ = 200 nM and *D*_max_ = 75–88%, while VHL protein levels remained unaffected.

In parallel, the Gütschow laboratory optimised LHS N-terminally tethered VHL-CRBN Hetero-PROTACs regarding linker length and composition, covering short 8-atom up to long 28-atom ether-containing linkers.^116^ While PROTACs with short 8- to 14-atom linkers proved to be inefficient as degraders, various longer linkers were well tolerated, inducing selective degradation of CRBN while sparing VHL. The best degrader of this series, CRBN-6-5-5-VHL (59, Fig. 22b), qualified as a superior chemical probe compared to CRBN-Homo-PROTAC 15a previously developed by the same group,^134^ featuring a DC_50_ = 1.5 nM and inducing up 90% degradation of CRBN.

Trying to rationalise the selectivity for CRBN degradation, the Kim laboratory developed and assessed the N-terminally tethered CRBN-VHL PROTAC TD-165 (60, Fig. 22b).^132^ While concentration-dependent depletion of CRBN levels was observed, VHL levels of both short and long isoform were slightly increased. Degradation studies in cells overexpressing CRBN, VHL or both E3 ligases confirmed that relative protein levels do not bias protein degradation, as CRBN was solely degraded in all cases. Assessing several deletion mutants of CRBN revealed that the disordered region of full-length CRBN is important for efficient CRBN degradation, and that attachment of this disordered region to otherwise non-degrading CRBN deletion mutants enables their degradation.^132^

Combining proteomics and cellular degradation assays, the Gray laboratory recently evaluated two further CRBN-VHL Hetero-PROTACs, ZXH-4-130 (61) and ZXH-4-137 (62, Fig. 22b) differing from TD-165 in the attachment vector chemistry at pomalidomide, as competent degraders for CRBN knock-down.^133^ Though comparable in cellular potency, degradation of CRBN was longer lasting with ZXH-4-137 featuring an additional methyl group in the benzylic position of VH032 (complete degradation for 16 h *vs.* 4 h with ZXH-4-130). Proteomic studies identified CRBN as the sole significantly down-regulated target protein of ZXH-4-130 and ZXH-4-137. Treatment with ZXH-4-130 was able to rescue levels of proteins, such as GSPT1, that are usually targeted by CRBN for proteasomal degradation *via* small molecular-glue CRBN binders, qualifying degraders 61 and 62 as alternative chemical probes for CRBN knock-down.

#### 4.2.4. B-Cell lymphoma 2 (Bcl-2) targeting PROTACs

**Fig. 23:**
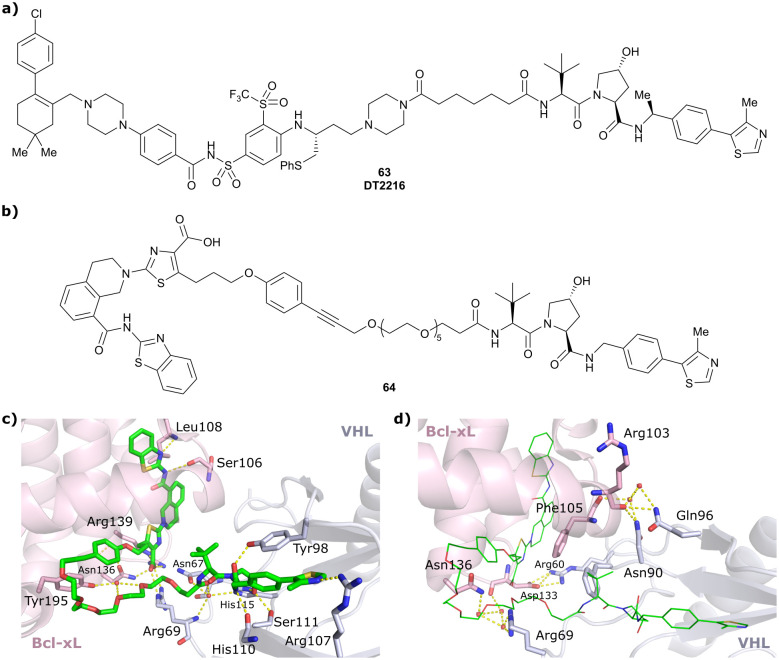
Bcl-xL targeting PROTACs DT2216 (63)^144^ (a) and 64 (b), and ternary cocrystal structure of 64 bound to Bcl-xL and VCB (PDB 6ZHC) highlighting PROTAC–protein interactions (c) and *de novo* PPIs (d).^123^

Bcl-2 and B-cell lymphoma extra-large (Bcl-xL) are well validated anti-apoptotic proteins and cancer drug targets, particularly in haematological malignancies.^135–137^ Though several potent Bcl-2 inhibitors have been developed as potential anticancer drug candidates,^138,139^ their therapeutic utility is limited, as undesired on-target inhibition of Bcl-xL in blood platelets induces rapid platelet death resulting in thrombocytopenia.^140–142^ Aiming to reduce these undesirable side effects, the Zheng and Zou laboratories integrated the potent, but cytotoxic Bcl-2 and Bcl-xL dual inhibitor ABT-263^143^ into the VHL-recruiting degrader DT2216 (63, Fig. 23a).^144^DT2216 induced selective degradation of Bcl-xL proteins in several cancer cell lines and featured an increased cellular potency. Due to low expression levels of VHL in platelets, on-target toxicity of DT2216 was considerably reduced compared to its parent inhibitor, thus rescuing its therapeutic potential, and recently enabled advancement of DT2216 as first VHL-recruiting PROTAC into phase I clinical trials.^144,145^ Exploiting a different exit vector for linker attachment on ABT-263 furthermore enabled the development of Bcl-xL and Bcl-2 dual degraders showing improved antitumour activity in leukaemia cells which are depending both on Bcl-xL and Bcl2 for survival.^146^

Building on this first proof-of-principle study on Bcl-xL targeting degraders, researchers from GSK developed and characterised a Bcl-xL-selective PROTAC 64 (Fig. 23b) derived from Bcl-xL antagonist A-1155463.^123^ Its solvent-exposed propagylic amine was derived as suitable linker vector from its binary cocrystal structure with Bcl-xL and connected *via* a LHS N-terminally amide bond to VH032 using a PEG_5_ linker. Similar binary binding of 64 to Bcl-xL compared to the parent inhibitor (*K*_d_ = 0.6 nM *vs.* 0.5 nM for the inhibitor in fluorescence displacement assay) confirmed the suitability of the linker exit vector. Though the ternary complex featured modest cooperativity (*α* = 0.72), 64 induced concentration-dependent Bcl-xL degradation with a DC_50_ value of 4.8 nM and *D*_max_ = 76%, comparable to the performance of DT2216. According to the ternary cocrystal structure of Bcl-xL/64/VCB that was solved at 1.9 Å resolution (Fig. 23c), the ligands of 64 recapitulate the binding mode of their parent inhibitors, while its long PEG linker collapsed bringing VHL close to the α2-loop-α3 region of Bcl-xL. The conformational change in 64 to allow for this binding mode is expected to require a considerable energy penalty, consistent with the modest cooperativity observed. Binding of 64 in the ternary complex induced both neo-PPIs, comprising a bidentate salt bridge between Asp133 (Bcl-xL) and Arg60 (VHL), hydrogen bonding between the main-chain carbonyl group of Arg 103 (Bcl-xL) and Asn90 and Gln96 of VHL, as well as favourable PROTAC–protein interactions (Fig. 23d). Modelling of ternary binding with Bcl-2 instead of Bcl-xL revealed that the specific binding mode of Bcl-xL/64/VCB would be unfavourable with Bcl-2, rationalising the target-selectivity of 64.

#### 4.2.5. Focal adhesion kinase (FAK) targeting PROTACs

FAK has multiple roles acting as regulator of intracellular signal transduction, driver of cancer cell growth and acting as kinase-independent scaffold for various signalling proteins.^147^ As high FAK protein levels have been detected in several solid tumour types,^148^ FAK has been addressed as a therapeutic target with inhibitors^149,150^ and both VHL- and CRBN-recruiting PROTACs.^117,118,151,152^

**Fig. 24:**
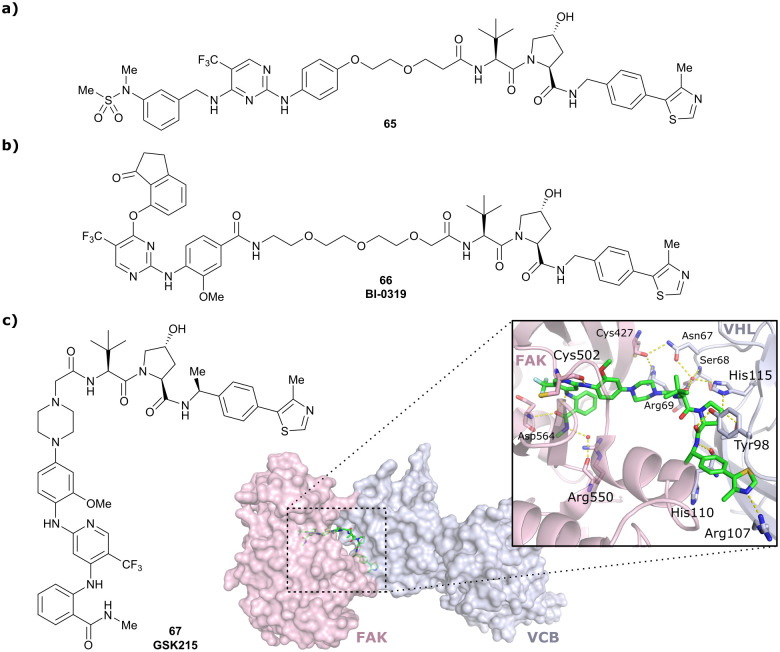
First FAK targeting PROTAC 65 (a),^151^ degrader BI-0319 (66) featuring improved selectivity (b),^117^ and structure and ternary cocrystal structure of GSK215 (67) bound to FAK and VCB (PDB 7PI4) (c).^118^

Starting from a similarly potent derivative of the clinical candidate defactinib as FAK ligand, the Crews laboratory developed N-terminally linked VHL-recruiting PROTACs differing in linker length and composition.^151^ The most efficient FAK degrader, 65 (Fig. 24a), induced up to 99% degradation of FAK, with DC_50_ = 3.0 nM, and outperformed defactinib with respect to inhibition of downstream signalling and kinase selectivity. Based on an ATP competitive inhibitor as FAK ligand, alternative CRBN- and VHL-recruiting FAK degraders were disclosed by Boehringer Ingelheim.^117^ Degrader BI-0319 (66) (Fig. 24b), the best degrader of the VHL-series, as well as the best CRBN-recruiting PROTAC qualified as potent FAK degraders in 12 liver and lung cancer cell lines and exhibited markedly improved kinase selectivity.

More recently, researchers from GSK developed FAK targeting PROTACs based on the clinical FAK inhibitor VS-4718 (originally PND-1186^153^) linked *via* N-terminally amide bond to the benzylic methylated derivative of VH032.^118^ An initial screen of a set of PROTACs with different linkers revealed correlation of DC_50_ values with ternary complex cooperativity, identifying GSK215 (67) featuring an exceptionally short amide linker as the most potent degrader with DC_50_ = 1.5 nM, *D*_max_ = 99% and *α* = 104. The ternary cocrystal structure of GSK215 bound to FAK and VCB, resolved at 2.2 Å (Fig. 24c), revealed a multitude of neo-PROTAC–protein interactions and neo-PPIs rationalising the high cooperativity of GSK215. The *in vivo* applicability of GSK215 was assessed by subcutaneous injection in mice, causing rapid and long-lasting degradation of FAK in the liver.

#### 4.2.6. WD40 repeat-containing protein 5 (WDR5) targeting PROTACs

**Fig. 25:**
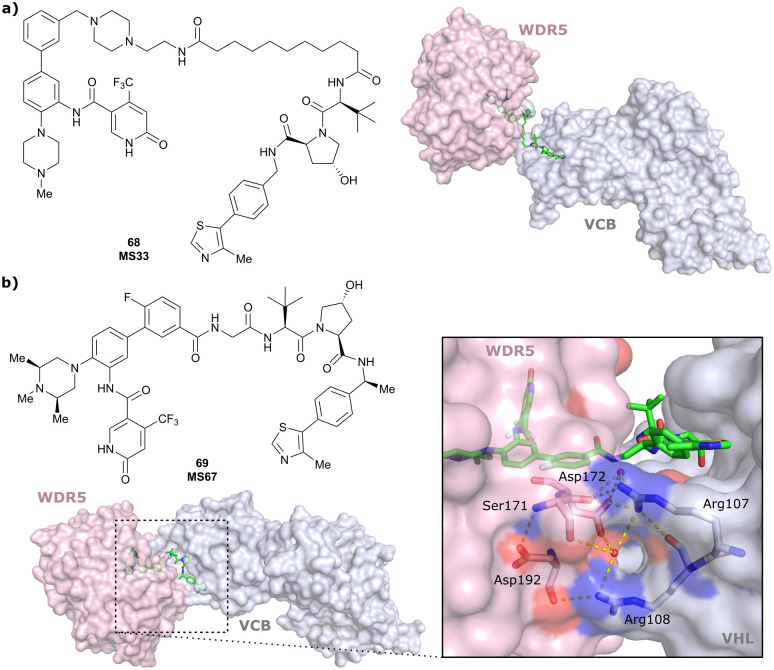
Ternary cocrystal structures of WDR5/MS33/VCB (PDB 7JTO) (a) and WDR5/MS67/VCB (PDB 7JTP) (b).^124^

WDR5 is a functional subunit of the mixed lineage leukaemia histone methyltransferase complex which contributes to sustaining haematological cancers and is overexpressed in some solid tumours.^154–156^ Though WDR5 selective inhibitors have been developed, their limited antiproliferative effects and lack of *in vivo* activity^124,157,158^ motivated exploration of therapeutic alternatives. Using structure-guided design, the laboratories of Wang and Jin recently developed selective WDR5 targeting PROTACs.^124^ Using inhibitor OICR-9429^158^ as WDR5 binding motif connected *via* its morpholine group to the N-terminus of VH032, degradation potency of an initial set of PROTACs was assessed, identifying MS33 (68) as a lead degrader. MS33 induced WDR5 degradation with DC_50_ = 260 nM and *D*_max_ = 71% and a ternary complex *K*_d_ of 520 nM and cooperativity *α* = 1.66 measured by ITC. The ternary cocrystal structure of MS33 bound to WDR5 and VCB, resolved at 1.7 Å, showed that MS33 is bridging WDR5 and VCB, recapitulating the ligand binding modes of the respective inhibitors, but only inducing few *de novo* PPIs as a consequence of MS33's long linker (Fig. 25a).

Based on this ternary cocrystal structure, several changes were envisioned to increase PPIs, PROTAC–protein interactions and binary binding of the ligands: (i) the linker was considerably shortened and the piperazinyl group used as tethering vector at OICR-9429 was removed; (ii) methyl groups were introduced at the 2- and 4-position of the methylpiperazinyl moiety of OICR-9429 to fully occupy WDR5's hydrophobic binding pocket; (iii) the phenyl ring of OICR-9429 was fluorinated to enhance interactions with Phe133 and Tyr191 of WDR5; and (iv) the benzylic methyl group (see Section 3.7) was introduced into VH032 to increase binding with VHL. From the 2^nd^ generation degraders, featuring solely one or more combinations of these modifications, the degrader featuring all modifications, MS67 (69), proved to be the most potent. Ternary binding affinity was improved to *K*_d_ = 52 nM compared to MS33, and cooperativity was increased to *α* = 2.74, as determined by ITC. According to the ternary cocrystal structure of WDR5/MS67/VCB (Fig. 25b), resolved at 2.1 Å, a novel, more extensive protein–protein interface formed between WDR5 and VCB due to rotation and translation of WDR5 relative to VHL.

Beside inducing more PPIs, such as hydrogen bonds between the side chains of Asp172 of WDR5 and Arg107 and Arg108 of VHL, MS67 created cross protein–ligand interactions, *e.g.*, hydrophobic contacts of the methyl and *t*Bu group of the VHL ligand with Phe149, Pro173 and Tyr131 of WDR5 and van-der-Waals contacts of WDR5 binder's fluorobenzyl moiety with Tyr112 and His110 of VHL. Cellular profiling of MS67 revealed improved cellular potency compared to MS33 (DC_50_ = 3.7 nM, *D*_max_ = 94%), cellular activity in a wider range of cancer cells and suppression of WDR5-mediated gene transcription. Furthermore, MS67 induced substantial WDR5 degradation and significant tumour growth inhibition in subcutaneous mouse xenograft models and induced prolonged mouse survival, indicating therapeutic potential of MS67 for treatment of WDR5-dependent tumours.^124^

**Fig. 26:**
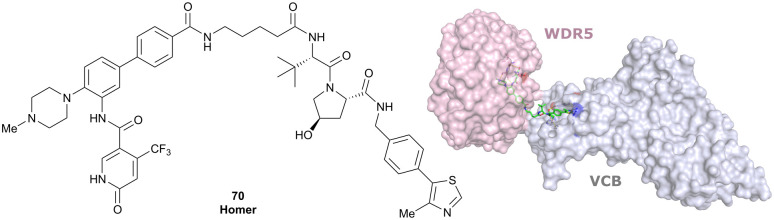
Ternary cocrystal structure of WDR5/Homer/VCB (PDB 7Q2J).

WDR5 has also been targeted for degrader development activities of the Structural Genomics Consortium (SGC) collaborative network and led to the disclosure of Homer (70, Fig. 26), a N-terminally linked VHL-recruiting WDR5 degrader with DC_50_ = 53 nM. Compared to MS67, Homer induces less PPIs between VCB and WDR5, as a result of the elongated linker, potentially rationalising the lower cellular potency of Homer.^125^

#### 4.2.7. Epidermal growth factor receptor (EGFR) targeting PROTACs

**Fig. 27:**
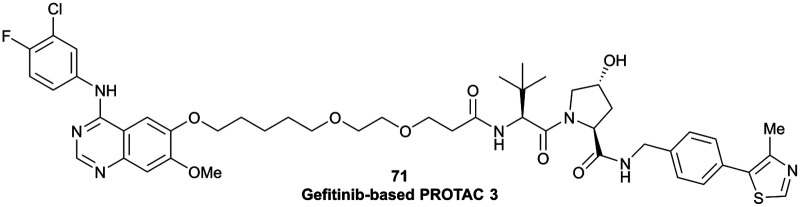
EGFR targeting Gefitinib-based PROTAC 3 (71).^122^

EGFR belongs to the class of receptor tyrosine kinases and is a transmembrane protein involved in the regulation of essential cellular processes, such as cell proliferation, metabolism and apoptosis.^159^ Overexpression or mutation of EGFR is associated with the development of a variety of solid tumours such as non-small cell lung cancer.^160^ Exploiting potent EGFR inhibitors as EGFR binding moiety, the Crews laboratory designed VHL-recruiting PROTAC degraders targeting EGFR tethered *via* the N-terminally amide linker vector.^122^ Using Genefitinib as POI ligand in degrader 71 (Fig. 27) induced almost complete, selective degradation of the Exon19 del and L858R mutations of EGFR, while sparing wild-type EGFR, showcasing that choice of the right ligand can allow differentiation between different mutational states of the target POI. Further EGFR targeting VHL-recruiting PROTACs have been developed since,^161–165^ including the recently disclosed covalent EGFR degrader CP17, with single-digit nanomolar DC_50_ values the most potent EGFR degrader reported to date.^165^

#### 4.2.8. Serum/glucocorticoid-inducible protein kinase (SGK) targeting PROTACs

**Fig. 28:**
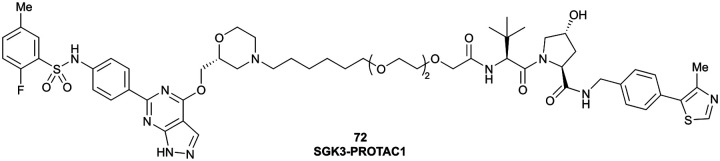
SGK3-selective VHL-recruiting degrader SGK3-PROTAC1 (72).^119^

SGK3 is a serine/threonine protein kinase that is activated downstream of growth factors by phosphorylation and that contributes to the regulation of ion channels, transcription factors and enzymatic activities.^166,167^ Though overexpression of SGK3 has been linked to several solid cancers, the precise mechanism of SGK3 regulation has yet to be elucidated.^168^ Starting from a pan-selective SGK inhibitor linked *via* an optimised ether-containing alkyl linker to VH032's N-terminus, the Ciulli and Alessi laboratories developed the SGK3 selective degrader SGK3-PROTAC1 (72, Fig. 28), inducing efficient SGK3 degradation with DC_50_ < 100 nM and *D*_max_ = 80%. Treatment with 72 led to reduced phosphorylation of the SGK3 native substrate NDRG1, and was able to counteract resistance to PI3K/Akt inhibition in cancer treatment.^119^

### 4.3. Structure-guided rational design of LHS thioether tethered VHL-recruiting PROTACs

Beyond the N-terminally linked VHL-based PROTACs described above, several other conjugation chemistries have been explored and various highly effective degraders have been developed accordingly over the years. In the following sections we outline the most explored alternative exit vectors.

**Fig. 29:**
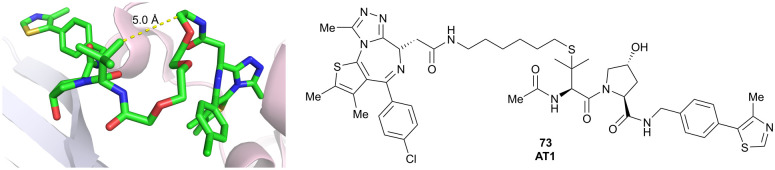
Design of LHS thioether conjugation vector from ternary crystal structure of VCB, MZ1 and Brd4^BD2^ (PDB 5T35) and structure of the optimised Brd4^BD2^ selective degrader AT1 (73).^102^

Based on the ternary crystal structure of MZ1 bound to VCB and Brd4^BD2^, a new linker vector potentially leading to increased target depletion selectivity towards Brd4 was identified.^102^ Maintaining both ligands’ binding modes, the *tert*-Leu group of VH032 was identified as in close contact to the conjugation point on the BET binding moiety (at ∼5 Å distance) and thus envisioned as tethering point for linker attachment (Fig. 29).^102^ Exchange of *tert*-Leu with the bioisosteric penicillamine in the VHL binder provided a thiol functionality at this position, suitable for linker attachment *via* thioether linkage. Using this strategy, a set of PROTAC degraders featuring both PEG-based and alkylic linkers of varying length were assessed regarding their binding affinity, degradation potency and selectivity. From this series, degrader AT1 (73, Fig. 29) showed clearly improved Brd4^BD2^ degradation selectivity both in immunoblotting and unbiased quantitative isobaric tagging mass spectrometry assays as compared to MZ1 and highest cooperativity for ternary complexes with Brd4^BD2^ (*α* = 7) as opposed to the BDs from Brd2 and Brd3 (1 < *α* < 4).^102^

This LHS thioether linkage has been included in the development of CRBN-VHL PROTACs, but led to degraders with only moderate activity of 20–30% CRBN degradation at 1 μM concentration in initial studies, while N-terminal amide linkage gave rise to a degrader of considerable higher potency (58, see Fig. 22b),^115^ highlighting the importance of exploiting different exit vectors in the design of novel VHL-recruiting PROTACs.

**Fig. 30:**
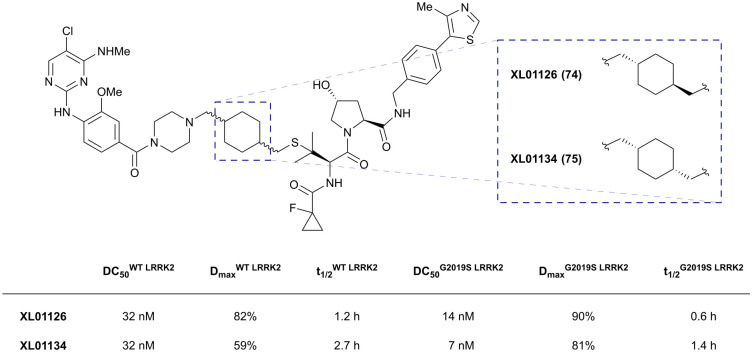
LRRK2 degraders XL01126 (74) and XL01134 (75).^126^

Very recently, the thioether linkage in VHL ligands has also been exploited by the Ciulli and Alessi laboratories in the development of Leucine Rich Repeat Kinase 2 (LRRK2) PROTAC degraders.^126^ LRRK2 is an attractive target for treating Parkinson's Disease, which has been associated with both increased LRRK2 activity and pathologic LRRK2 mutations.^169,170^ Starting from LRRK2 inhibitor HG-10-102-01 as LRRK2 binding moiety, a small set of PROTACs was designed to recruit cIAP, CRBN, or VHL, the latter connected either *via* the RHS phenolic or LHS thioether vector. LRRK2 degradation activity assessment in mouse embryonic fibroblasts identified three thioether-linked VH101-containing PROTACs as initial hit compounds, suggesting the thioether linkage as a privileged motif for designing next-generation LRRK2 PROTACs.^126^ A second compound series was designed to include modifications on the LRRK2 ligand, the VHL ligand and the linker moieties of the initial hit compounds, from which compounds XL01126 (74) and XL01134 (75) (Fig. 30), bearing a *trans*- or *cis*-functionalised cyclohexyl ring in the linker, respectively, emerged as most potent LRRK2 degraders.

While XL01134 showed a pronounced hook effect at concentrations >300 nM, degrader XL01126 did not lead to any hooking even at the highest concentrations tested, highlighting how subtle structural changes in the linker can impact the degrader's degradation profile. Though XL01126 had a >10-fold lower binding affinity to VHL, it featured considerably higher cooperativity of the ternary complex (*α* = 5.7 *vs.* 1.4 for XL01126*vs.*XL01134), thus rescuing the weaker binding affinity. In addition, the different linkers also impacted the permeability of these two PROTACs. Using Nano-BRET based target engagement assay in both permeabilised and live cell mode in parallel, the authors found that XL01126 was more permeable than XL01134, together rationalising its better degradation profile. Notably, XL01126 is both orally bioavailable (*F* = 15%) and able to penetrate the blood brain barrier, a feature that is perceived difficult to obtain with PROTAC degraders, highlighting its potential for *in vivo* therapeutic application.^126^

### 4.4. RHS benzylic tethered VHL-recruiting PROTACs

Within efforts to develop degraders for the androgen receptor (AR) in metastatic castration-resistant prostate cancer, and to improve their cellular potency compared to previously reported AR degraders, such as ARCC-4 (Table 1),^120^ the Wong group identified the methylated benzylic position at the RHS of VHL inhibitors as attractive tethering point for linker attachment.^94^

**Fig. 31:**
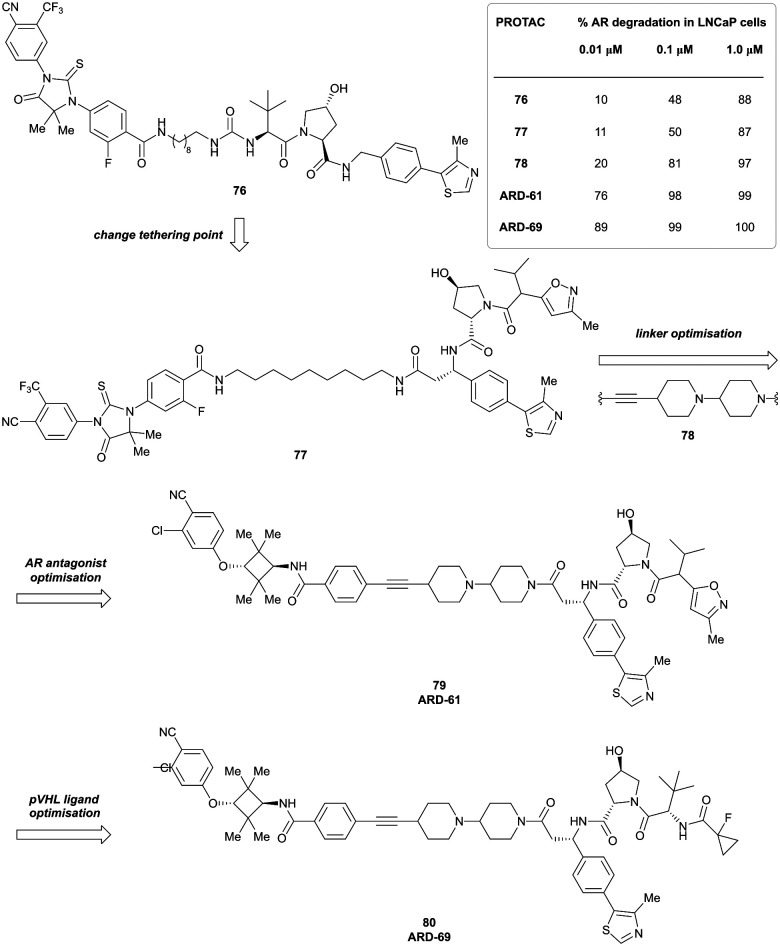
Development of AR degrader ARD-69 (80) featuring a benzylic exit vector between the VHL ligand and the linker.^94^

Towards this goal, they developed VHL binder 41 featuring a benzylic methyl acetamide group (see Section 3.7, Fig. 13) and incorporated it into AR targeting PROTACs *via* amide coupling with an amine containing linker. Benzylic AR degrader 77 (Fig. 31) degraded AR as effectively as its N-terminal analogue (76). Exchange of the flexible alkyl linker with a more rigid alkynyl-bis(piperidyl) linker (78) considerably improved degradation.^94^ Further improvement of degradation activity was achieved by variation of the AR binder to a potent AR antagonist developed by Pfizer,^171^ increasing degradation at 10 nM concentration from 20% to 76% (ARD-61, 79). Finally, exchange of the VHL binding moiety to a VH101 analogue generated the most potent AR-degrader 80, ARD-69, with sub-nanomolar DC_50_ values in prostate cancer cell lines and inducing considerable reduction in AR protein levels in xenografted tumour tissue in mice.^94^

**Fig. 32:**
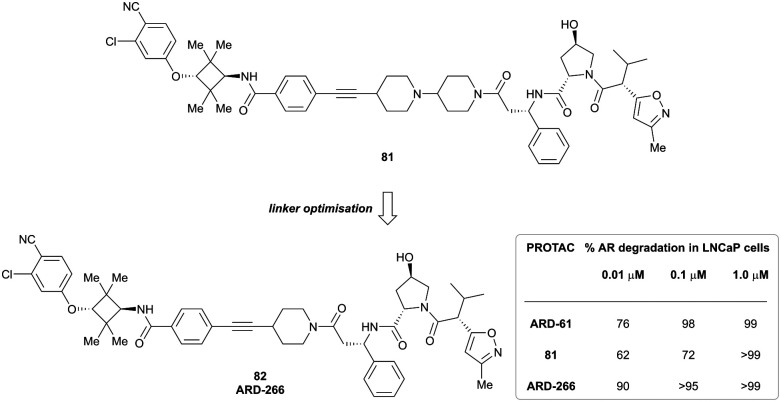
Development of AR degraders build from low-affinity VHL binding ligands featuring the linker exit vector in benzylic position.^172^

A follow-up study on related AR degraders focused on the influence of the binary binding affinity of the VHL-recruiting moiety towards VHL on degrader efficacy.^172^ For this purpose, the 4-methylthiazole unit from ARD-61 was replaced with smaller functionalities and the corresponding PROTACs were evaluated for their ability to reduce AR protein levels in prostate cancer cells.^172^ Effective reduction of AR protein levels was observed with all first series PROTACs, despite considerable differences in their binding affinity to VHL. For example, exchange of the 4-methylthiazolyl unit with hydrogen led to a 467-fold decrease in binding affinity of the isolated binder, but still induced 72% degradation at 0.1 μM concentration when incorporated in the corresponding PROTAC degrader 81 (Fig. 32). Optimisation of the linker by removing one piperidyl group leading to degrader ARD-266 (82) recovered and even exceeded the cellular potency of its parent degrader ARD-61 and competes in potency with the much larger AR degrader ARD-69, further exemplifying the impact of ternary complex formation in rescuing low-affinity VHL binders for PROTAC development.

More recently, another example of VHL-recruiting PROTACs featuring a benzylic exit vector was reported by the Ciulli laboratory/Boehringer Ingelheim collaboration in the design of orally bioavailable SMARCA2 selective degraders.^103^ Convenience of administration, ideally oral administration, is highly relevant for therapeutic development and applicability of PROTAC degraders, and has been – apart from very few reports of orally bioavailable VHL-recruiting PROTACs^103,126,173^ – limited to CRBN-recruiting degraders.^174^ To access orally bioavailable SMARCA2 degraders, a novel SMARCA2/4 BD binder featuring a bare minimum of hydrogen bonds was designed and linked *via* either the phenolic or benzylic position to VHL recruiters.^103^ For the degraders exploiting the benzylic exit vector, novel synthetic routes were developed, as previous examples relied on introducing an additional amide bond which is contraindicated when attempting oral bioavailability.

**Fig. 33:**
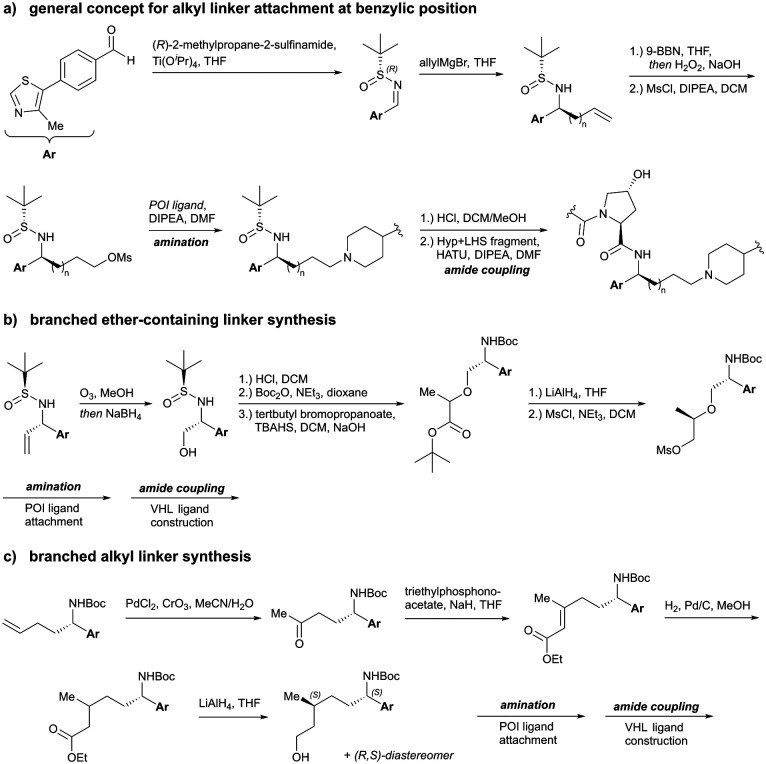
Synthetic routes to attach alkyl linkers (a), branched ether-containing linkers (b) and branched alkyl linkers (c) to the benzylic position of VHL binders.^103^

As a general method to access linear alkyl linkers at the benzylic position, 4-(4-methylthiazol-5-yl) benzaldehyde or a derivative was converted in the corresponding *tert*-butyl sulfinamide followed by stereoselective 1,2-addition of a terminal alkenyl Grignard reagent. Hydroboration–oxidation formed a terminal alcohol, which, after being transformed into the corresponding mesylate, underwent amination with the POI ligand. The VHL binding moiety was subsequently completed by cleavage of the *tert*-butyl sulfinyl group followed by HATU-mediated amide coupling with the LHS-Hyp building block (Fig. 33a).^103^

To introduce branching in α-position from ethers, alcohols generated as described above underwent S_N_2 reaction with *tert*-butyl 2-bromopropanoate followed by reduction with LiAlH_4_ (Fig. 33b). The branched all-alkyl linker of ACBI2 was synthesised starting from a terminal alkene made using the general strategy, which was transformed in the corresponding methyl ketone by Pd-mediated oxidation. Horner–Wadsworth–Emmons olefination with triethyl phosphonoacetate followed by reduction of the alkene and subsequent reduction of the ester gave diastereomers of the desired alcohol, which were separated by supercritical fluid chromatography (SFC) prior to mesylation and coupling with the POI ligand (Fig. 33c).^103^

**Fig. 34:**
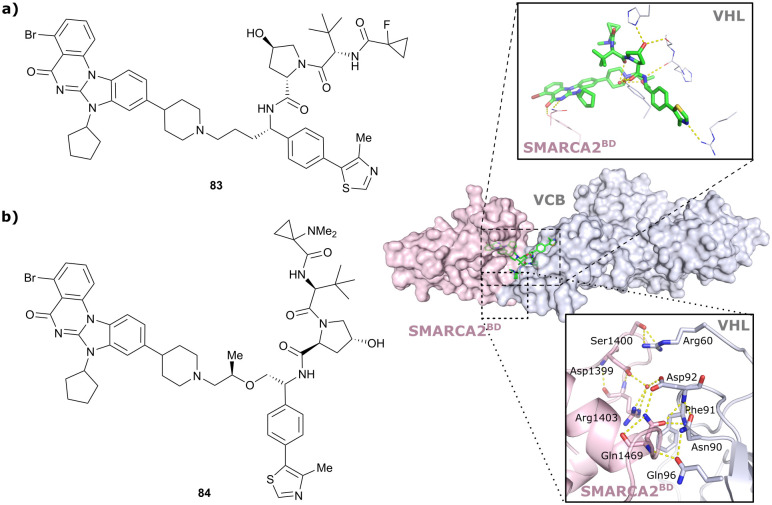
Initial benzylic tethered SMARAC2/4 targeting PROTAC 83 (a) and SMARCA2-selective PROTAC 84 including visualisation of ionic *de novo* PPIs (yellow dotted lines) in the ternary crystal structure of degrader 84 bound to SMARCA2^BD^ and VCB (PDB 7Z76).^103^

Compared to an initial SMARCA2 selective degrader with a phenolic vector on the VHL ligand, the corresponding benzylic degrader 83 (Fig. 34a) featured a higher buried surface area in its ternary cocrystal structure, an increased ternary complex half-life, improved microsomal stability and lower clearance, but failed to discriminate between SMARCA2 and SMARCA4 degradation.

Aiming at restoring SMARCA2 selectivity and further improving pharmacokinetic properties, a set of analogues with varied short alkyl and ether-based linkers including branched ones was assessed. Slightly elongated linkers restored the desired SMARCA2 selectivity and branching by adding a methyl group to the linker improved cell permeability. Ternary crystal structure elucidation of degrader 84 bound to VCB and SMARCA2 revealed extensive *de novo* PPIs, including interaction of the SMARCA2-specific residue Gln1469 with Phe91 and Asp92 of VHL (Fig. 34b) potentially accounting for its excellent SMARCA2 selectivity. However, degrader 84 still showed a high efflux ratio preventing oral bioavailability.

**Fig. 35:**
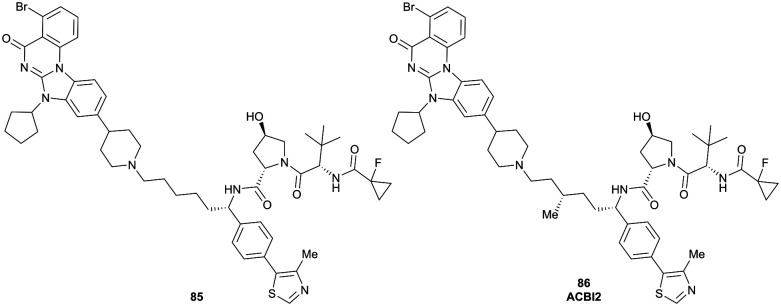
Further optimised orally bioavailable SMARCA2 degraders 85 and ACBI2 (86) featuring benzylic linker attachment points.^103^

To further optimise pharmacokinetic properties, alkyl linkers of higher lipophilicity were studied, leading to the development of degrader 85 (Fig. 35), which yielded measurable oral bioavailability (F% p.o. 3%) and induced 80% reduction of SMARCA2 levels after oral treatment in xenografted mice. Incorporation of a branching methyl group in the linker of degrader ACBI2 (86, Fig. 35) induced a more compact solution conformation and further improved efflux, resulting in substantial increases in oral bioavailability (F% p.o. 22%). ACBI2 features a DC_50_ at 18 h of 1 nM for SMARCA2, a 30-fold selectivity for SMARCA2 over SMARCA4 degradation in several cell lines, and induced significant cytotoxicity in NCI-H1568 lung cancer cell lines (IC_50_ of 7 nM) and inhibition of tumour growth in mouse lung cancer xenograft models.^103^ The development of ACBI2, a highly potent orally available VHL-recruiting SMARCA2-selective degrader, illustrates the potential of rational, structure-based and pharmacokinetically-driven design in the development of orally administrable VHL-based PROTAC degraders with therapeutical potential.

### 4.5. RHS phenolic tethered VHL-recruiting PROTACs

Linker attachment at the RHS aromatic moiety through formation of a phenol ether was identified early on in the development of HaloPROTACs^100^ (Section 4.1) as potential conjugation vector in VHL-recruiting degraders and has been exploited in numerous studies since.

**Fig. 36:**
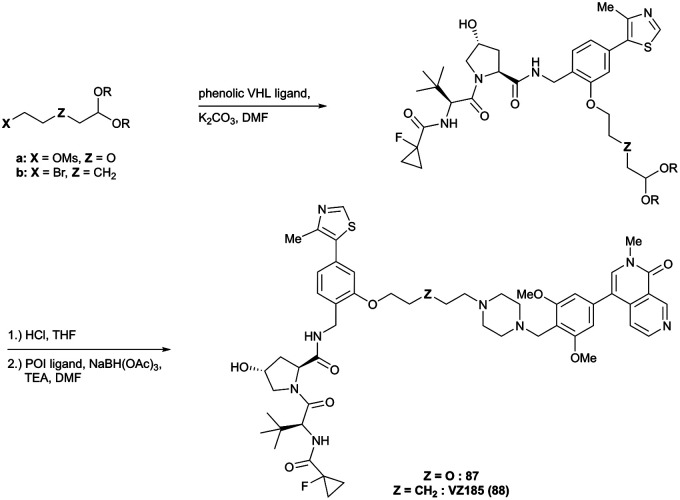
Development of Brd7/9 selective VHL-recruiting degraders 87 and VZ185 (88) featuring phenolic linker vectors.^112^

Following the unsuccessful use of phenolic VHL ligand conjugation in their early homo-PROTAC study (Section 4.2), the Ciulli laboratory exploited the phenolic exit vector of VHL ligands in PROTACs aiming to degrade the bromodomain-containing proteins BRD7/9.^112^ BRD7/9 are two mutually exclusive subunits of the chromatin BAF remodeller complexes that had previously been linked to leukaemia and synovial sarcomas.^175,176^ The initial prototypic N-terminally amide ligated VHL-recruiting PROTACs exhibited negative cooperativity and did not induce measurable protein degradation in cells.^112^ This led the team to switch conjugation strategy to an ether bond that was formed by S_N_2 reaction of mesylated or brominated linkers with the phenolic group of the VHL binder. Subsequent acid-mediated hydrolysis of the linker's terminal acetal to the corresponding aldehyde followed by reductive amination with the piperazine moiety of the BRD7/9 ligand formed the desired phenolic-tethered VHL-recruiting PROTACs (Fig. 36). The first set of such phenolic PROTACs comprised VHL binders with LHS acetyl, cyanocyclopropyl and fluorocyclopropyl amides, thus derivatives of VH032, VH298 and VH101, respectively, featuring alkoxy linkers of various length. Profiled against BRD7 and BRD9 degradation, increased degradation activity towards BRD9 was observed for shorter and more lipophilic linkers, while BRD7 levels remained basically untouched. Furthermore, cellular potency increased in the order acetyl < cyanocyclopropyl amide < fluorocyclopropyl amide as LHS group of the VHL ligand, reflecting the increasing binding affinity towards VHL of the related free inhibitors as well as potential better shielding of the LHS *tert*-Leu amide bond HBD group, minimising desolvation penalties and maximising cell permeability, an SAR trend previously shown within the context of the VHL inhibitor alone.^62^

Based on this SAR data, further systematic optimisation efforts focused on the linker length and nature, thus lipophilicity, and variation of the BRD7/9 ligand while keeping the fluorocyclopropyl amide containing VHL-recruiting moiety.^112^ In accordance with SAR from the first series of PROTACs, compounds 87 and 88 (VZ185) featuring short 5-atom linker containing one (87) or no (VZ185) oxygen atom (Fig. 36), led to enhanced degradation of BRD9 and now also dialled-in some BRD7 degradation. VZ185, featuring nanomolar DC_50_ values, is cytotoxic in BRD7/9 sensitive tumour cell lines and selectively depletes BRD7/9 levels while leaving other BD containing protein unaffected, thus qualifying as chemical probe.^112^

A subsequent study by a team at Promega and the Ciulli laboratory retrospectively analysed the developed series of Brd7/9 PROTACs to reveal mechanistic determinants of the chemical series optimisation that were important to translating into functional outcomes underlying BRD7 and BRD9 protein degradation and the discovery of VZ185 as the optimal degrader and chemical probe.^177^ This study highlighted that the significant improvements in cellular degradation activities observed between the compounds of series 2 and series 1 were underpinned by the decision to maintain fixed the VH101-phenol as the conjugatable VHL ligand during the optimisation. This decision drove substantial improvements in cell permeability, while retaining favourable ternary complex formation. In particular, VZ185 was not the most cell permeable, nor the most cooperative PROTAC, but was the one that induced the most ubiquitination on BRD9.^177^

**Fig. 37:**
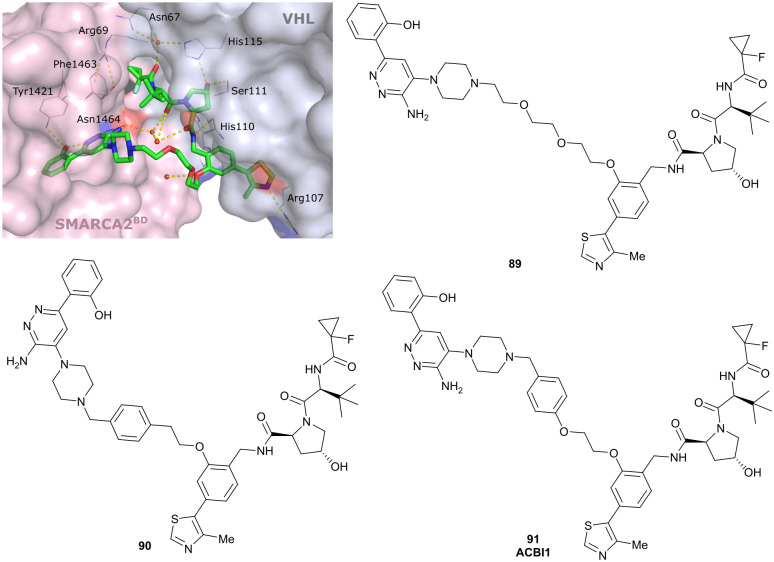
Structure-based design of phenolic tethered SMARCA2/4 degraders: cocrystal structure of the initial degrader 89 bound to VCB and SMARCA2^BD^ (PDB 6HAY), and structures of degraders 90 and ACBI1 (91) derived from structure-guided design.^113^

Leveraging the privileged VH101-phenol as VHL ligase recruiting moiety, the phenolic linkage has been exploited in the structure-guided design of degraders targeting the BAF ATPase subunits SMARCA2 and SMARCA4 as potential therapeutics for BAF ATPase dependent cancer.^113^ Capitalising on the cocrystal structure of the initial prototype degrader 89 binding to VCB and SMARCA2^BD^, attractive regions for degrader optimisation were identified. Degrader 89 consisted of a SMARCA BD ligand connected *via* three PEG units to the VH101-phenol VHL ligand. Inspection of the ternary complex cocrystal structure revealed that all HBDs of degrader 89 were involved in PROTAC–protein interactions and favourable *de novo* PPIs were induced around the fluorocyclopropyl amide group of the PROTAC (Fig. 37). Based on these observations, PROTAC optimisation focused on the alkoxy linker which was found to be collapsed at the ternary complex PPI interface and hypothesised to be contributing to the low passive permeability of 89.

To increase conformational restraint, potentially enable π-stacking with Tyr98 of VHL and to reduce the polarity of the linker, a 1,4-substituted benzyl group was introduced into degrader 90 to rigidify the linker, leading to better molecular recognition within the ternary complex (Fig. 37). The newly introduced group, as designed, formed the envisioned π-stacking and improved both permeability and ternary complex cooperativity and stability. Ultimately, extending the linker by one oxygen atom, thus matching the linker length of 89 and further releasing linker strain, resulted in ACBI1 (91, Fig. 37), featuring drastically improved ternary complex cooperativity and affinity, and PROTAC permeability. ACBI1 induced selective, fast and complete degradation of the engaged targets SMARCA2, SMARCA4 and PBRM1 in MV-4-11 cancer cells with DC_50_ of 6 nM and 11 nM for SMARCA2/4, respectively. ACBI1 recapitulated sensitivities to SMARCA2/4 degradation in cancer cell lines, in a manner not achieved by bromodomain inhibition.^113^ Emphasising the power of iterative structure-guided design, ACBI1 has been developed *via* focused and precise structure-guided design modifications, requiring only a limited number of analogues during the medicinal chemistry optimisation campaign, thus avoiding laborious unguided exploration of chemical space.

**Fig. 38:**
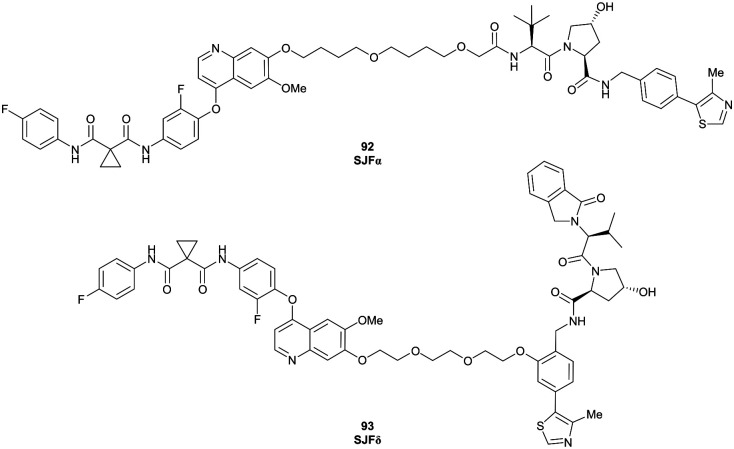
Isoform selectivity of p38 degraders driven by differing linker tethering vectors of the VHL-recruiting ligand.^178^

The Crews laboratory discovered isoform-selective PROTACs against members of the p38 mitogen-activated protein kinase (MAPK) family *via* PROTACs exploring different vectors out of the VHL ligand.^178^ Comparing LHS N-terminally amide bond tethered and RHS phenolic tethered VHL-recruiting PROTACs comprising foretinib as pan-selective warhead for p38 binding, preferential degradation of the p38α-isoform was found for representatives of the N-terminally-tethered PROTAC series, while only partial degradation of the δ isoform and no degradation of β and γ isoforms was observed for the most potent and selective degrader SJFα (92). In contrast, phenolic tethered degrader SJFδ (93), featuring a shorter linker than SJFα, induced efficient depletion of p38δ levels while leaving α, β and γ isoforms untouched (Fig. 38). A combination of cellular pull-downs and *in silico* molecular dynamics simulation studies and selective mutations were used to hypothesise that SJFδ's selectivity towards inducing p38δ degradation likely emerge from greater favourability of the p38δ/SJFδ/VHL ternary complex formation, as opposed to p38δ/SJFα/VHL ternary complex formation, resulting from stabilising PPIs between VHL and p38δ in presence of SJFδ, which are not accessible in the differing conformation of p38δ relative to VHL when recruited by SJFα.^178^

**Fig. 39:**
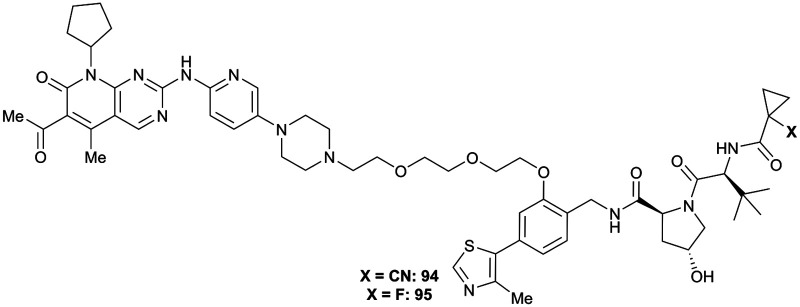
Optimised CDK6 degraders 94 and 95 featuring phenolic tethering vector of the VHL-recruiting ligand.^179^

Exploring the chemical space of cyclin-dependent kinase 4/6 (CDK4/6) PROTACs, the laboratories of Gütschow and Krönke assessed degraders build from CRBN, VHL, cIAP and MDM2 ligands as E3 recruiting unit, various linkers and Palbociclib as CDK4/6 binder.^179^ Palbociclib is an FDA and EMA approved inhibitor of CDK4/6 for the treatment of advanced and metastatic breast cancer.^180^ Two series of VHL-recruiting PROTACs were developed, featuring either LHS N-terminal amide or RHS phenolic tethering to alkyl- and alkoxy linkers of various length. In contrast to the N-terminal amide series, in which each representative PROTACs induced pronounced depletion of both CDK4 and CDK6 protein levels, preferential CDK6 degradation was observed among the phenolic-linked PROTACs. Further optimisation of these degraders by enhancing VHL binding affinity led to highly potent and selective CDK6 degraders 94 and 95 (Fig. 39), with nanomolar DC_50_ values and suppression of CDK6 levels for up to 96 h.^179^

**Fig. 40:**
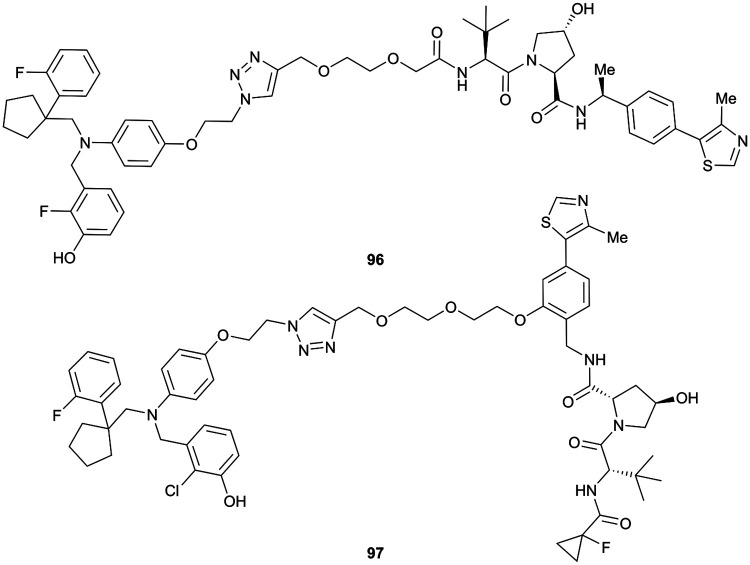
ERα targeting VHL-recruiting PROTACs derived from DNA-encoded library screening.^181^

In a recent approach to develop novel oestrogen receptor α (ERα) degraders, an ERα antagonist derived from DNA-encoded chemical library screening followed by off-DNA hit optimisation has been incorporated into a series of PROTAC molecules featuring CRBN, VHL and IAP binders with different linkage vectors as E3 ligase recruiting moieties.^181^ Using the Huisgen 1,3-dipolar cycloaddition, commonly referred to as click reaction, the azide-functionalised ERα binder was coupled with E3 ligase binders featuring PEG linkers of different length terminated with an alkyne functionality. The VHL-based PROTACs of both the amide-linked and phenoxy-linked series induced ERα degradation at sub-micromolar level in multiple cell lines, with compounds 96 and 97 (Fig. 40), both featuring (poly)ethylene glycol linker, being the most potent representatives of each class. In contrast to similar behaviour of 96 and 97*in vitro*, solely the phenoxy-linked degrader 97 showcased considerable tumour growth inhibition in mouse xenograft models, possibly due to lower *in vivo* clearance of 97,^181^ highlighting the relevance of exploiting different exit vectors at the VHL ligand when designing PROTACs.

VHL-recruiting PROTAC degraders exploiting a phenolic exit vector have been further trialled in the development of VHL-Homo-PROTACs,^114^ VHL-CRBN degraders^115^ and Bruton-Tyrosinkinase (BTK) degraders,^182^ but performed inferiorly than degraders featuring alternative linker exit vectors.

### 4.6. Chemistry innovation in PROTACs exemplified with VHL-based molecules

To aid developing PROTAC degraders for new target POIs and expand the sampled chemical space, it is important to explore alternative and unusual linkage chemistry and motifs. Efforts in this context will be herein exemplified by chemistry innovations developed within the realm of VHL-based degraders.

#### 4.6.1. Amide-to-ester conversion

**Fig. 41:**
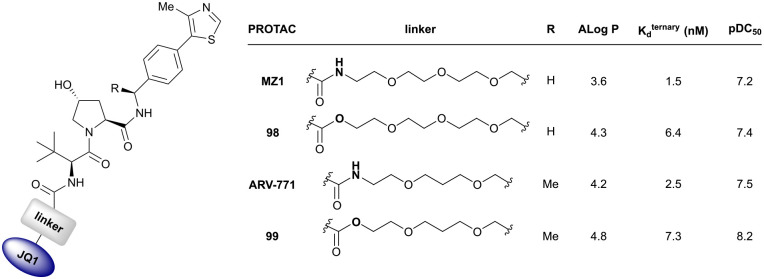
Amide-to-ester conversion BET protein targeting PROTACs recruiting VHL leading to improved pharmacokinetics and cellular potency.^108^

Analysing the influence of structural features on PROTAC permeability of previously reported VHL-recruiting degraders, the groups of Lokey and Ciulli found a strong influence of the environment around HBDs, and in particular intramolecular hydrogen bonds (IMHBs) on the respective lipophilic permeability efficiency.^183^ In particular, solvent-exposed amide functionalities are known to contribute to low cell permeability due to the highly unfavourable energetic penalties associated with desolvating the highly polar amide bonds. To alleviate this, substitution of an amide group with an ester functionality was trialled in model VHL binders, resulting indeed in improved cellular permeability, but reducing binding affinity to VHL when the ester was located in close proximity to the Hyp binding site.^183^ Further evaluation of VHL-based amide- and ester-containing PROTAC-like model compounds showed significantly increased cellular permeability upon amide to ester conversion and revealed the possibility of shielding exposed HBD through ligand-to-linker IMHBs of the ligand's amide with a 5-atom distant oxygen atom in an alkoxy linker.^108^ Implementing the ester-linkage as connecting chemistry between the BET ligand and the linker in derivatives of the BET degraders MZ1 and ARV-771 resulted in improved permeabilities, and greater degradation potency and cytotoxicity of the ester derivatives 98 and 99 compared to their amide molecular matched pairs (Fig. 41).^108^

#### 4.6.2. Macrocyclic PROTAC

**Fig. 42:**
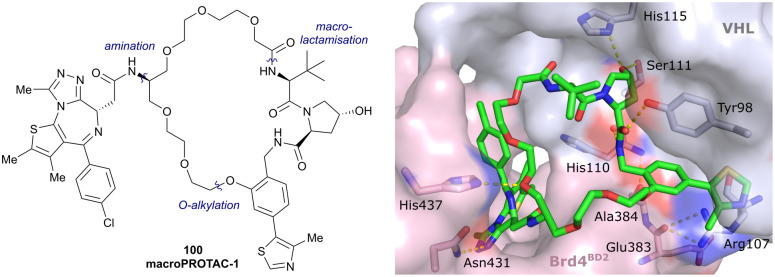
Synthetic strategy to access macroPROTAC-1 (100) and cocrystal structure of macroPROTAC-1 bound to VCB and Brd4^BD2^ (PDB 6SIS).^110^

Macrocyclisation is a powerful design strategy to constrain the conformation of a ligand molecule to its bound form, that can increase binding affinity and selectivity by reducing the energetic penalty associated with the binding process, and *via* forming additional interactions.^184–186^ Applying this concept in PROTAC design, a macrocyclic derivative^110^ of the BET degrader MZ1 was designed based on the VCB/MZ1/Brd4^BD2^ ternary complex crystal structure.^102^ Building on the molecular architecture of MZ1, an additional PEG_3_-based linker connecting the phenolic tethering point of the VHL ligand with the α-position of the tethering amide group of the BET binding motif, was found to be the preferred linker length based on MD simulations on the MZ1 cocrystal structure.^110^ Further calculations also predicted the most favourable conjugation pattern at the newly introduced stereocentre, based on considerations of conformational energy and compatibility with ternary complex formation. Synthesis of the designed macroPROTAC-1 (100) was achieved from a trifunctionalised PEG linker *via O*-alkylation of the phenolic position with the mesylated alcohol. Subsequent macrolactamisation of the deprotected carboxylic acid of the linker with the N-terminal amine of the VHL binder and lastly amide bond formation between the acid group of JQ1 and the amine functionality of the linker yielded the final macrocyclic PROTAC (Fig. 42). Relative to MZ1, macroPROTAC-1 features increased differential cooperativity between BET bromodomains which can be explained with the filling of the cavity between VHL and the ZA-loop of Brd4^BD2^ by the newly introduced linker component in the otherwise maintained overall binding mode of the PROTAC compared to that of MZ1, as evidenced by a novel ternary complex cocrystal structure (Fig. 42). The cellular potency of macroPROTAC-1 was similar to MZ1 with regards to both degradation efficiency and cytotoxicity, despite a 12-fold reduced binary binding affinity with Brd4^BD^,^110^ a property worth factoring into the design of future macrocyclic PROTACs.

#### 4.6.3. Trivalent PROTACs

**Fig. 43:**
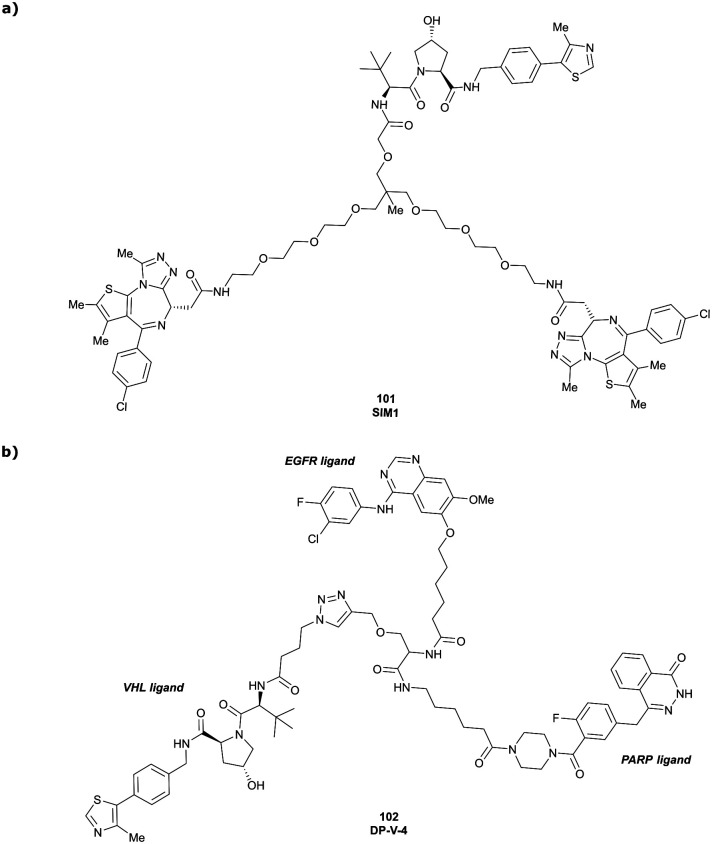
Trivalent PROTACs. Trivalent degrader SIM1 (101) targeting both BDs of Brd4 *via* two incorporated BET ligands (a),^187^ and trivalent degrader DP-V-4 (102) simultaneously targeting EGFR and PARP proteins (b).^189^

Hypothesising that increasing binding valency of PROTACs might enhance degradation and target selectivity, the Ciulli laboratory at Dundee in collaboration with the Daniels team at Promega developed trivalent BET targeting degraders by fusing a bivalent BET PROTAC degrader with a bivalent BET inhibitor.^187^ Analyses of the cocrystal structures of MZ1^102^ bound to VCB and Brd4^BD2^ and of the bivalent BET inhibitor MT1^188^ in complex with two molecules of Brd4^BD2^, identified solvent-exposed central portions of their linkers as suitable tethering points for the envisioned tripodal linker. Using 1,1,1-tris(hydroxymethyl) ethane as branching point of the linker scaffold, trivalent PROTACs were designed that featured two instances of JQ1 as BET ligand and either VH032 or pomalidomide as VHL and CRBN-recruiting ligands, respectively, joined by PEG-linkers of various lengths.^187^ Trivalent VHL-based PROTACs were identified as much more potent degraders than CRBN-based ones. Trivalent PROTAC SIM1 (101, Fig. 43a) was identified as the most potent degrader, requiring simultaneous engagement of all three functional valences, and featuring picomolar DC_50_ values, with preference for Brd2, and improved cellular potency and increased downstream functional activity compared to bivalent BET degraders MZ1 and ARV-771. Extensive biophysical analysis revealed that SIM1 formed a 1 : 1 : 1 complex with VHL and the BET proteins, by intramolecularly engaging their BD1 and BD2 bromodomains simultaneously in *cis* with high avidity, added to cooperative recruitment of VHL. The combined binding avidity and cooperativity led to the formation of highly stable complexes with prolonged residence times and highly efficient ubiquitination, accounting for the higher potency of SIM1 compared to bivalent degraders. Despite its large molecular weight, SIM1 showed comparable cell permeability to bivalent compounds, as well as low clearance and long half-lives after intravenous and subcutaneous injection in mice,^187^ qualifying SIM1 as chemical probe for both *in vitro* use and *in vivo* use.

In another study, related but conceptually distinct trifunctional PROTACs featuring ligands for two different target POIs were developed as a strategy to overcome the shortcomings of single-target drugs such as emerging drug resistance. Dual PROTACs were built from Gefitinib and Olaparib as EGFR and poly(ADP-ribose)polymerase (PARP) binders, respectively, and an E3 recruiting ligand linked to either the amino acid Tyr or Ser serving as a tri-functional linker for sequential attachment of each terminal recruiting moiety (Fig. 43b).^189^ The resulting CRBN- and VHL-recruiting prototype trifunctional PROTACs, such as DP-V-4 (102), induced simultaneous degradation of EGFR and PARP and weak antiproliferative activity, as such establishing a basis for development of dual-targeting degraders.

### 4.7. Degradation technology platforms based on VHL-recruiting PROTACs

#### 4.7.1. Innovations towards spatiotemporal control of VHL-recruiting PROTAC activity

With the progression of development of highly potent PROTAC degraders qualified as chemical probes and bearing potential for therapeutic applications, design of methods allowing conditional control of PROTAC function gained the attention of the chemical biology community. Spatiotemporal control of PROTAC function is highly desirable for targeted therapy, as mechanisms directing PROTAC activity towards cancerous tissue could reduce undesirable off-target effects, such as cytotoxicity towards healthy cells. Current approaches toward spatiotemporal control of VHL-recruiting PROTAC activity are based on either control of the PROTAC's activity by an external stimulus, such as light,^190–192^ or preferential enrichment of inactive PROTACs in cancerous tissue, where the compound is transformed into an active degrader.^193,194^

**Fig. 44:**
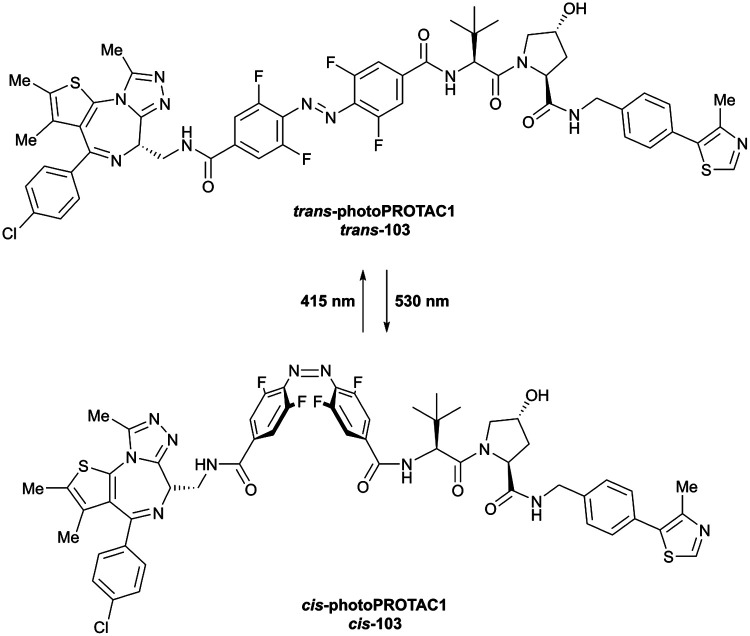
Reversible switching between an active, degrading *trans*-isomer and an inactive *cis*-isomer of photoPROTAC1 (103) upon irradiation with 415 nm *viz.* 530 nm.^190^

The groups of Crews and Carreira developed photo-switchable PROTACs containing azobenzene linkers as a strategy towards spatiotemporal control of induced POI degradation.^190^ In their design strategy, they used *ortho*-tetrafluorobenzene as a bio-compatible photoinduced switch, hypothesising that its *trans*-conformation would induce tripartite binding with POI and E3 ligase, while its shorter *cis*-conformation linker would not do so as effectively. Starting from a ARV-771 derived lead structure, incorporation of the azobenzene moiety *via* amide coupling with the amine of VH032 and an amine-derivative of JQ1 generated bistable photoPROTAC1 (103) with a linker length of 11 Å and 8 Å for the *trans*- and *cis*-isomers, respectively, and n–π* absorption bands at 415 nm (*cis*–*trans* isomerisation) and 530 nm (*trans*–*cis* isomerisation) (Fig. 44). *In vitro* tests showed robust degradation of Brd2 with *trans*-103, while *cis*-103 was incapable of inducing POI degradation. As anticipated, incubation of *cis*-103 under 415 nm irradiation induced considerable Brd2 degradation,^190^ indicating that light induces spatiotemporal control of POI degradation in azobenzene-containing photoPROTACs. A related approach for photochemical control of protein degradation *via* CRBN-based PROTACs was developed around the same time by the Trauner group.^195^

An alternative approach relying on light as external stimulus inducing spatiotemporal control over PROTAC activity involves deactivation of degraders by caging with photolabile groups, whose light-mediated cleavage releases the active degraders. Several photocaging strategies have been developed both for CRBN and VHL-recruiting degraders.^191,192,196–199^

**Fig. 45:**
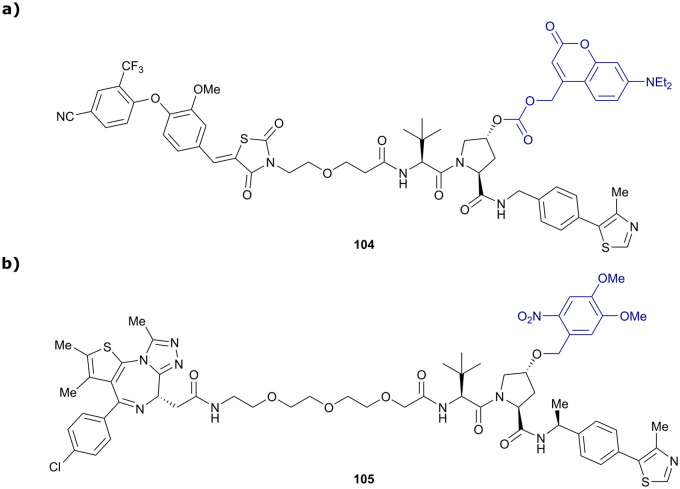
Photocaged PROTACs. Photocleavable DEACM-caged ERRα degrader 104 (a),^191^ and photocleavable DMNB-caged BET degrader 105 (b).^192^

For VHL-recruiting PROTACs, attachment of a bulky group to the hydroxyl group of the Hyp motif blocked binding with VHL, thus inactivating the corresponding degrader. Using this strategy, the Deiters laboratory developed photocleavable ERRα degraders using diethylamino coumarin (DEACM) as caging group attached to the Hyp hydroxyl *via* carbonate linkage.^191^ While the caged degrader 104 was completely inactive, photolysis with <405 nm light released the corresponding active degrader within 3 min (Fig. 45a), which induced significant reduction in ERRα levels in cancer cells.

Following the same strategy, the Tate laboratory introduced 4,5-dimethoxy-2-nitrobenzyl (DMNB) as caging group for Hyp in a BET degrader structurally related to MZ1. While the caged PROTAC 105 was inactive, irradiation at 365 nm for 1 min was sufficient to release the corresponding active degrader inducing Brd4 degradation at concentrations >100 nM (Fig. 45b).^192^

**Fig. 46:**
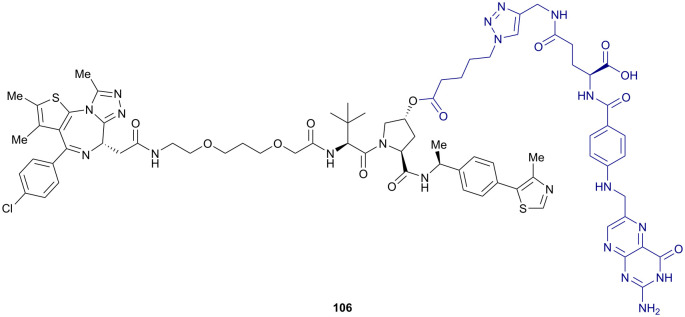
Folate-caged BET degrader 106.^193^

Apart from decaging methodologies relying on light, caging strategies based on enzyme-mediated removal of the caging group in cancerous cells have been established. Exploiting the overexpression of folate receptor α (FOLR1) in many cancer types, folate-caged degraders have been designed,^193^ envisioned to be transported preferentially into cancer cells, where intracellular hydrolase catalysis activates the degrader by release of the folate caging group. As a proof-of-concept, the laboratories of Jin and Wei attached folate to Hyp of ARV-771 using a hydrolysable ester bond, yielding degrader 106 (Fig. 46). While efficient degradation of BET proteins and cytotoxicity were induced by 106 in several cancer cell lines, considerably lower activity was detected in non-cancerous cells. Extending the applicability of this strategy, FOLR1-dependent degradation has further been demonstrated for folate-caged VHL-recruiting PROTACs targeting MEK1/2 and anaplastic lymphoma kinase (ALK) proteins.^193^

**Fig. 47:**
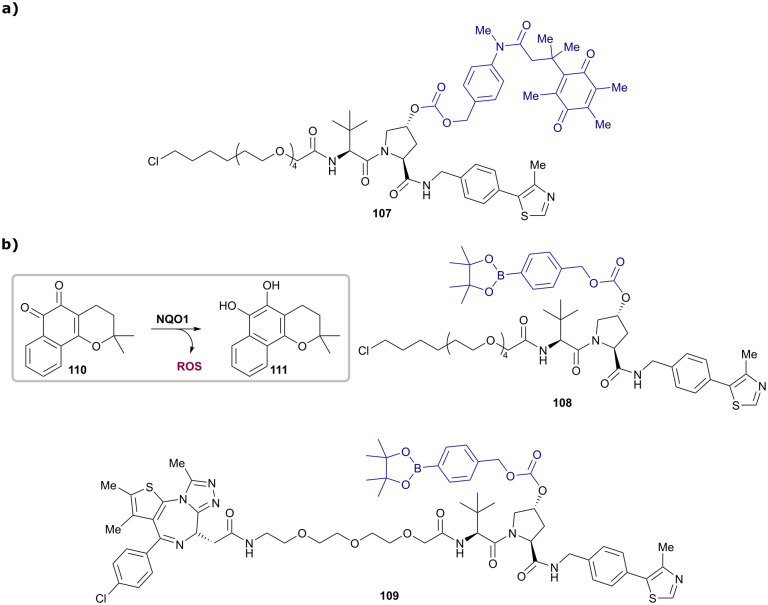
Enzymatic activated PROTACs. Quinone-caged HaloPROTAC 107 activated by NOQ1-mediated reduction (a) and generation of ROS *via* NOQ1-mediated reduction of β-lapachone (110) activating ROS-responsive HaloPROTAC 108 and BET degrader 109 (b).^194^

Merging enzyme-response chemistry with PROTAC technologies, the overexpression of the NAD(P)H quinone dehydrogenase 1 (NQO1) enzyme in cancer cells has inspired the design of trimethyl-locked quinone cages attached to Hyp of VHL-recruiting PROTACs, envisioned to release the active PROTAC upon quinone reduction by NQO1. As an example, an NQO1-responsive HaloPROTAC 107 has been synthesised by carbonate ester formation from the HaloPROTAC's Hyp and a quinone-carbonochloridate moiety. NQO1 was able to efficiently cleave the caging group of 107 (Fig. 47a), leading to considerable depletion of HaloGFP protein levels, while NQO1 knock-down fully suppressed POI degradation. Alternatively, arylboronic acids, which can be removed by reactive oxygen species (ROS), were introduced as caging groups in ROS-responsive PROTACs 108 and 109 (Fig. 47b). Co-treatment with either 108 or 109 and β-lapachone (110), known to excessively generate ROS in living cells upon reduction to 111 by NQO1,^200^ induced efficient degradation of the respective POI in cancer cells, while negligible effects were observed in noncancerous cells.^194^

**Fig. 48:**
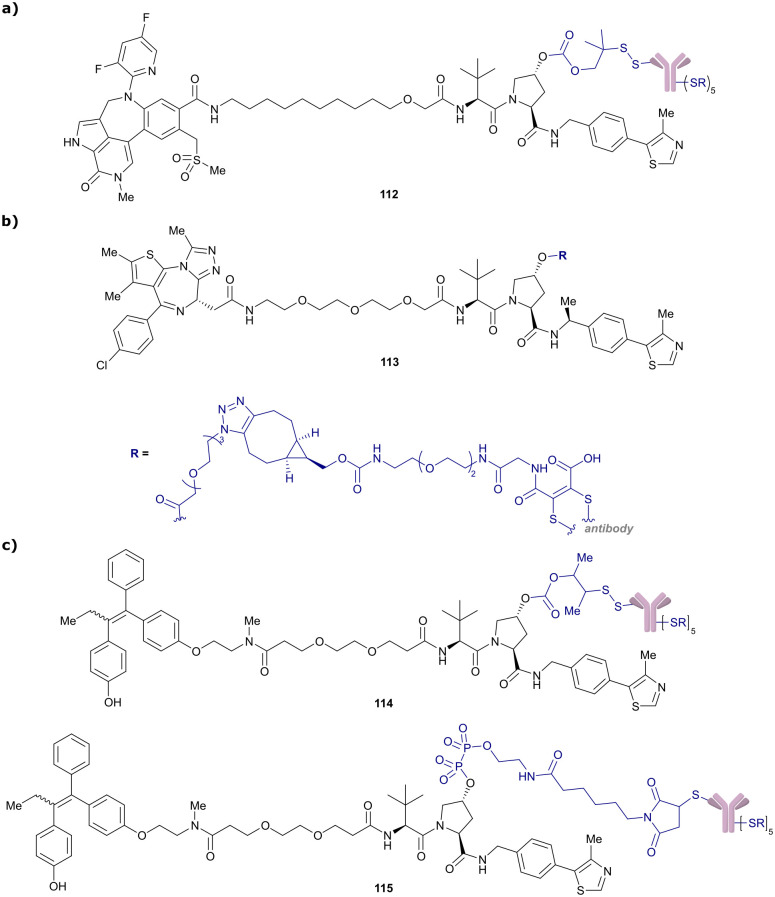
Degrader-antibody conjugates as tissue-selective delivery systems. BET degrader-antibody conjugates 112 and 113 attached using disulfide bonding (a)^202^ or strain-promoted azide–alkyne cycloaddition (b)^201^ and ERα targeting degrader-conjugates featuring disulfide (114) and pyrophosphate (115) linkages to the antibody (c).^204^

Spatial control of PROTAC activity has also been addressed by degrader-antibody conjugates. Adopting antibody-drug conjugate (ADC) technology, tissue-selective delivery systems for both BET^111,201–203^ and ERα^204^ targeting VHL-recruiting PROTACs were recently developed. In an initial report by a Genentech/WuXi collaboration, disulfide-containing linkers, coupled *via* carbonate moieties to Hyp of the VHL binder in BET degrader 112, were used for PROTAC attachment to an anti-CLL1 monoclonal antibody (Fig. 48a). Administration of such degrader-antibody conjugates *in vivo* led to antigen-dependent delivery to acute myeloid leukaemia tumours and induced tumour growth inhibition.^202^ Using the methylated analogue 113 of MZ1 (Fig. 48b) as payload and trastuzumab as HER2+ cells targeting antibody, degrader-antibody conjugates were developed that induced Brd4 degradation selectively in HER2+ breast cancer cell lines, but not in HER2-cell lines, while the corresponding unconjugated PROTAC could not discriminate between the two cell lines. VHL recruitment outside of the target tissue was again prevented by linking the antibody to the Hyp motif of the VHL ligand, using strain-promoted azide–alkyne cycloaddition as conjugation step (Fig. 48b).^201^

In a follow-up in-depth study by the Genentech team, Brd4 targeting degrader-antibody conjugates were systematically optimised for their *in vitro* and *in vivo* bioactivities.^111,203^ While first generation degrader-antibody conjugates using MZ1 derivatives efficiently induced Brd4 degradation, no antiproliferative activity was observed. Besides variation of the conjugation chemistry and antibody-loading,^203^ introduction of a more potent Brd4 binder in the PROTAC scaffold, leading to degrader “Compound 9” (Table 1) whose ternary cocrystal structure bound to VCB and Brd4^BD2^ was solved,^111^ was ultimately necessary to achieve highly cytotoxic payloads. Antibody-degraders based on **Compound**9 induced both potent BET degradation and tumour cell antiproliferation, as well as antigen-dependent antitumour activity in *in vivo* models.^111^ Finally, HER2-selective degrader-antibody conjugates featuring different linkers have been developed for ERα targeting PROTACs.^204^ While cleavage of the disulfide-linked conjugate 114 was expected to release the active degrader after disulfide reduction followed by self-immolation, pyrophosphate diester containing conjugate 115 (Fig. 48c) was designed to undergo phosphatase-mediated hydrolysis to release the active degrader after lysosomal antibody catabolism. Both degrader-antibody conjugates induced significant ERα degradation selectively in HER+ cell lines, consistent with antibody-mediated delivery of the degraders.

The ability to spatiotemporally control the activity of a PROTAC presents an attractive strategy to aid tissue specificity and therapeutic applicability of PROTAC degraders, as potential off-target effects, for example in non-cancerous tissue, are reduced. Due to its pivotal role in VHL binding, Hyp is well placed to be addressed by caging and delivery strategies for VHL-recruiting PROTACs, as functionalisation at Hyp has proven to disable a PROTAC's cellular activity. Capitalising on already established drug-delivery methods, such as caging strategies or antibody-drug conjugates, targeted protein degradation by tissue-selective operating PROTACs presents a highly promising therapeutic strategy.

#### 4.7.2. Tag degradation platforms

Tag degradation platforms can be utilised to assess target protein degradation without embarking in laborious target-selective ligand identification and PROTAC-degrader development. Such degradation platforms enable biological studies and early target validation (or de-validation) using a small molecule, that would otherwise not be possible for targets that lack high-quality small-molecule binders or degraders.^205^ The approach works by fusing the target protein with a protein tag which gets recruited by a tag-selective degrader.^206^ Building on early reports of HaloPROTACs as degraders targeting proteins covalently tagged with a HaloTag7 protein (described in Section 4.1),^100,127^ further tag-based degradation platforms using alternative tagging moieties and tag-targeting degraders have been developed.

**Fig. 49:**
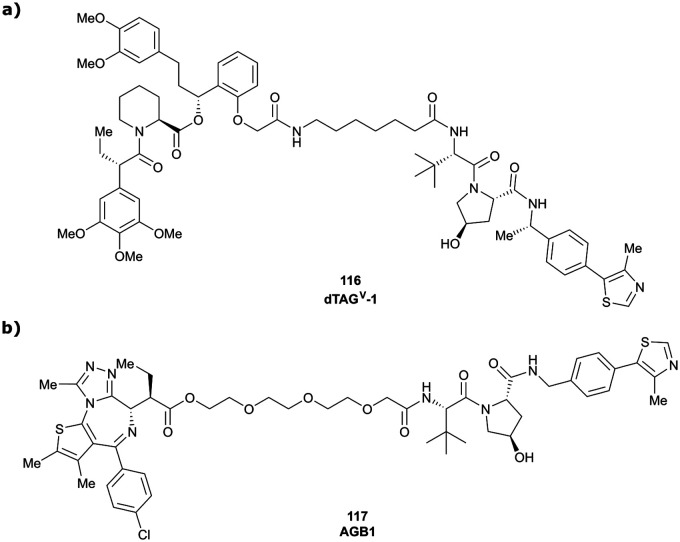
VHL-recruiting degraders used in TAG degradation platforms: FKBP12^F36V^ fusion protein targeting degrader dTAG^V^-1 (116) (a)^129^ and Brd4^BD2 L387A^ (BromoTag) targeting degrader AGB1 (117) (b).^128^

In the dTAG degradation platform developed by the Gray and Bradner laboratories, proteins fused with the synthetic FKBP12^F36V^ protein are targeted by chimeric molecules comprising an FKBP12^F36V^ selective ligand. Initially reported using CRBN-recruiting dTAG degraders,^206^ the dTAG toolbox has been extended to VHL-recruiting dTAG degraders, with dTAG^V^-1 (116, Fig. 49a) as the most potent dTAG degrader with improved pharmacokinetic properties compared to the CRBN-recruiting dTAG degrader molecules.^129^

Using a “bump-and-hole” design approach previously developed on BET bromodomain ligands,^207,208^ the Ciulli laboratory developed the BromoTag platform. They utilised Brd4^BD2^ as the tag bromodomain, by mutating Leu387 of Brd4^BD2^ to Ala (Brd4^BD2 L387A^, referred to as the BromoTag) and derivatised the PROTAC molecule MZ1, by introducing an ethyl group in α-position to the carbonyl functionality of the BET ligand, generating the “bumped” PROTAC AGB1 (117, Fig. 49b).^128^ Following extensive structure-degradation activity relationships, AGB1 was found to induce degradation of a BromoTag’ed Brd2 protein expressed at endogenous protein levels *via* generation of CRISPR knock-in heterozygous cell line, with high efficacy, speed and selectivity. Crucially, no off-target degradation of the untagged native BET proteins Brd2, Brd3 or Brd4 were observed, showing the required high level of selectivity of the new tag system. AGB1 also showed no cytotoxicity in tested cell lines at the operating concentration and featured excellent plasma stability and pharmacokinetic properties in mice, thus qualifying it as a degradation probe for *in vivo* studies.

#### 4.7.3. Nucleotide-containing VHL-recruiting PROTAC technology

**Fig. 50:**
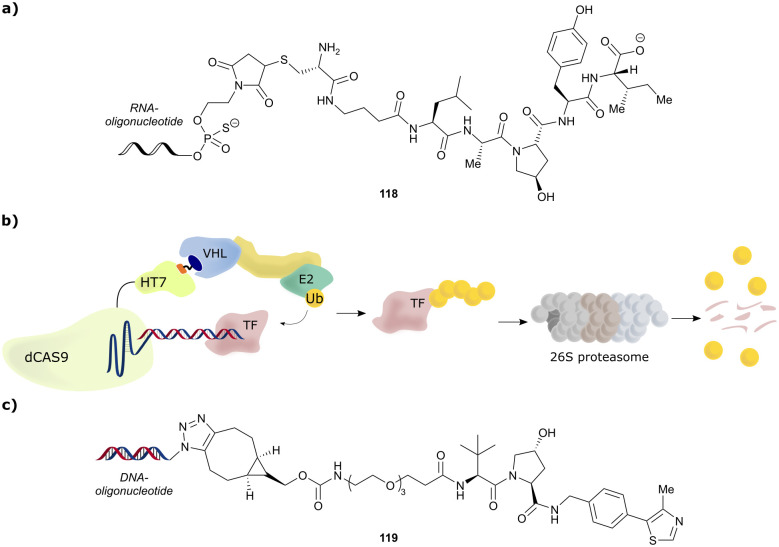
Oligonucleotide-containing PROTACs. Schematic peptide-based RNA-PROTAC 118 (a),^209^ mode of action of TRAFTACs as TF degradation technology (b)^210^ and schematic DNA-nucleotide containing TF degrader 119 (c).^211^

Expanding the toolbox of PROTAC technology, PROTACs comprising short oligonucleotides as POI binding moieties have been introduced recently to specifically target RNA-^209^ and DNA-binding^210–212^ proteins. Using a short, modified RNA oligonucleotide as POI ligand linked to a short peptide derived from HIF-1α (Leu-Ala-[Hyp]-Tyr-Ile) as the VHL ligand, the Hall laboratory pursued the incorporation of oligonucleotides into bifunctional degraders (118, Fig. 50a).^209^ Both their initial peptide-based RNA-PROTACs and an RNA-small-molecule-based degrader using VH032 as the VHL-recruiting ligand, induced partial degradation of the RNA-binding target proteins, stem cell factor LIN28 and a splicing factor RBFOX1. This provided proof of concept for oligonucleotide-containing PROTACs as a viable option for targeting nucleotide-binding proteins.

As transcription factors’ (TFs) function is triggered by binding of specific DNA sequences, such DNA sequences are prone to serve as highly selective ligands in chimeric degraders targeting otherwise difficult-to-drug TFs. Relying on this concept, the Crews laboratory reported TF degraders, so-called TRAFTACs, consisting of a TF-specific double-stranded DNA sequence covalently linked to Cas9-CRISPR-binding RNA recruiting a dCas9-Halotag7 fusion protein which can be targeted by HaloPROTACs. By increasing the linker length within the HaloPROTAC, previously reported direct degradation of the HaloTag7 fusion protein (Section 4.1) could be avoided, repurposing the fusion protein as a connector between the VHL E3 ligase and the DNA oligonucleotide (Fig. 50b), ultimately inducing target TF degradation.^210^

Besides this “indirect” approach to connect a TF recruiting oligonucleotide with an E3 ligase recruiter, TF degradation technology based on covalent linkage of DNA oligonucleotide moieties with small-molecule E3 recruiting ligands has also been established. The laboratories of Jin and Wei designed bivalent TF degraders by conjugating azide-modified DNA oligomers *via* strain-promoted azide-alkyne cycloaddition to bicyclooctyne-modified VHL ligands (Fig. 50c).^211^ Direct incorporation of the DNA oligonucleotide impeded neither target nor E3 ligase binding, and ≥50% reduction target TF levels was achieved with bivalent TF degraders such as 119. Using the same concept, the laboratories of Li and Huang reported bivalent VHL-recruiting TF degraders, in which phosphoramidates attached to the 5′ terminus of a 19-mer DNA strand serve as anchor for linker attachment to VH032 as VHL-recruiting ligand.^212^

Though present oligonucleotide-based degraders merely serve as proof-of-concept inducing only moderate target degradation so far, utilising short oligonucleotides as POI recruiting elements has potential as a strategy to address otherwise elusive targets. Beneficially, high DNA binding specificity of TFs is expected to reduce potential off-target effects and avoids otherwise required ligand optimisation that can be laborious and low-success for such poorly ligandable targets as TFs.

## 5. Concluding remarks

Fragment-based design has proven pivotal to the discovery of early VHL binders and their subsequent rational structure-guided optimisation into potent VHL inhibitors. Besides their obvious application as chemical probes for disrupting the VHL/HIF-1α interaction and consequently upregulating HIF-1α-dependent processes, VHL inhibitors have served as a platform for further chemical developments, for example of VHL-recruiting fluorescence or NMR probes for biophysical assays. In particular, the development of high affinity, high specificity small-molecule ligands for VHL paved the way for the development of PROTACs, leading to the establishment of many small-molecule VHL-recruiting PROTACs efficiently targeting a multitude of different proteins for degradation. Modulation of the VHL ligand's scaffold and linker tethering vector have in many cases proven to be critical for achieving PROTAC's efficacy and selectivity against specific targets. Though N-terminally amide-bond tethered VHL-recruiting PROTACs have predominated to date, alternative exit vectors are on the rise and are proving increasingly successful at generating fast, potent, and effective PROTAC degraders for multiple applications, and with favourable physicochemical properties. In this light, it seems highly recommended to assess several exit vectors on the VHL binder in the early-stage development of VHL-recruiting PROTACs prior to fine-tuning by further subtle structural modifications, such as introducing a methyl group in the RHS benzylic position or variation of the substituent at the LHS *ipso*-cyclopropyl position of VH298. We anticipate exciting future developments of novel multi-functional molecules and modalities based on the VHL ligand. Chemistries for macrocyclic, trivalent, and cleavable VHL-based molecules are some of the examples that will no doubt inspire more chemical creativity to build on the VHL ligand with multiple unforeseen applications in chemical biology and drug discovery.

The recent disclosure that most of the first clinical PROTAC drugs are CRBN-based has led to a widespread belief that CRBN-recruiting PROTACs might be more suitable than VHL-recruiting PROTACs for therapeutic application. This is reflected by a considerable higher number of orally bioavailable CRBN-based PROTAC degraders currently investigated in clinical trials.^213^ The lower molecular weight of common CRBN ligands compared to VHL ligands, along with their more favourable physicochemical properties such as lower HBD and HBA counts and lower lipophilicity, is believed to offer a more “drug-like” starting point for degrader development.^174^ However, recent advances in the design of orally bioavailable VHL-based PROTAC degraders,^103,126,173^ together with the evidence that they can achieve exposure in the brain,^126^ and combining PROTACs with established drug-delivery methods, such as ADCs^111,201–204^ or caging strategies,^191–194,196^ represent important milestones on the quest towards conveniently administrable VHL-recruiting PROTACs as therapeutic modalities to impact unmet medical needs. Though first studies focussing on BET degrading PROTACs disclosed both acquired and intrinsic resistance mechanisms towards both CRBN- and VHL-recruiting PROTACs,^214,215^ the essential role of VHL for survival of many cancerous cell lines results in a lower fraction of disruptive mutations on the substrate receptor VHL compared to CRBN.^216^ Instead, Cul2 loss has been identified as primary resistance mechanism induced by MZ1 as exemplary VHL-recruiting degrader.^214,215,217^ Interestingly, MZ1-resistant cells have shown to be still sensitive towards μM concentrations of the more potent VHL-based BET protein degrader ARV-771,^214^ and to exhibit undiminished sensitivity towards CRBN-based BET degraders.^214,215,217^ As such, though resistance against PROTAC degraders can be acquired, this resistance can be mitigated by targeting the POI using either a different, more potent PROTAC for the same ligase, or degraders for the same target recruiting alternative E3 ligases. Together, these observations and other mounting evidence highlight the importance and benefit of developing multiple degrader series with different characteristics, such as varying the recruited E3 ligase, even for the same target, as a strategy to maximise scope and minimise risks. It follows, and could be envisioned, that first-in-class and best-in-class degrader drugs could well differentiate based on their E3 ligases and chemistries. The future is therefore bright for VHL-based PROTAC degraders, and the community will watch with trepidation their progress through clinical trials.

## Abbreviation list

α: Cooperativity factor
ADC: Antibody-drug conjugate
Ala: Alanine
ALK: Anaplastic lymphoma kinase
AR: Androgen receptor
Arg: Arginine
Asn: Asparagine
Bcl-2: B-Cell lymphoma 2
Bcl-xL: B-Cell lymphoma extra large
BD: Bromodomain
BET: Bromo- and extra-terminal (protein)
Boc: *tert*-Butoxycarbonyl (protecting group)
BODIPY-FL: Difluoro[methyl 5-methyl-2-[(5-methyl-2*H*-pyrrol-2-ylidene)methyl]-1*H*-pyrrole-3-acetato-N1,N2]boron
Brd4: Bromodomain containing protein 4
Brd4^BD2^: Second bromodomain of Brd4
BTK: Bruton-Tyrosinkinase
*C*_2_
: R_2_ contrast
CAND1: Cullin-associated NEDD8-dissociated protein 1
ccRCCs: Clear-cell renal cell carcinomas
CDK4/6: Cyclin-dependent kinase 4/6
CETSA: Cellular thermal shift assay
cIAP: Cellular inhibitor of apoptosis protein 1
CODD: C-terminal oxygen destruction domain
CRBN: Cereblon
CRL2^VHL^: Cullin2 RING-VHL
Cul2/4: Cullin2/4
CSN: COP9 signalosome
Da: Dalton
DC_50_: Half-degrading concentration
DCAF11/16: (DDB1)-Cul4 associated factor 11/16
DDB: Damage-specific DNA binding protein 1
DEACM: Diethylamino coumarin
D_max_: Maximal degradation
DMNB: 4,5-Dimethoxy-2-nitrobenzyl
DSF: Differential scanning fluorimetry
EDG: Electron donating group
EGFR: Epidermal growth factor receptor
EloB: Elongin B
EloC: Elongin C
ERα: Oestrogen receptor α
ERRα: Oestrogen-related receptor α
EWG: Electron withdrawing group
FAK: Focal adhesion kinase
FBLD: Fragment-based lead discovery
FKBP12: FK506 binding protein
FOLR1: Folate receptor α
FP: Fluorescence polarisation
GFP: Green fluorescent protein
Gln: Glutamine
GPCRs: G-protein coupled receptors
HBA: Hydrogen bond acceptor
HBD: Hydrogen bond donor
HER+/−: Human epidermal growth factor positive/negative
HIFs: Hypoxia inducible factors
HIF-1α: Hypoxia-inducible factor 1-α
HIF-1α-OH: Hydroxylated HIF-1α
His: Histidine
Homo-PROTACs: Homo-bivalent PROTACs
HTS: High-throughput screening
Hyp: Hydroxyproline
IC_50_: Half-maximal inhibitory concentration
IKK: Inhibitor of kappa B kinase
Ile: Isoleucine
IMHBs: Intramolecular hydrogen bonds
ITC: Isothermal titration calorimetry
*K*_d_
: Dissociation constant
KEAP1: Kelch-like ECH-associated protein 1
LHS: Left-hand side
LE: Ligand efficiency
Leu: Leucine
LLE: Lipophilic ligand efficiency
LRRK2: Leucine rich repeat kinase 2
MAPK: Mitogen-activated protein kinase
MDM2: Mouse double minute 2 (E3 ligase)
MEK1/2: Mitogen-activated protein kinase 1/2
Met: Methionine
MetAP-2: Methionine aminopeptidase 2
mRNA: Messenger ribonucleic acid
MS: Mass spectrometry
NMR: Nuclear magnetic resonance
NQO1: NAD(P)H quinone dehydrogenase 1
ODDs: Oxygen-dependent degradation domains
PARP: Poly(ADP-ribose)polymerase
PCCs: Phaeochromocytomas
PEG: Polyethylene glycol
PHD: Prolyl hydroxylase domain (enzyme)
Phe: Phenylalanine
POI: Protein of interest
PPIs: Protein–protein interactions
Pro: Proline
PROTAC: Proteolysis targeting chimera
QM/MM: Quantum mechanics/molecular mechanics
R_2_: Relaxation rate 2 (NMR parameter)
RHS: Right-hand side
RING: Really interesting new gene
RIPK2: Receptor-interacting serine/threonine-protein kinase 2
RNF4/114: RING-type zinc-finger protein 4/114
Ro3: Rule-of-3
ROS: Reactive oxygen species
SAR: Structure–activity relationship
Ser: Serine
SFC: Supercritical fluid chromatography
SGC: Structural genomics consortium
SGK: Serum/glucocorticoid-inducible protein kinase
SPR: Surface plasmon resonance
Tat: Transactivating transcriptional activator
*t*Bu: *tert*-Butyl
*tert*-Leu: *tert*-Leucine
TBK1: TANK-binding kinase 1
TDSC: Tendon-derived stem cell
TF: Transcription factor
TMT: Tandem mass tag
TPD: Targeted protein degradation
TR-FRET: Time-resolved fluorescence resonance energy-transfer
Trp: Tryptophan
Tyr: Tyrosine
UPS: Ubiquitin–proteasome system
VCB: VHL-ElonginC-ElonginB protein complex
VHL: von Hippel-Lindau (protein)
*vhl*
: von Hippel-Lindau (gene)
WaterLOGSY: Water-ligand observed *via* gradient spectroscopy (NMR method)
WDR5: WD40 repeat-containing protein 5

## Conflicts of interest

The Ciulli laboratory receives or has received sponsored research support from Almirall, Amgen, Amphista Therapeutics, Boehringer Ingelheim, Eisai, Merck KGaA, Nurix Therapeutics, Ono Pharmaceutical and Tocris-BioTechne. A. C. is a scientific founder, advisor, and shareholder of Amphista Therapeutics, a company that is developing targeted protein degradation therapeutic platforms. C. D. reports no competing interest.

## Supplementary Material
